# Discovery and Characterization of Novel IKZF1/3 Glue Degraders against Multiple Hematological Cancer Cell Lines

**DOI:** 10.32604/or.2025.065123

**Published:** 2025-09-26

**Authors:** Ting Wei, Pengli Wei, Yalei Wang, Yaqiu Mao, Jian Yan, Xiaotong Hu, Zhenze Qi, Xu Cai, Changkai Jia, Zhiyuan Zhao, Bingkun Li, Min Qiao, Yaxin Zou, Tingting Yang, Shiyang Sun, Xuesong Feng, Pengyun Li, Hongzhou Shang, Zhibing Zheng

**Affiliations:** 1 College of Chemical Engineering, North China University of Science and Technology, Tangshan, 063210, China; 2 National Engineering Research Center for Strategic Drugs, Beijing Institute of Pharmacology and Toxicology, Beijing, 100850, China; 3 School of Pharmacy, China Medical University, Shenyang, 110122, China; 4 State Key Laboratory of Natural and Biomimetic Drugs, School of Pharmaceutical Sciences, Peking University, Beijing, 100191, China

**Keywords:** Hematological cancer, cereblon ligands (CRBN ligands), molecular docking, Ikaros family zinc finger proteins 1 and 3 (IKZF1/3), antitumor evaluation

## Abstract

**Objectives:**

Immunomodulatory drugs (IMiDs), functioning as molecular glue degraders, have been approved for treating various hematological malignancies; however, the inevitable acquired drug resistance resulting from their skeletal similarity and hematological toxicities poses significant obstacles to their clinical treatment. The study aimed to develop degraders with potent efficiency and low toxicity.

**Methods:**

Phenotypic profiling, elaborate structure-activity relationships (SAR), rational drug design and degradation profiles investigations, quantitative proteomics analysis and cell-based functional studies, and pharmacokinetic studies were conducted to develop more potent degraders.

**Results:**

This study developed novel CRBN-binding moieties through methylene deletion in lenalidomide’s isoindole core. Lead compounds MGD-A7 and MGD-C9 demonstrated superior antiproliferative efficacy vs. IMiDs, with submicromolar potency. MGD-A7 and MGD-C9 significantly and selectively induced the degradation of Ikaros Family Zinc Finger Proteins 1 and 3 (IKZF1/3) with nanomolar potency via a CRBN-dependent pathway. Mechanistically, MGD-A7 and MGD-C9 dramatically induced cell apoptosis and G1 cell cycle arrest and MGD-C9 exhibited favorable pharmacokinetic properties *in vivo*. Furthermore, MGD-C9 exhibited significant synergistic effects with standard-of-care agents in various hematological malignancy cells.

**Conclusions:**

These results indicate that MGD-C9 could act as a highly effective CRBN ligand and is expected to become a candidate drug for the treatment of hematological malignancies.

## 1 Introduction

Targeted protein degradation (TPD), a cutting-edge technology, harnesses the ubiquitin-proteasome and the autophagy-lysosome system inherent in the human body to specifically target and degrade disease-related proteins, thereby facilitating disease treatment [1,2]. Unlike traditional protein regulators, which rely on the high-affinity occupation of specific hydrophobic pockets on the target protein surface (occupancy-driven), TPD can effectively eliminate disease-causing proteins that are difficult to target with conventional drugs. Additionally, due to its unique pharmacological mechanism, TPD shows potential in overcoming the drug resistance problem associated with existing drugs [3,4]. Immunomodulatory drugs (IMiDs), including thalidomide, lenalidomide, and pomalidomide, have been approved for clinical hematological malignancies, such as multiple myeloma (MM), acute myeloid leukemia (AML), and myelodysplastic syndrome (MDS) [5]. Increasing studies have shown that these compounds share a common glutarimide moiety, which binds to a shallow pocket with a phenylalanine side-chain base formed by three tryptophan residues in cereblon (CRBN). They act as molecular glue through the Cullin 4 (CUL4)-DNA Damage-Binding Protein 1 (DDB1)-Ring-Box Protein 1 (RBX1)-CRBN E3 ligase complex to induce the ubiquitination of multiple substrate proteins (neosubstrates), such as Ikaros family of zinc finger transcription factors (IKZF1/2/3), casein kinase 1α (CK1α) and G1 to S phase transition 1 gene (GSPT1), thus triggering a series of pharmacological effects [6].

**Figure 1:**
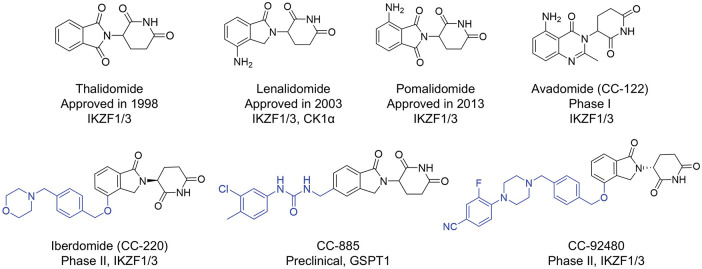
The representative CRBN E3 ligase ligands and their primary degradation substrates

However, to date, nearly all CRBN ligands have been developed based on thalidomide. Their skeletal similarity and small structural differences will inevitably lead to the emergence of drug resistance [7]. As reported, the drug-resistance phenomenon during the treatment of MM with IMiDs can be addressed by developing novel CRBN ligands. Several optimization strategies have been explored to develop CRBN ligands [8]. Avadomide (CC-122), an IKZF1/3 degrader, has been developed by replacing the phthalimide ring with quinazoline, exhibiting greater potency than lenalidomide in the degradation of IKZF1/3 [9]. Avadomide exerts potent inhibitory effects on the proliferation of diffuse large B-cell lymphoma (DLBCL) cells *in vitro* and *in vivo* [9], and has been investigated for its dose-expansion of safety and clinical activity in patients with novo relapsed or refractory DLBCL and transformed lymphoma in a phase I study [10]. Other lenalidomide-like compounds, such as Iberdomide (CC-220), CC-885 and mezigdomide (CC-92480), are derived by chain extension with specific substituents on the benzene ring of phthalimide, forming more interactions with CRBN (Fig. 1) [6]. Compared with traditional lenalidomide-like drugs, the activities of these molecules have been significantly improved both *in vitro* and *in vivo*. These compounds are currently in the clinical trial stage and are being investigated for relapsed or refractory multiple myeloma (R/R MM), leukemia, DLBCL treatment, and other indications [10,11]. Nevertheless, during the clinical trial stage, common toxic and side effects, such as neutropenia, anemia, and thrombocytopenia, have been observed [12]. Furthermore, the binding motifs of CRBN have emerged as highly valuable scaffolds for E3 ligase recruitment in several reported heterobifunctional degraders, including proteolysis targeting chimeras (PROTACs) currently under clinical study, such as ARV-471, ARV-110, and NX-2127 [13–15], as well as PROTACs targeting oncoprotein such as epidermal growth factor receptor (EGFR) [16], Kirsten rat sarcoma (KRAS) [17], c-mesenchymal-to-epithelial transition (c-MET) [18] and Bruton’s tyrosine kinase (BTK) [19] in preclinical studies. Consequently, it’s urgent to develop novel and highly potent CRBN ligands that possess low toxicity, which could help overcome drug resistance and facilitate the advancement of both PROTAC and molecular glue technologies.

Since several IMiDs-CRBN co-crystal studies have validated the pivotal role of the glutarimide moiety in IMiDs’ anchoring to CRBN through hydrogen bond interactions with CRBN residues His380 and Trp382, respectively [20–22]. Recent studies on CRBN ligands have shown that CRBN ligands could be obtained by simply modifying the methylene or carbonyl group in the five-membered ring, where the indolizine ketone or benzoyl imide core is connected to the imidazole pyrimidine part [23]. The study presents the rational design, synthesis, and comprehensive evaluation of a series of CRBN ligands. These ligands feature a novel skeleton that was conceived by simply deleting the methylene in the isoindole cores of lenalidomide across a range of MM, AML, and DLBCL cell lines. By extensively exploring structure-activity relationships (SAR) and protein degradation mechanisms, the study identified MGD-C9, a highly effective IKZF1/3 degrader, which holds great promise as a candidate for treating hematological cancers.

## 2 Materials and Methods

### 2.1 Chemistry: General Methods and Synthesis

All reagents and solvents were obtained from commercial sources. They were used right after arrival without any further purification procedures. The reactions were carried out under precisely controlled pressure and temperature conditions. Thin-layer chromatography (TLC) was performed on precoated silica gel plates (Qingdao Haiyang Chemical Co., Qingdao, China). The silica gel used had a particle size ranging from 40–63 μm. The purity of all compounds was determined by High Performance Liquid Chromatography (HPLC) analysis. The mobile phase was set as a gradient of 20% acetonitrile rinse over 30 min, with a flow rate of 1 mL/min. At a wavelength of 254 nm, the purity of the assay exceeded 95%. The ^1^H and ^13^C nuclear magnetic resonance (NMR) spectra were tested using an AVANCE NEO 600 MHz mass spectrometer (Bruker, Bilerika, MA, USA). For ^1^H, the spectrometer operated at 600 MHz, and for ^13^C, it operated at 151 MHz. Moreover, JNM-ECZ400S spectrometers (JEOL, Tokyo Metropolis, Akishima-shi, Japan) were also employed for NMR measurements. High-resolution mass spectrometry (HRMS) data were gathered using an AB Sciex API 3000™ LC/MS system (Sciex LLC, Framingham, MA, USA).

The detailed information on the synthesis steps and properties of these compounds is provided in the Supplementary Materials Sections 2 and 3.

### 2.2 Biological Assays

#### 2.2.1 Reagents and Antibodies

Thalidomide (#T0213), pomalidomide (#T2384) and lenalidomide (#T1642) gemcitabine (#T0251), decitabine (#T1508), etoposide (#T0132), cytarabine (#T1272), gilteritinb (#T4409), sorafenib (#T0093L), venetoclax (#T2119), pirtobrutinib (#T36287), avadomide (#T3549) and ivosidenib (#T3617) were procured from Targetmol, Boston, MA, USA. All the drugs were dissolved in dimethylsulfoxide (DMSO) within the range of 10–200 mM. These solutions were then stored at −30°C as stock solutions.

The following primary antibodies were used: CRBN (#71810, 1:1000) and IKZF3 (#15103, 1:500) were all from Cell Signaling Technology, Boston, MA, USA; CK1α (#ab108296, 1:1000) were purchased from Abcam, MA, USA; IKZF1 (#12016-1-AP, 1:1000), GSPT1 (#10763-1-AP, 1:500) was purchased from Proteintech, Rosemont, IL, USA; Anti-rabbit IgG (HRP-conjugated, Cell Signaling Technology, #7074, 1:3000), anti-mouse IgG (HRP-conjugated, Cell Signaling Technology, #7076, 1:3000) were used as secondary antibodies.

#### 2.2.2 Cell Culture and Transfection

HEK293T (293T) cells (#CRL-3216) were cultured in high-glucose Dulbecco’s Modified Eagle’s Medium (DMEM, #SH30022.01; Hyclone, Logan, UT, USA). NCI-H929, OPM-2, U937, Ocl-Ly3, RPMI-8226, MOLM-13, Skm-1, SU-DHL-4, TMD8, U2932, and WSU-DLCL-2 cells were grown in Roswell Park Memorial Institute Medium (RPMI, #SH30809.01; Hyclone, Logan, UT, USA). MV-4-11 and KG-1 cells were cultured in Iscove’s Modified Dulbecco’s Medium (IMDM, #SH30228.01; Hyclone). The cells were maintained in media that contained 10% fetal bovine serum (FBS, #16000-044; Gibco^TM^, Grand Island, NY, USA), 100 µg/mL penicillin, and 100 µg/mL streptomycin (#15140122, Thermo Fisher Scientific, MA, USA). All these cell lines were sourced from the American Type Culture Collection (ATCC, Manassas, VA, USA). MUTZ-1 cell line was sourced from the BeNa Culture Collection (BNCC, Beijing, China), which was grown in RPMI Medium. The culture environment was set at 37°C in a humidified incubator with a 5% CO_2_ environment. All cell lines underwent mycoplasma testing, and the results showed no mycoplasma contamination. As described in our previous research, HEK293T cells that stably expressed IKZF1-, IKZF2-, IKZF3-, GSPT1-, and CK1α-HiBiT were established [23].

#### 2.2.3 Cell Viability and Drug Synergy Assays

Multiple cell lines (NCI-H929, OPM-2, U937, Ocl-Ly3, RPMI-8226, MOLM-13, Skm-1, MUTZ-1, SU-DHL-4, TMD8, U2932, WSU-DLCL-2, MV-4-11 and KG-1 cells) were plated into 96-well plates. The plated at a density of 15,000 cells per well, and each well contained 100 µL of medium. Subsequently, different compounds (MGD-AX, MGD-BX, MGD-CX) were added to each well. These compounds’ concentrations were set at 0, 0.01, 0.033, 0.1, 0.33, 1, 3.3, 10 and 33 μM, and the cells were incubated with the compounds for 96 h. To evaluate the antiproliferative effects of these compounds, the Cell Counting Kit-8 (CCK-8, #CX007, Epizyme, Shanghai, China) was used. All procedures were carried out in strict accordance with the manufacturer’s instructions (Dojindo Laboratories, Mamoto Ken, Japan). The half maximal inhibitory concentrations (IC_50_) values, which represent the concentrations of the test compounds that can inhibit 50% of cell activity, were calculated using GraphPad Prism 8.0 (GraphPad Software Inc., San Diego, CA, USA).

In the drug synergy experiments, cells including NCI-H929, OPM-2, MV-4-11, KG-1, Ocl-Ly3 and U2932. They were inoculated into 96-well plates, and ultimately, the volume of the culture medium in each well remained at 100 μL, with the cell density in each well at 12,000 cells. Subsequently, the cells were treated with either MGD-C9 or drugs such as Gemcitabine, Decitabine, Etoposide, Cytarabine, Venetoclax, Gilteritinib, Pirtobrutinib, Sorafenib, and Ivosidenib as single agents or in combinations. These treatments were incubated for 96 h at concentrations of 0, 0.01, 0.033, 0.1, 0.33, 1, 3.3, 10, and 33 μM. The antiproliferative effects of these compounds were evaluated by the CCK-8 assay according to the manufacturer’s protocol. The synergy effect of drug pairs. Reflected by the Combinational Index (CI) value, was quantitatively determined by the Chou-Talalay equation [24], which enabled a quantitative evaluation of the interactions among the drugs. CI < 1 indicates synergism; CI > 1 indicates antagonism.

#### 2.2.4 Molecular Docking

The crystal structure of the CRBN and IKZF1/3 complex from the entry 8D7Z in the Protein Data Bank (PDB, https://www.rcsb.org/) [22]. To get the proteins ready for further analysis, the Protein Preparation Wizard in Maestro (2022-4, Schrödinger, NY, USA) was applied. Subsequently, ‘Prime’ was used to add any missing side-chains and loops to the protein structure to obtain a more comprehensive and precise model. The ionization state of the ligand, appropriate for a pH range of 7.0 ± 2.0, was predicted using ‘Epik’ in combination with the ‘OPLS4’ force field. After that, Receptor Grid Generation was performed using the crystal structure to set up the grids for the binding site. During this step, His378 and Trp380 were specifically set as H-bond constraints. Finally, the ligands were docked into the CRBN-IKZF1/3 complex using ‘Glide’ Standard Precision (SP) with the default settings.

#### 2.2.5 Immunoblot Analysis

Cells (NCI-H929 and MV-4-11) were plated into 6-well plates. They were then treated with different concentrations (0, 0.01, 0.1, 1, 10 μM) of the compounds (MGD-A7, MGD-C9) or the designated time periods. Afterward, the cells were washed with phosphate-buffered saline (PBS, pH = 7.2–7.4, 1×). Next, they were lysed using RIPA buffer (#R0278-500ML, Sigma-Aldrich, MO, USA), and the cell lysates were precisely measured. Following this, the total protein lysates underwent 10% sodium dodecyl sulfate-polyacrylamide gel electrophoresis (SDS-PAGE). This method efficiently separated the proteins based on their molecular weights. Subsequently, the separated proteins were transferred onto a nitrocellulose membrane (#HATF00010, Promega, Madison, WI, USA). The membranes were then successively incubated with the indicated primary antibodies. These antibodies were diluted in 5% bovine serum albumin (BSA) in Tris-buffered saline with Tween 20 (TBST, #TF103; Epizyme, Shanghai, China). The incubation was conducted overnight at 4°C with gentle agitation. The secondary antibodies were also diluted in 5% BSA in TBST (#TF103; Epizyme, Shanghai, China). The membranes were incubated with the corresponding secondary antibodies according to the manufacturer’s suggestions for 1 h at room temperature. Finally, after thorough washing with TBST, the membranes were developed using an enhanced chemiluminescence (ECL) substrate (#WBULS0100, Sigma-Aldrich). The chemiluminescent signal was detected, and membrane images were captured with an Imaging system (Bio-Rad, Hercules, CA, USA). ImageJ analysis software (V1.8.0.112, National Institutes of Health, NIH, Bethesda, MD, USA) was employed to analyze the intensity of protein bands, aiming to quantify the relative expression levels of the target proteins. The half-maximal degradation concentration (DC_50_) value, which indicates the concentrations of test compounds showing 50% protein degradation efficacy, was calculated using GraphPad Prism 8.0.

#### 2.2.6 Quantitative Degradation Assay Using the HiBiT System

An *in vitro* quantitative degradation assay utilizing the HiBiT system was carried out as previously described [23]. To evaluate the degradation of IKZF1, IKZF2, and IKZF3 proteins, HEK293T cell lines stably expressing IKZF1-, IKZF2-, IKZF3-, GSPT1- or CK1α-HiBiT were cultured in 96-well plates at 12,000 cells per well. The cells were treated with different concentrations of the test compounds (MGD-A7 and MGD-C9) with concentrations of 0, 0.01, 0.033, 0.1, 0.33, 1, 3.3, 10 and 33 μM for the specified time intervals. After the treatment, cell lysis was carried out using the Nano-Glo HiBiT Lytic Detection System (#N3040, Promega). All lysis operations were strictly carried out according to the manufacturer’s specific protocol. Then, the SpectraMax iD3 instrument (Molecular Devices, San Jose, CA, USA) was used to detect the luminescent signals released by the HiBiT-tagged neosubstrates.

CRBN knockout (CRBN^−/−^) NCI-H929 and MV-4-11 cells

CRBN^−/−^ NCI-H929 and MV-4-11 cells were seeded at a density of 1 × 10^6^ cells/well in 6-well tissue culture-treated plates. According to the manufacturer’s protocol (Santa Cruz Biotechnology, Inc, Dallas, TX, USA), CRBN CRISPR/Cas9 knockout Plasmid (#sc-412142), CRBN HDR plasmid (#sc-412142-HDR), UltraCruz^®^ Transfection Reagent (#sc-395739) were pre-mixed in Plasmid Transfection Medium (#sc-108062) at a 1:1:2 mass ratio, and cells were transiently transfected in a 37°C, 5% CO_2_ incubator. After 72 h of infection, the medium was replaced with complete medium containing 3 μg/mL puromycin for 5–7 days. On day 7, GFP-positive cells were sorted as single cells into 96-well plates. Isolated single-cell clones were screened via western blot analysis, using wild-type NCI-H929 or MV-4-11 cells as positive controls.

### 2.3 MS-Based Proteomic Analysis

#### 2.3.1 Sample Preparation

NCI-H929 cells were treated with either 10 μM DMSO or 10 μM MGD-C9 for 4 h. Post-treatment, the cells were washed with PBS (pH = 7.2–7.4, 1×) and then lysed in 8M Urea (UA) (comprising 8M urea, 100 mM Tris-HCl, pH = 8.0). Subsequently, the cell lysate was subjected to centrifugation on a high-speed freezing microcentrifuge (#CF1524R; SCILOGEX, Meriden, CT, USA) at 120,000 *m*/*z* 200, and the supernatant was collected. Subsequently, the collected supernatant was treated with 10 mM tris (2-carboxyethyl) phosphine hydrochloride (TCEP) to reduce it for 30 min. Following this, cysteine alkylation was performed using 50 mM 2-chloroacetamide (CAA) for an additional 30 min. The denatured proteins were then digested with trypsin (1 μg/μL) at an enzyme-to-protein ratio of 1:50 and incubated at 37°C overnight. The reaction was stopped by adding 0.1% formic acid (FA). The samples were desalted using 100 μL pipette tips packed with C18 discs (3M, Saint Paul, MN, USA). Prior to sample loading, the tips were conditioned sequentially with 80 μL of acetonitrile (ACN), followed by 80 μL of a 1:1 (v/v) mixture of H_2_O and ACN, and finally with 80 μL of H_2_O. The conditioned tips were loaded with peptides. Then, they were washed twice with 80 μL of H_2_O. After that, the peptides were eluted twice using 80 μL of a solution composed of 50% water and 50% acetonitrile (ACN). The resulting eluates were vacuum-dried and then reconstituted in 0.1% formic acid for the subsequent LC-MS/MS analysis.

#### 2.3.2 Liquid Chromatography-Tandem Mass Spectrometry (LC–MS/MS) Analysis

The peptide mixtures were analyzed using an EASY-nLC 1200 liquid chromatography system (Thermo Fisher Scientific, WLM, MA, USA) equipped with a custom-made 15 cm C18 column (150 μm ID, 1.9 μm particle size, 100 Å pore size). Peptide separation was performed over a 75-min gradient at a constant flow rate of 600 nL/min. The gradient profile was as follows: 4%–7% buffer B (1 min), 7%–13% buffer B (6 min), 13%–25% buffer B (38 min), 25%–45% buffer B (22 min), 45%–95% buffer B (1 min), followed 95% buffer B (7-min). The buffers used were Buffer A (0.1% formic acid) and Buffer B (0.1% formic acid in 80% acetonitrile). The peptide mixtures were then analyzed using a Q Exactive HF mass spectrometer (Thermo Fisher Scientific). MS data were acquired in data-dependent acquisition (DDA) mode with a dynamic exclusion duration of 15 s. MS1 scans were acquired in positive-ion mode across a mass-to-charge (*m*/*z*) range of 300–1400, with a resolution of 120,000 (at *m*/*z* 200) and a maximum injection time of 50 ms. Precursor ions were fragmented using higher-energy collision dissociation (HCD) at 27% normalized collision energy. MS2 spectra were collected with an AGC target of 5.0 × 10^4^ and a maximum injection time of 20 ms.

#### 2.3.3 MS Data Analysis

Label-free MS raw data were processed using MaxQuant (v2.4) against the UniProt Human database (https://www.uniprot.org/, downloaded May 2024; 20,434 entries). A strict false discovery rate (FDR) threshold of ≤0.01 was applied at the spectra, protein, and modification levels. Contaminants and reverse database hits were excluded. Default settings were used for all other parameters. Protein groups output by MaxQuant were further analyzed in R (v4.2.1). Student’s *t*-test, and *p*-values < 0.01 were considered statistically significant for statistical analyses and a volcano plot was generated with GraphPad Prism 8.0.

#### 2.3.4 Data Availability

MS proteomics data were deposited to the ProteomeXchange Consortium (https://proteomecentral.proteomexchange.org (accessed on 11 June 2025)) via iProX (dataset identifier: PXD061290) [25,26].

### 2.4 Cell Apoptosis Assay and Cell Cycle Assay

Cells (NCI-H929 and MV-4-11) were seeded at a density of 1 × 10^6^ cells per well into 6-well dishes. After that, the cells were cultured in medium supplemented with different concentrations of compounds (MGD-A7, MGD-C9 and Pomalidomide) with concentrations of 1 and 10 μM, respectively, for 48 h. Apoptosis was assessed using an Annexin V-FITC kit (#ab14085; Abcam, MA, USA) on a FACS Calibur Flow Cytometer (Becton Dickinson, Franklin Lakes, NJ, USA). For cell cycle analysis, cells were fixed in 75% ethanol (−20°C/overnight), washed with PBS (pH = 7.2–7.4, 1×), treated with RNase A (0.2 mg/mL, 37°C/30 min), stained with PI (10 μg/mL), and analyzed on the same instrument. Finally, the samples were analyzed using a FACS Calibur Flow Cytometer.

### 2.5 The Pharmacokinetic Study In Vivo

BALB/c mice weighing 19–21 g were procured from Beijing Vital River Laboratory Animal Technology Co., Ltd. (Beijing, China). The animals were housed under conditions of constant temperature and humidity, with a 12-h light-dark cycle for lighting. The mice had free access to water and food but were fasted for 12 h before intragastric administration. This study has been approved by the Animal Care and Use Committee Ethics Committee of the National Beijing Center for Drug Safety Evaluation and Research; the approval number is IACUC-DWZX-2024-P557.

Six male BALB/c mice were randomly assigned to two groups, with three mice in each group. MGD-C9 was administered via intravenous (i.v., 2 mg/kg in a solution of 10% DMSO, 2% Tween 80% and 88% H_2_O) and oral (p.o., 50 mg/kg in the same vehicle) routes to assess its pharmacokinetic (PK) characteristics. Blood samples (0.05 mL) were collected into heparinized tubes at pre-dose and at 0, 0.0833-, 0.25-, 0.5-, 1-, 2-, 4-, 6-, 8-, and 24 h post-administration. Plasma samples were obtained by centrifuging the blood for 10 min.

The plasma supernatant was then stored at −80°C and thawed at room temperature for subsequent analysis. For preparation, 10 μL of plasma was mixed with 10 μL working solution, followed by protein precipitation with 50 μL acetonitrile containing propranolol hydrochloride internal standard (50 ng/mL). After vortexing (1 min) and centrifugation (18,800× *g*, 10 min, 4°C), the supernatant was collected, diluted two-fold with 50% acetonitrile/water (v/v), and mixed. MGD-C9 plasma concentrations were quantified by LC-MS/MS using Analyst 1.5.1 (Applied Biosystem, Shanghai, China).

Automated peak integration was performed for atlas samples. The peak area ratio (sample/internal standard) was used for concentration regression analysis, applying linear regression with 1/X^2^ weighting. Pharmacokinetic parameters (AUC, t_1/2_, MRT) across administration routes, and Cmax/Tmax after oral dosing, were calculated via non-compartmental analysis using WinNonlin v6.3 (Pharsight, Lawrenceville, GA, USA).

Experimental data were presented as “Mean ± SD” (*n* = 3). The bioavailability (*F*) was calculated using the following equation:

$$F=\left[\left(AUC_{\text{extra}-\text{venous}}/AUC_{\text{iv}}\right)\times \left({\text{dose}}_{\text{iv}}/{\text{dose}}_{\text{extra}-\text{venous route}}\right)\right]\times 100\%$$

### 2.6 Statistical Analysis and Reproducibility

All *in vitro* experiments were performed in triplicate. Data are presented as mean ± Standard Deviation (SD) or Standard Error of the Mean (SEM). Differences between variables were assessed using a two-tailed Student’s *t*-test or one-way analysis of variance (ANOVA), as appropriate. Statistical significance was defined as *p* < 0.05. Analyses used SPSS 13.0 (IBM Corp., Armonk, NY, USA) or GraphPad Prism 8.0.

## 3 Results

### 3.1 Molecular Docking and Rational Design of Novel CRBN Ligands

**Figure 2:**
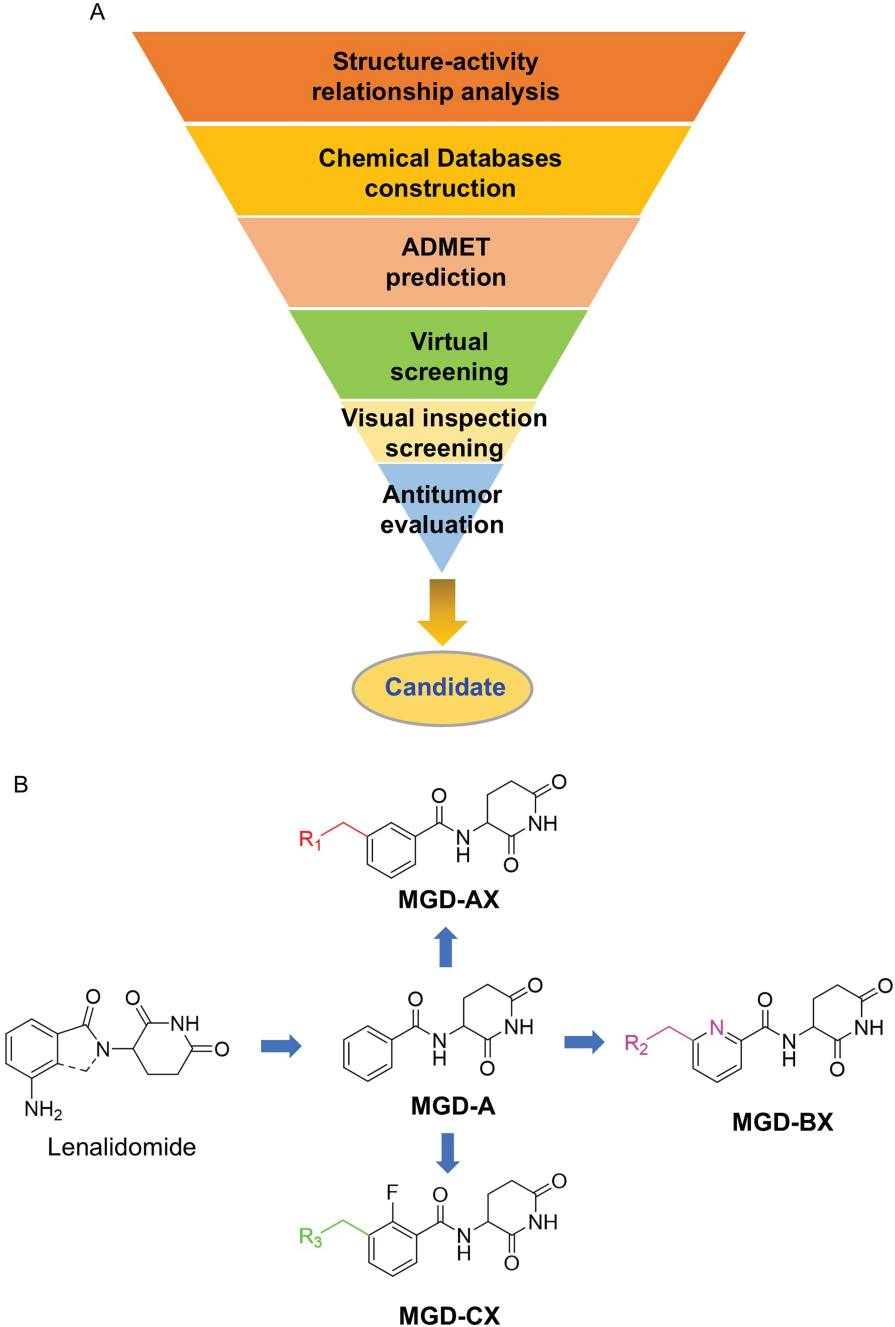
Rational design of novel CRBN ligands. (**A**) The workflow of this study; (**B**) Compounds modification based on lenalidomide

Given that nearly all CRBN ligands originate from thalidomide, we employed a rational, precise, and effective strategy to identify novel and potent CRBN ligands. As depicted in Fig. 2A, a molecular library was constructed through scaffold hopping based on thalidomide. Subsequently, ADMET (Absorption, Distribution, Metabolism, Excretion, and Toxicity) screening, virtual screening, and visual inspection screening were performed step-by-step to screen the molecular library for hits. Further optimization and antitumor evaluation were integrated for candidates.

Although the rigid isoindole ring of lenalidomide confers good physicochemical stability of lenalidomide, it also reduces the likelihood of interaction between lenalidomide and amino acid residues near the CRBN binding pocket [20]. By analyzing the binding modes after molecular docking, the molecule (MGD-A) with a novel skeleton, which was designed as a simple deletion of the methylene in the isoindole cores of lenalidomide, was selected. This modification could increase the molecule’s flexibility (Fig. 2B). Subsequently, various aliphatic nitrogen heterocyclic ring substituents (R_1_), such as pyrrole, piperidine, piperazine, morpholine, thiomorpholine groups, were introduced at the 5-position of the benzene ring to obtain a series of MGD-AX compounds. These substituents could form the salt-bridge interactions with CRBN^Glu377^, enhancing the binding ability. To verify this, the methylene connecting the R_1_ group in several compounds was replaced with the carbonyl group. The electron-absorbing capacity of the carbonyl group reduces the alkalinity of the nitrogen atom on the R_1_ group, thus impeding the formation of the salt bridge. Furthermore, we hypothesized that intramolecular hydrogen bonds could assist CRBN ligands in forming specific three-dimensional conformations to enhance binding affinity and pharmacological activity. Building on the structure of MGD-AX, the carbon atom at the 4-position of the benzene ring was replaced by a nitrogen atom to obtain a series of MGD-BX compounds, which could form an intramolecular hydrogen bond with the amide on the ortho-substituent group. Similarly, the introduction of fluorine substituents at the 4-position of the benzene ring led to a series of MGD-CX compounds, which could also form intramolecular hydrogen bonds.

### 3.2 Synthesis and Antiproliferation Evaluation of Compounds across Multiple Hematological Cancer Cell Lines

The synthetic routes for the desired CRBN ligands were presented in Fig. S1A–C. Cell-based phenotypic screening of the library across multiple cell lines would allow for the triage and prioritization of hits with broad-spectrum anticancer effects. Consequently, we conducted full dose-response cell viability assays on the library compounds, employing a panel of hematological cell lines including NCI-H929 (MM), MV-4-11(AML), and Ocl-Ly3 (DLBCL) cells. As shown in Table 1, the prototypical IMiDs, such as thalidomide, lenalidomide, and pomalidomide, demonstrated relatively low potency, with IC_50_ values in the micromolar range in NCI-H929 cells, and their IC_50_ potency was in the double-digit micromolar range in MV-4-11 and Ocl-Ly3 cells. Compounds MGD-A1 to MGD-A9 demonstrated higher potency with submicromolar to micromolar IC_50_ values. Among these compounds, MGD-A7 exhibited the strongest degradation activity, with IC_50_ values of 0.67, 0.59, and 2.32 µM in NCI-H929, MV-4-11, and Ocl-Ly3 cells, respectively. These values were approximately 5–10 times more potent than those of the prototypical IMiDs. Compared with MGD-A5, replacing the methylene connecting the p-methylpiperidine with a carbonyl group, yielding MGD-A12, led to a more than 3- to 5-fold reduction in potency. Similar effects were also shown between MGD-A5 and MGD-A13. Other compounds (MGD-A10, MGD-A11, MGD-A14, MGD-A14) with a carbonyl group connecting the R_1_ group exhibited mild activity comparable to that of the prototypical IMiDs.

**Table 1:** Antiproliferative effects of analogues MGD-AX

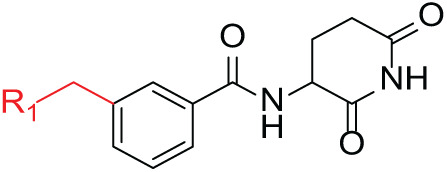				
Cpd.	R_**1**_	IC_**50**_ (**μ**M)^**a**^		
		NCI-H929	MV-4-11	Ocl-Ly3
**MGD-A1**	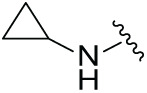	10.34 ± 0.98	7.34 ± 0.70	>33.00
**MGD-A2**	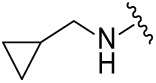	12.74 ± 1.21	8.30 ± 0.79	21.32 ± 2.03
**MGD-A3**	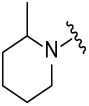	5.39 ± 0.51	6.03 ± 0.58	7.27 ± 0.69
**MGD-A4**	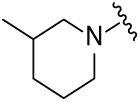	4.56 ± 0.44	3.11 ± 0.30	6.33 ± 0.60
**MGD-A5**	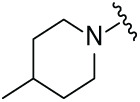	2.88 ± 0.28	2.03 ± 0.19	4.03 ± 0.38
**MGD-A6**	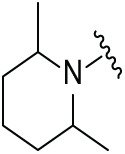	3.02 ± 0.38	3.44 ± 0.33	5.90 ± 0.56
**MGD-A7**	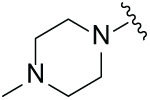	0.67 ±0.10	0.59 ± 0.07	2.32 ± 0.22
**MGD-A8**	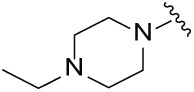	0.90 ± 0.13	0.80 ± 0.10	4.12 ± 0.39
**MGD-A9**	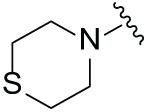	2.11 ± 0.20	1.90 ± 0.18	5.44 ± 0.52
**MGD-A10**	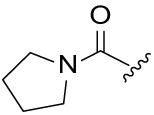	14.23 ± 1.36	8.34 ± 0.79	>33.00
**MGD-A11**	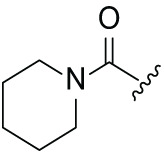	8.33 ± 0.80	5.47 ± 0.52	22.97 ± 2.19
**MGD-A12**	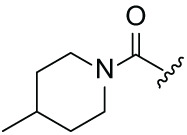	11.93 ± 1.14	14.31 ± 1.36	15.35 ± 1.46
**MGD-A13**	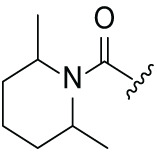	10.35 ± 0.98	9.26 ± 0.88	17.25 ± 1.64
**MGD-A14**	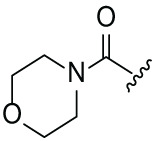	7.43 ± 0.71	5.10 ± 0.49	10.63 ± 1.01
**MGD-A15**	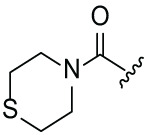	6.90 ± 0.66	4.45 ± 0.42	9.43 ± 0.90
Thalidomide	——	9.86 ± 0.94	26.48 ± 2.52	>33.00
Lenalidomide	——	7.36 ± 0.70	17.85 ± 1.70	27.58 ± 2.63
Pomalidomide	——	2.18 ± 0.21	16.15 ± 1.54	18.26 ± 1.74

Note: Cpd., compounds; ^a^Data are presented as mean ± SEM of three independent measurements.

Next, the effect of intramolecular hydrogen bonds on pharmacological activity was examined. Among compounds MGD-B1 to MGD-B10, MGD-B3 exhibited the strongest antiproliferative activity, with IC_50_ values of 0.92, 0.72, and 2.03 µM in NCI-H929, MV-4-11, and Ocl-Ly3 cells, respectively. Compared with MGD-A7, MGD-B3, in which the carbon atom of the 4-position in the benzene ring was replaced by a nitrogen atom, exhibited merely comparable antiproliferative efficacy. Similar effects were also observed between MGD-A9 and MGD-B5 (Table 2). Among compounds MGD-CX analogues, the piperidone derivative (MGD-C9) displayed the strongest antiproliferative activity, with IC_50_ values of 0.26, 0.21, and 0.81 µM in NCI-H929, MV-4-11, and Ocl-Ly3 cells, respectively, which were approximately 10–20 times more potent than those of the prototypical IMiDs (Table 3). Additionally, MGD-C9 exhibited comparable efficacy compared with avadomide in NCI-H929 (IC_50_ = 0.20 µM), and showed approximately 4-fold higher antiproliferative activity than avadomide in MV-4-11 cells (IC_50_ = 0.85 µM) and approximately 20 times stronger in Ocl-Ly3 cells (IC_50_ = 15.54 µM), respectively (Fig. S1D). Moreover, compared with MGD-B9, MGD-C7 showed higher antiproliferative efficacy. Similar trends were also shown between MGD-B10 and MGD-C8. These findings suggest that fluorine substituents at the 4-position of the benzene ring have a more pronounced effect on enhancing pharmacological activity.

**Table 2:** Antiproliferative effects of analogues MGD-BX

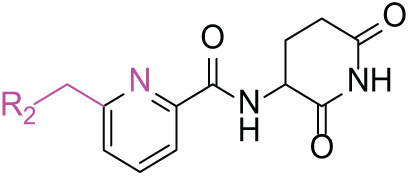				
Cpd.	R_**2**_	IC_**50**_ (**μ**M)^**a**^		
		NCI-H929	MV-4-11	Ocl-Ly3
**MGD-B1**	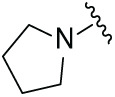	7.90 ± 0.75	14.95 ± 1.42	22.37 ± 2.13
**MGD-B2**	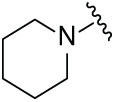	8.50 ± 0.81	6.78 ± 0.65	>33.00
**MGD-B3**	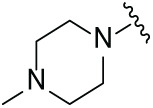	0.92 ± 0.12	0.72 ± 0.13	2.03 ± 0.39
**MGD-B4**	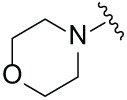	4.65 ± 0.44	3.02 ± 0.29	14.95 ± 1.42
**MGD-B5**	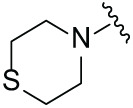	2.77 ± 0.36	1.58 ± 0.47	4.68 ± 2.44
**MGD-B6**	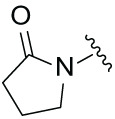	4.89 ± 0.47	3.34 ± 0.32	27.48 ± 2.62
**MGD-B7**	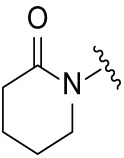	3.21 ± 0.31	5.65 ± 0.54	15.15 ± 1.44
**MGD-B8**	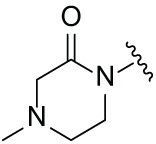	1.77 ± 0.17	1.33 ± 0.13	8.43 ± 0.80
**MGD-B9**	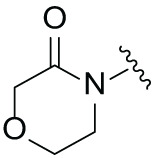	2.34 ± 0.22	1.93 ± 0.18	9.35 ± 0.89
**MGD-B10**	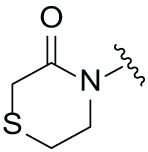	4.32 ± 0.41	5.54 ± 0.53	13.24 ± 1.26
Thalidomide	——	9.86 ± 0.94	26.4 ± 2.52	>33.00
Lenalidomide	——	7.36 ± 0.70	17.8 ± 1.70	27.58 ± 2.63
Pomalidomide	——	2.18 ± 0.21	16.15 ± 1.54	18.26 ± 1.74

Note: Cpd., compounds; ^a^Data are presented as mean ± SEM of three independent measurements.

**Table 3:** Antiproliferative effects of analogues MGD-CX

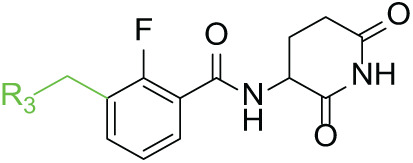				
Cpd.	R_**3**_	IC_**50**_ (**μ**M)^**a**^		
		NCI-H929	MV-4-11	Ocl-Ly3
**MGD-C1**	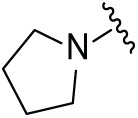	6.91 ± 0.66	7.36 ± 0.70	12.64 ± 1.20
**MGD-C2**	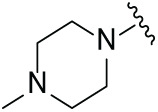	0.77 ± 0.04	0.54 ± 0.05	1.64 ± 0.37
**MGD-C3**	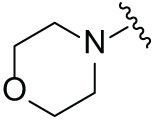	1.98 ± 0.19	0.93 ± 0.048	2.39 ± 0.23
**MGD-C4**	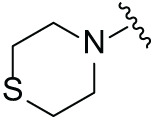	1.79 ± 0.27	2.43 ± 0.33	5.01 ± 0.48
**MGD-C5**	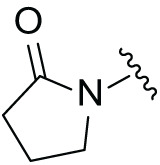	2.04 ± 0.19	2.22 ± 0.21	5.05 ± 0.48
**MGD-C6**	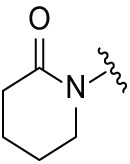	1.33 ± 0.13	1.63 ± 0.16	5.95 ± 0.57
**MGD-C7**	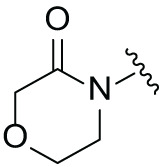	0.93 ± 0.15	0.68 ± 0.07	2.11 ± 0.20
**MGD-C8**	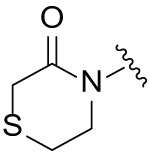	1.23 ± 0.12	1.73 ± 0.17	3.28 ± 0.31
**MGD-C9**	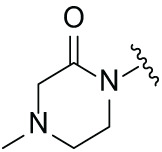	0.26 ± 0.03	0.21 ± 0.02	0.81 ± 0.09
Thalidomide	——	9.86 ± 0.94	26.48 ± 2.52	>33.00
Lenalidomide	——	7.36 ± 0.70	17.85 ± 1.70	27.58 ± 2.63
Pomalidomide	——	2.18 ± 0.21	16.15 ± 1.54	18.26 ± 1.74

Note: Cpd., compounds; ^a^Data are presented as mean ± SEM of three independent measurements.

### 3.3 The Binding Model of MGD-A7 and MGD-C9 with CRBN

**Figure 3:**
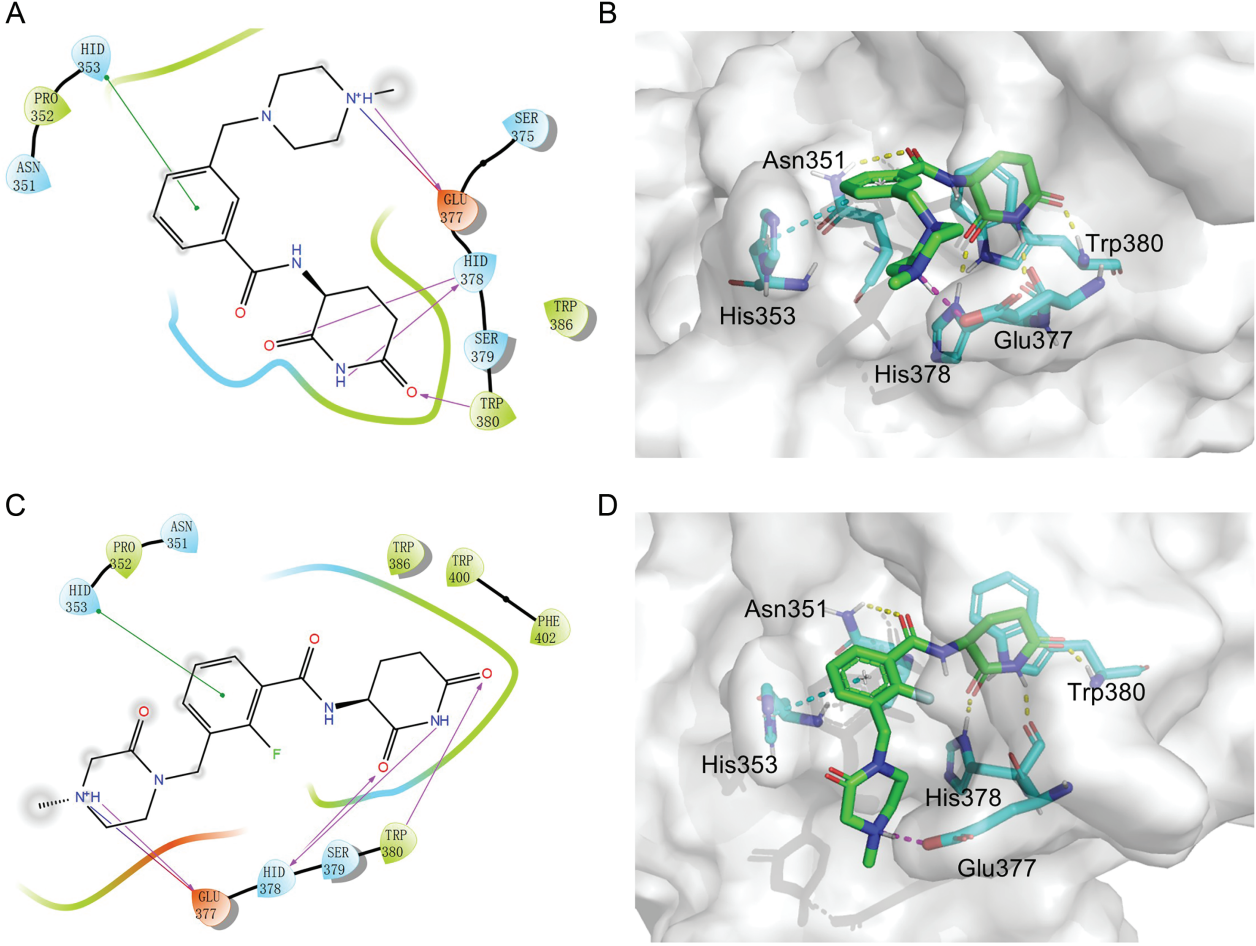
The potential interaction patterns between CRBN and MGD-A7, as well as MGD-C9. (**A**,**B**). For the CRBN-MGD-A7 pair, the two-dimensional (2D) interaction pattern (**A**) and three-dimensional (3D) interaction pattern (**B**) are depicted. In a 2D illustration, green dashes denote π-π stacking interactions and pink dashes signify hydrogen bonding interactions. (**C**,**D**). For the CRBN-MGD-C7 pair, the 2D interaction mode (**C**) and 3D interaction mode (**D**) are shown. In a 2D graph, green dashes represent π-π stacking interactions, and pink dashes represent hydrogen bonding interactions

Given the potent antitumor activity of MGD-A7 and MGD-C9 in multiple hematological cancer cells, we sought to explore the potential interaction modes between these compounds and the CRBN protein at the molecular level through molecular docking. As depicted in Fig. 3A–D, the two-dimensional and three-dimensional interaction modes of CRBN-MGD-A7/MGD-C9 complexes were visualized using the Docking scoring function. The key interactions between MGD-A7 and MGD-C9 with CRBN are generally consistent. Specifically, the glutarimide moiety of both compounds could form three hydrogen bonds with His378 and Trp380 of CRBN, similar to lenalidomide and pomalidomide, whose glutarimide moiety anchors to CRBN by the formation of hydrogen bond interactions with CRBN residues His380 and Trp382 [22,27]. Moreover, the benzene ring of MGD-A7/MGD-C9 could form π-π stacking interactions with His353 of CRBN. Additionally, the protonated nitrogen atoms of piperazine, which were exposed to the solvent region, could form salt bridge interactions with CRBN^Glu377^, which could enhance the binding ability for CRBN. Collectively, these data indicate that hydrogen bonding, π-π stacking, and salt bridge interactions play key roles in the binding of MGD-A7/MGD-C9 and CRBN protein, validating our design strategies.

### 3.4 MGD-A7 and MGD-C9 Significantly and Selectively Induced IKZF1/3 Degradation

**Figure 4:**
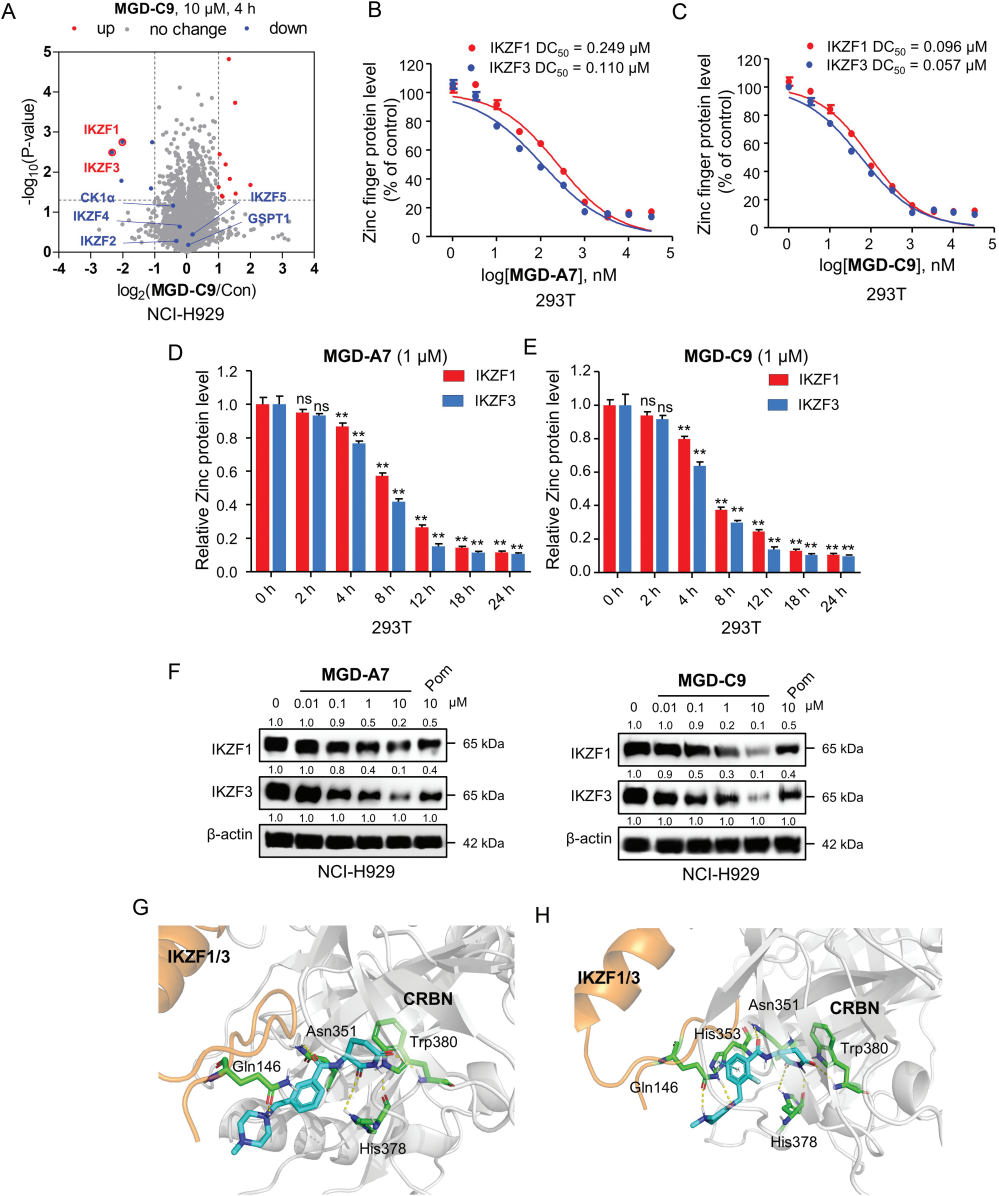
MGD-A7 and MGD-C9 significantly induced IKZF1/3 degradation. (**A**) Proteomics analysis of NCI-H929 cells treated with either MGD-C9 (10 μM) or DMSO over a 4-h period. A volcano plot was generated to compare the effects of MGD-C9 treatment with the vehicle control, which shows the downregulation of IKZF1 and IKZF3 under MGD-C9 treatment (protein false discovery rate < 1%, *n* = 3). Lines in the plot indicate significant thresholds: [log_2_(fold change)] > 1. (**B**,**C**) Engineered HEK293T (293T) cells were used to assess the levels of IKZF1 and IKZF3 exposed to increasing doses of MGD-A7 (**B**) and MGD-C9 (**C**) over a 24-h period. The IKZF1- and IKZF3-HiBiT assays were employed to measure the respective protein levels. (**D**,**E**) 293T cells stably expressing IKZF1- and IKZF3-HiBiT were used to assess the levels of IKZF1 and IKZF3 exposed to increasing treatment time of MGD-A7 (1 µM, D) and MGD-C9 (1 µM, E), respectively. (**F**) The degradation effects of MGD-A7 (left) and MGD-C9 (right) on IKZF1 and IKZF3 in NCI-H929 cells. Cells were treated with MGD-A7 and MGD-C9 at the specified concentrations over a 24-h period, respectively. (**G**,**H**) The docking models of MGD-A7 (**G**) and MGD-C9 (**H**) to the IKZF1/3 (orange)-CRBN (grey) obtained from the Protein Data Bank (PDB: 8D7Z). “ns” indicates no significance, and “**” represents *p*-value < 0.01

Numerous neo-substrates, like IKZF1/2/3, CK1α, and GSPT1, have been demonstrated to be degraded by IMiDs or their derivatives. This offers valuable understanding of their pharmacological mechanisms in cancer therapy [6,28]. To investigate the targets of MGD-C9 without preconceived bias, we carried out quantitative proteomics in NCI-H929 cells treated with 10 μM of MGD-C9 for 4 h. As presented in Fig. 4A, the proteins IKZF1 and IKZF3 were significantly downregulated following treatment with 10 µM MGD-C9, with reductions of approximately 75.1% and 80.2%, respectively. In contrast, IKZF2 and IKZF4, IKZF5, CK1α, and GSPT1 were not notably downregulated by MGD-C9, as their absolute [log_2_(fold change)] values were less than 1. To quantitatively analyze the compounds’ degradation capacity regarding the Ikaros family neosubstrates, HEK293T cells stably expressing IKZF1-, IKZF2-, and IKZF3-HiBit were employed to evaluate the degradation potency, kinetics, and depth following the treatment of MGD-A7 and MGD-C9, respectively. In comparison with pomalidomide, treatment with MGD-A7 and MGD-C9 led to a more substantial decrease in the luminescence signals of IKZF1- and IKZF3-HiBit in a dose-dependent manner (Figs. 4B,C and S2A). Specifically, pomalidomide exhibited a DC_50_ value of 0.375 μM and a maximal degradation (D_max_) of 76.2% on IKZF1, and a DC_50_ value of 0.807 μM and D_max_ of 69.4% on IKZF3. In contrast, upon treatment with MGD-A7, IKZF1 exhibited a DC_50_ value of 0.249 μM and a D_max_ of 89.4%, and a DC_50_ value of 0.110 μM and a D_max_ of 86.4% on IKZF3. In addition, MGD-C9 exhibited a DC_50_ value of 0.096 μM and a D_max_ of 90.3% on IKZF1, and a DC_50_ value of 0.057 μM and a D_max_ of 92.1% on IKZF3. Avadomide exhibited much weaker effects with a DC_50_ value of 0.347 μM and a D_max_ of 80.4% on IKZF1, and a DC_50_ value of 0.248 μM and a D_max_ of 83.1% on IKZF3 compared with MGD-C9 (Fig. S2B). Furthermore, time-course experiments revealed that treatment with 1 μM MGD-A7 for 12 h led to substantial reductions in IKZF1 (>60%) and IKZF3 (>80%) (Fig. 4D). Notably, treatment with 1 μM MGD-C9 for 8 h resulted in even stronger reductions in IKZF1 (>60%) and IKZF3 (>70%), demonstrating markedly higher potency than pomalidomide (Figs. 4E and S2C). The effects of MGD-A7 and MGD-C9 on IKZF2 were also evaluated. Analysis of luminescence signals from IKZF2-HiBit showed that MGD-A7 and MGD-C9 had minimal inhibition (Fig. S2D,E). We further assessed the ability of our degraders on endogenous degradation. Notably, MGD-A7 and MGD-C9 effectively induced the degradation of endogenous IKZF1 and IKZF3 in both NCI-H929 and MV-4-11 cells, which was remarkably greater compared to that of pomalidomide (Figs. 4F and S2F), while having minimal degradation on IKZF2. Additionally, consistent with the quantitative proteomics, MGD-A7 and MGD-C9 with increased dosing to 10 μM showed minimal degradation effects on luminescence signals of GSPT1- and CK1α-HiBit as well as endogenous CK1α and GSPT1 in both NCI-H929 and MV-4-11 cells with 24 h treatment (Fig. S2G,H). Moreover, the docking model further indicated that MGD-A7 and MGD-C9 bind to the IMiD-binding sites of the IKZF1/3-CRBN complex by forming hydrogen bond interactions with residues His353, His378, and Trp380, Asn341 of CRBN, and Gln146 of IKZF1/3, respectively (Fig. 4G,H). However, MGD-A7 cannot form hydrogen bonds with Ans351, and the number of hydrogen bonds formed between the protonated nitrogen atom and Gln146 of IKZF1/3 is less than that of MGD-C9. This indicates that MGD-C9 can induce the formation of a more stable ternary complex compared with MGD-A7, thus resulting in higher degradation efficacy. Collectively, these results demonstrated that MGD-A7 and MGD-C9 exert high antiproliferative potency by selectively and significantly inducing degradation of IKZF1/3.

### 3.5 MGD-A7 and MGD-C9 Significantly Induce Apoptosis and G1 Cell Cycle Arrest in Hematological Cancer Cells

**Figure 5:**
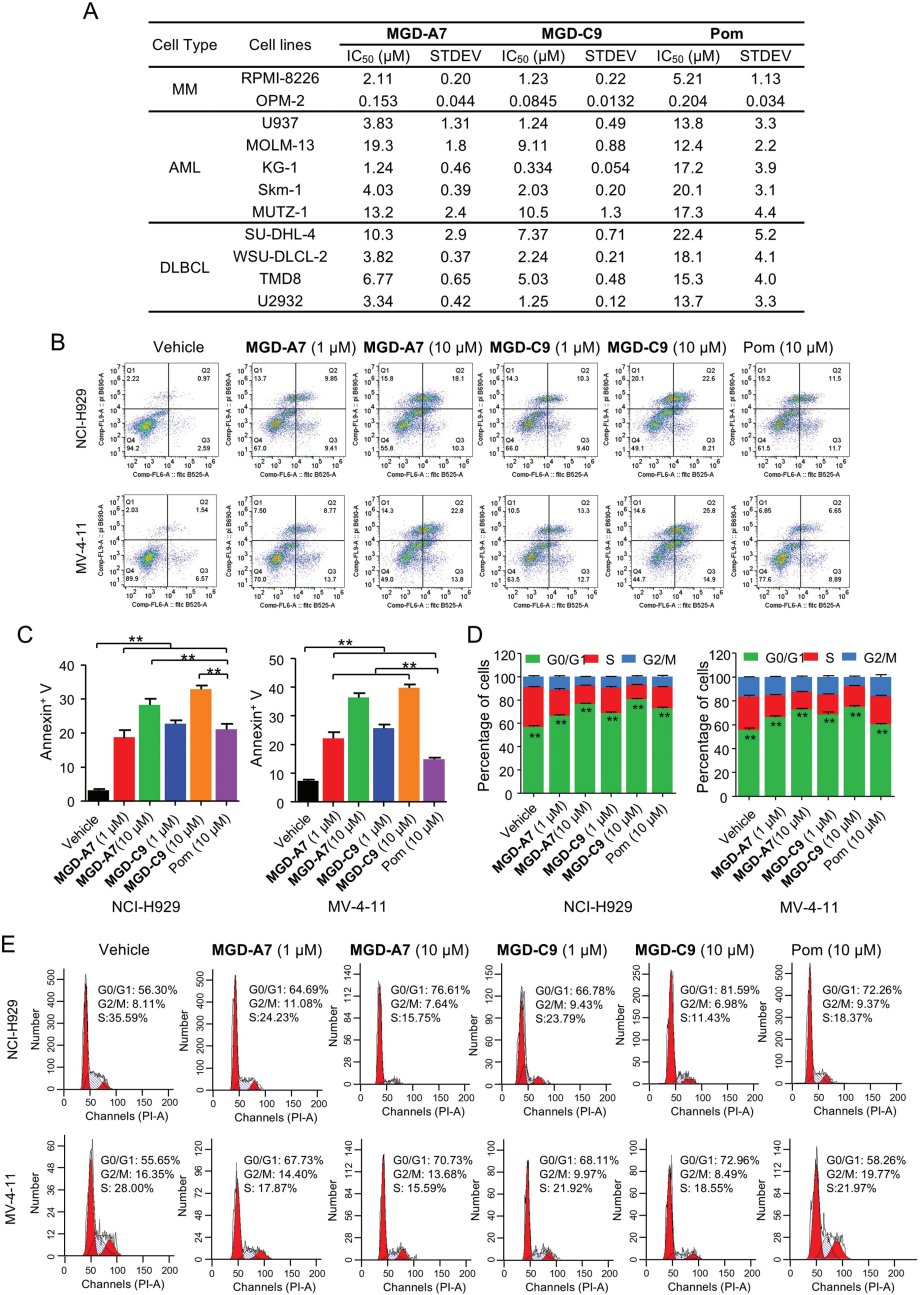
MGD-A7 and MGD-C9 significantly induce apoptosis and G1 cell cycle arrest in hematological cancer cells. (**A**) The IC_50_ values of MGD-A7, MGD-C9 and pomalidomide (Pom) in different hematological cancer cell lines over 96 h period. (**B**,**C**) Flow cytometry plot of NCI-H929 and MV-4-11 cells treated with specified doses of MGD-A7, MGD-C9 and or pomalidomide (Pom) over a 72-h period. Apoptosis was quantified by flow cytometry using Annexin V as a marker. The histograms display the relative cell percentages of apoptotic cells in each treatment group for NCI-H929 (left) and MV-4-11 cells (right). (**D**,**E**) Representative flow cytometry images illustrate the cell cycle distributions. NCI-H929 and MV-4-11 cells were treated with MGD-A7, MGD-C9, or pomalidomide (Pom) at the indicated doses over a 72-h period. The histograms show the relative cell percentages of cells in each cell cycle phase for each treatment group (up). The data are presented as the mean ± SD, with *n* = 3; Student’s *t*-test. “**” represents *p*-value < 0.01

Subsequently, we delved into the antiproliferative effect of MGD-A7 and MGD-C9 on a series of MM, AML and DLBCL cancer cell lines. As presented in Fig. 5A, in comparison with pomalidomide, MGD-A7 and MGD-C9 exhibited greater antiproliferative efficacy with IC_50_ values ranging from double-digit nanomolar to double-digit micromolar concentrations. To clarify the mechanism underlying the antiproliferative activity of MGD-A7 and MGD-C9, we investigated their capacity to trigger cell apoptosis and cell cycle transition in NCI-H929 and MV-4-11 cells. As depicted in Fig. 5B,C, in MV-4-11 cells, the apoptosis rates induced by 1 µM of MGD-A7 and MGD-C9 were 22.5% and 26.0%, respectively. When the concentration was increased to 10 µM, the apoptosis rates were 36.6% and 40.7% for MGD-A7 and MGD-C9, respectively. These rates were significantly higher than the 15.5% apoptosis rate induced by 10 µM pomalidomide. In NCI-H929 cells, 1 and 10 µM MGD-A7 and MGD-C9 also significantly induced apoptosis, with much higher rates than pomalidomide. Additionally, MGD-A7 and MGD-C9 induced cell cycle arrest at the G0/G1 phase in NCI-H929 and MV-4-11 cells in a concentration-dependent manner. Moreover, the activity of MGD-A7 and MGD-C9 was much higher than that of pomalidomide at 10 µM (Fig. 5D,E). In short, compared with pomalidomide, MGD-A7 and MGD-C9 were more efficient in inducing apoptosis and G1 cell cycle arrest in cancer cells, which is consistent with their antiproliferative activity.

### 3.6 MGD-A7 and MGD-C9 Induced Cytotoxic and Degradation Effects That Were CRBN-Dependent

**Figure 6:**
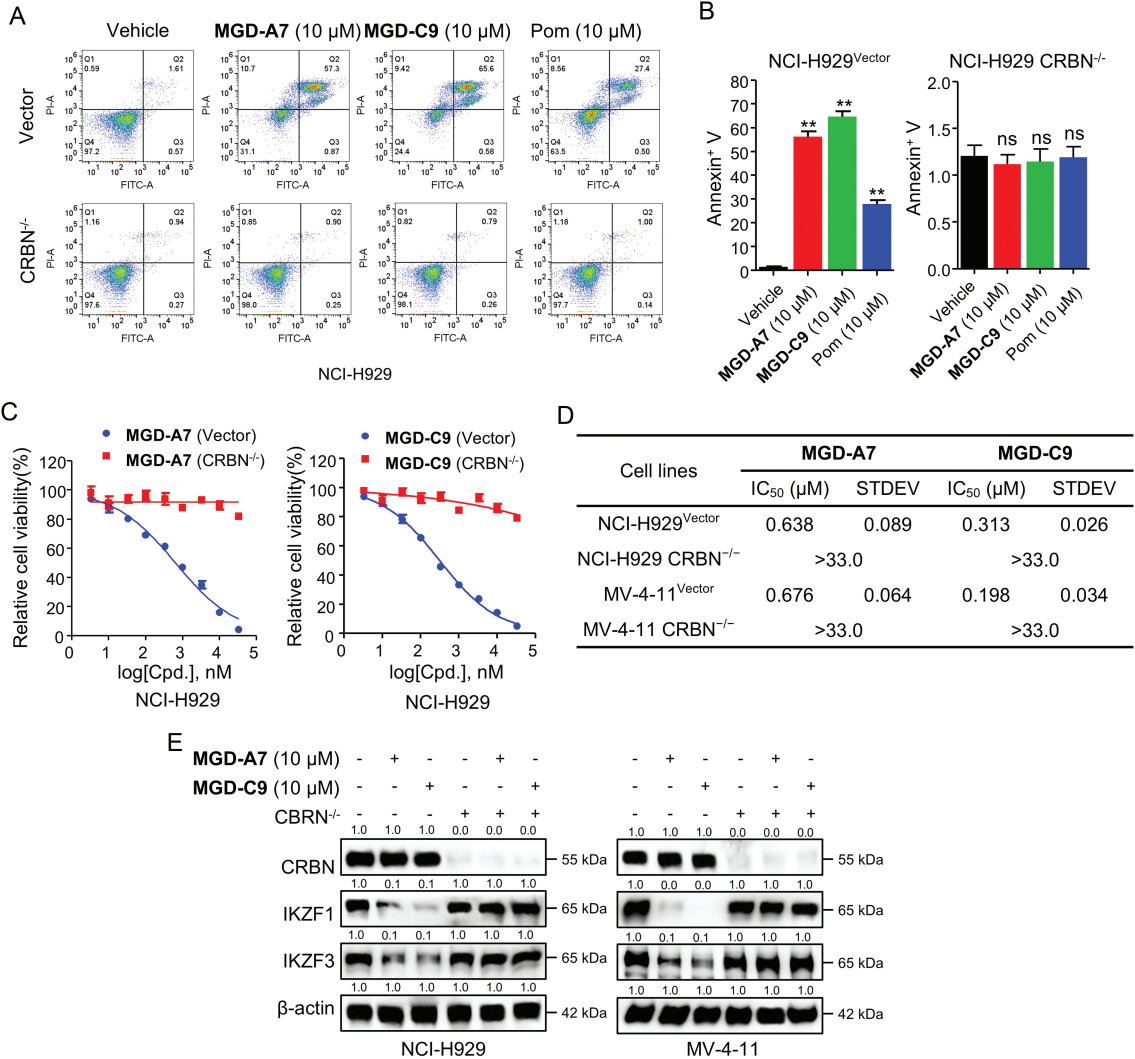
The cytotoxic and degradation effects induced by MGD-A7 and MGD-C9 were CRBN-dependent. (**A**,**B**) The representative flow cytometry plot of CRBN^–/–^ and control vector NCI-H929 cells treated with specified doses of MGD-A7 and MGD-C9 or pomalidomide (Pom) over a 72-h period. Apoptosis rates were then measured with Annexin V serving as a marker (**B**). (**C**,**D**) The cell viability (**C**) and IC_50_ values (**D**) of CRBN^–/–^ and control vector NCI-H929 cells treated with MGD-A7 or MGD-C9 over a 96-h period. (**E**) Western blot analysis in CRBN^−/−^ or control vector NCI-H929 and MV-4-11 cells. The cells were treated with MGD-A7 and MGD-C9 over a 24-h period. The data are presented as the mean ± SD, with *n* = 3. Student’s *t*-test, where “ns” indicates no significance, and “**” represents *p*-value < 0.01 compared with vehicle

After identifying MGD-A7 and MGD-C9 as potent antiproliferative and degrading agents, we explored whether their effects are CRBN-dependent. To explore this, CRBN knockout (CRBN^−/−^) NCI-H929 and MV-4-11 cells were utilized to identify whether the observed effects were mediated by CRBN. In control vector NCI-H929 cells, both MGD-A7, MGD-C9, and pomalidomide induced significant apoptosis. In contrast, neither compound elicited apoptosis in CRBN^−/−^ NCI-H929 cells (Fig. 6A,B). In addition, unlike the submicromolar antiproliferative activity observed in control vector NCI-H929 and MV-4-11 cells, the antiproliferative activity of MGD-A7 and MGD-C9 was markedly diminished in corresponding CRBN^−/−^ cells (Figs. 6C,D and S3A,B). Furthermore, rescue of IKZF1/3 degradation was observed in CRBN^−/−^ NCI-H929 and MV-4-11 cells (Fig. 6E). Collectively, these data demonstrate that MGD-A7 and MGD-C9 induce cytotoxic and degradation effects on IKZF1/3 through a CRBN-dependent manner.

### 3.7 MGD-C9 Exhibited Favorable Pharmacokinetic Properties In Vivo

The pharmacokinetic (PK) properties of MGD-C9 were next evaluated. As shown in Table 4, following a single intravenous (i.v.) administration and oral (p.o.) administration at doses of 2 and 50 mg/kg, respectively, we observed low clearance values of 4.39 and 1.49 L/h/kg. The distribution volume values of p.o. administration was 5.62 L/kg in mice for MGD-C9. These PK parameters indicate satisfied the exposure and limited distribution beyond the circulation compartment. Following a single oral dose of 50 mg/kg, the times to peak plasma concentrations were approximately 0.5 h, suggesting rapid absorption for MGD-C9. Furthermore, MGD-C9 exhibited a favorable oral bioavailability of 33.48%. Taken together, these favorable PK properties of MGD-C9 guarantee its efficacy *in vivo*.

**Table 4:** The pharmacokinetic parameter in mice for MGD-C9 after i.v. and p.o. administration (mean ± SD, *n* = 3)

Parameters	T_**1/2**_ (h)	T_**max**_ (h)	C_**max**_ (ng·mL^−1^)	AUC_**0-inf**_ (h·ng·mL^−1^)	MRT_**0-inf**_ (h)	Cl (L/h/kg)	Vz (L/kg)	F
**MGD-C9** (i.v.)	0.20 ± 0.07	—	3687.38 ± 712.97	1362.40 ± 190.39	0.26 ± 0.12	1.49 ± 0.22	0.42± 0.14	—
**MGD-C9** p.o.	0.89 ± 0.23	0.50 ± 0.00	5587.13 ± 221.83	11404.87 ± 553.12	1.51 ± 0.17	4.39 ± 0.22	5.62 ± 1.23	33.48%

### 3.8 MGD-C9 Displayed Remarkable Synergistic Effects When Combined with Standard-of-Care Agents

**Figure 7:**
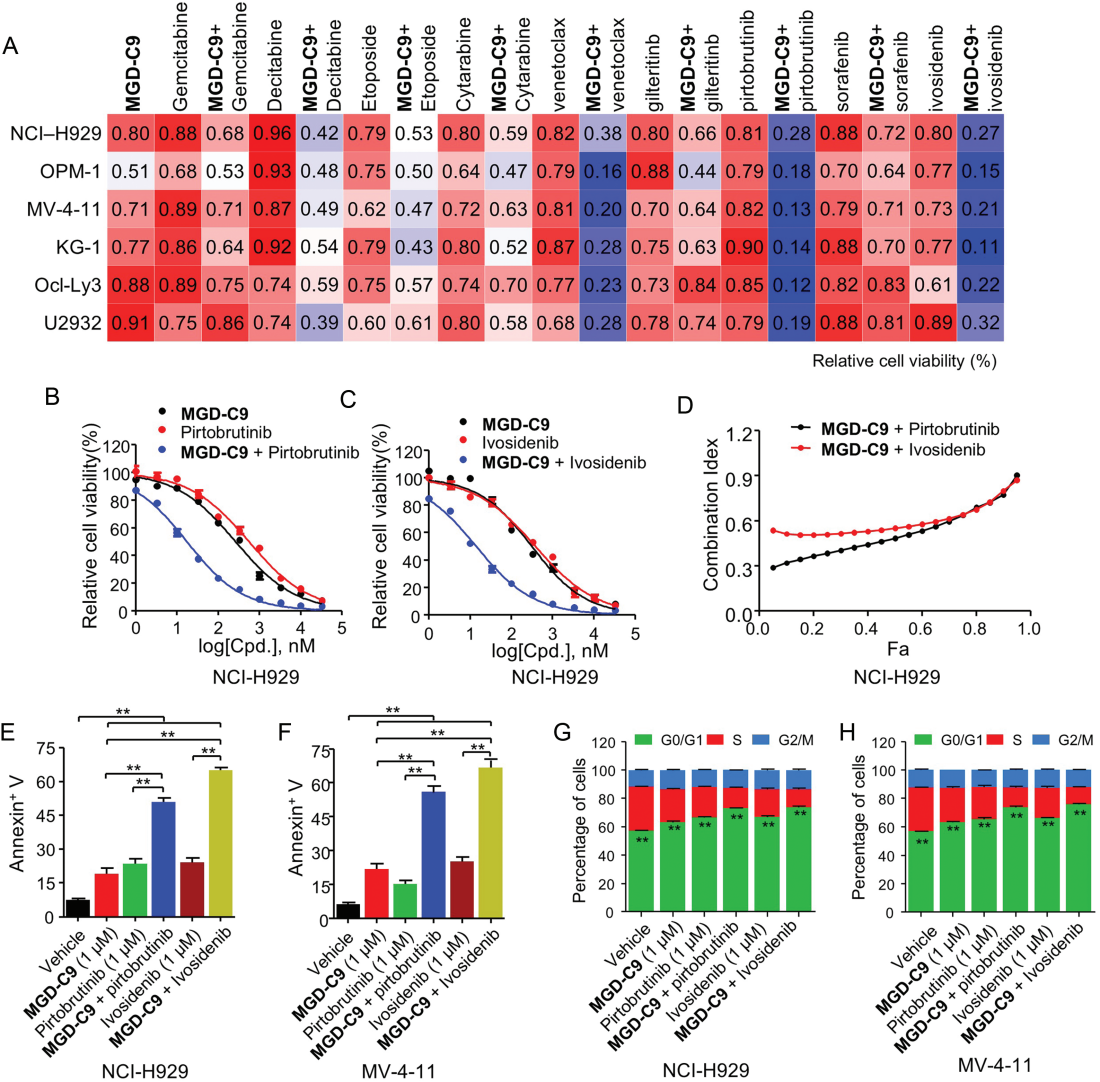
MGD-C9 exhibited remarkable synergistic effects when combined with standard-of-care agents. (**A**) The effects of MGD-C9 (1 μM) in combination with various single agents or as drug combinations were investigated in NCI-H929, OPM-1, MV-4-11, KG-1, Ocl-Ly3, and U2932 cells. The single agents included gemcitabine (0.5 μM), decitabine (1 μM), etoposide (1 μM), cytarabine (0.5 μM), venetoclax (1 μM) and gilteritinb (0.05 μM), pirtobrutinib (0.1 μM), sorafenib (1 μM) and ivosidenib (0.1 μM). (**B**,**C**) Cell viability of MGD-C9 combined with pirtobrutinib (**B**) and ivosidenib (**C**) was evaluated as single agents or in drug combinations in NCI-H929 cells over a 96-h period. (**D**) The CI values range from 0.05 to 0.95 based on the fraction affected (Fa) values, incorporating multiple doses and response points from the experiments in (**B**,**C**). The data represent the averages of three independent determinations. (**E**,**F**) Apoptosis rates in NCI-H929 (**E**) and MV-4-11 (**F**) cells treated with indicated doses of MGD-C9 with pirtobrutinib and ivosidenib as single agents or drug combinations over a 72-h period. (**G**,**H**) Relative cell percentages of cells in each cell cycle phase in NCI-H929 (**G**) and MV-4-11 (**H**) cells treated with indicated doses of MGD-C9, pirtobrutinib, and ivosidenib as single agents or drug combinations over a 72-h period. The data are presented as the mean ± SD, with *n* = 3. “**” represents *p*-value < 0.01

An increasing number research have demonstrated the clinical use of various chemotherapeutics agents, B-cell lymphoma-2 (BCL-2), Bruton’s tyrosine kinase (BTK), Isocitrate dehydrogenase 1/2 (IDH1/2) and tyrosine kinases such as Fms-like tyrosine kinase 3 (FLT3) inhibitors in clinic treatment of MM, AML and DLBCL, respectively [29–31]. Hence, we investigated whether combined treatment of inhibitors targeting these pathways could enhance the anticancer efficacy of MGD-C9. As revealed by Fig. 7A, co-treatment of chemotherapeutics such as gemcitabine, decitabine, etoposide, cytarabine, as well as targeted therapies including gilteritinib (FLT3), sorafenib (broad-spectrum tyrosine kinase inhibitor) showed minor synergetic inhibitory effects compared with monotherapy in a panel of hematological cancer cell lines. In contrast, a combination of venetoclax (BCL2 inhibitor), pirtobrutinib (BTK), and ivosidenib (IDH1 inhibitor) with MGD-C9 led to a remarkable decrease in cell viability relative to single agents, respectively. The further dose-response curves of MGD-C9 combined with pirtobrutinib, as well as ivosidenib, showed strong drug synergistic effects (Figs. 7B,C and S4A,B). The combination indexes (CI) computed using the Chou-Talalay equation for fraction affected (Fa, representing the proportion of cell viability) spanning from 0.05 to 0.95 also manifested the synergistic effects of MGD-C9 when combined with these inhibitors (combination index < 1; Figs. 7D and S4C). Furthermore, in cell apoptosis assays, combining MGD-C9 with pirtobrutinib and ivosidenib, respectively, led to a notable increase in the apoptosis rates (Fig. 7E,F) and a higher efficacy on inducing cell cycle arrest at the G0/G1 phase (Figs. 7G,H and S4D) in NCI-H929 and MV-4-11 cells compared to using either agent alone. BTK acts as a key component of the B-cell receptor signaling pathway, which is crucial for the survival and development of many B-cell malignancies [32]. Concurrently, IDH1 mutations have been observed in multiple tumors, including glioma, AML, and chondrosarcoma, and are associated with the production of an oncometabolite that promotes an abnormal cellular environment for cancer progression [33]. The observed synergistic effects of MGD-C9 with pirtobrutinib and ivosidenib, respectively, hold great promise clinical context, especially in relapsed or refractory hematological malignancies. Taken together, these findings imply that combined inhibition of BTK and IDH1 along with IMiDs can be regarded as effective treatment strategies to enhance the efficacy against hematological cancer cells and offer an alternative way to tackle acquired resistance.

## 4 Discussion

Modulating the chemical composition of CRBN binders is a critical step in optimizing protein degraders that exploit the functions of this E3 ligase. To date, FDA-approved IMiDs, including thalidomide, lenalidomide, and pomalidomide, exhibit close structural similarity. Moreover, some degraders under clinical trials like CC-220, CC-885, CC-92480 [6], as well as the recently reported NVP-DKY709, a selective IKZF2 degrader sparing IKZF1/3 [34], and dual CK1α/IKZF2 degrader DEG-35 [35], all share the isoindolinone or phthalimide scaffold, which significantly restrains the chemical space for recruiting novel neo-substrates. In contrast, MGD-A7 and MGD-C9, with a novel skeleton, were designed as a simple deletion of the methylene in the isoindole cores of lenalidomide, which could increase the molecule’s flexibility. Docking models reveal that MGD-A7 and MGD-C9 could form π-π stacking interactions with His353 of CRBN and the protonated nitrogen atoms of piperazine could form salt bridge interactions with CRBN^Glu377^. Both could enhance the binding ability for CRBN. Both compounds demonstrated superior antiproliferative efficacy vs. IMiDs. Concurrently, they achieved DC_50_ values ranging from double-digit nanomolar to submicromolar potency in degrading IKZF1/3 through a CRBN-dependent pathway. Mechanistically, MGD-A7 and MGD-C9 triggered a substantial increase in cell apoptosis and G1 cell cycle arrest. MGD-C9 exhibited favorable PK properties with low clearance values, satisfactory exposure, rapid absorption, as well as good oral bioavailability, guaranteeing its efficacy *in vivo*.

Although the efficacy of IMiDs has been continuously enhanced during drug development, common hematological toxicities, such as neutropenia, liver failure, anemia (reduced red blood cell count), constipation, diarrhea, thrombocytopenia (decreased platelet count), and potential teratogenic effects remain the major obstacles in the clinical treatment of IMiDs [11]. CC-885 demonstrated the ability to inhibit the proliferation of various AML and solid tumor cells, achieving nanomolar IC_50_ values [36]. However, its development was hindered by the discovery of uncontrolled off-target toxicity associated with the compound. To date, no clinical trials evaluating CC-885 in hematologic malignancies have been reported [12]. CFT7455, a next-generation IKZF1/3 degrader with favorable pharmacological properties in models of MM, has promoted its inclusion in a phase 1/2 study in relapsed/refractory (R/R) MM and Non-Hodgkin Lymphoma (NHL). However, 3 out of 5 MM patients developed grade 4 neutropenia side effects, requiring the clinical initiator to change the dosing regimens to improve tolerability while maintaining efficacy [37,38]. Moreover, in previous research, thalidomide and its metabolite, 5-hydroxythalidomide, were shown to induce teratogenicity via the CRBN neosubstrate PLZF [39], and another study revealed that thalidomide induces SALL4 degradation, providing a mechanistic explanation for thalidomide-induced birth defects [40]. In our global quantitative proteomics analysis of NCI-H929 cells treated with MGD-C9 (10 µM), significant reductions in IKZF1/3 levels were observed, while PLZF and SALL4 remained unaffected. Nonetheless, additional efforts are necessary to evaluate its toxicity and potential side effects during preclinical studies.

Several studies have shown that combining IMiDs with immunosuppressive steroids, proteasome inhibitors, or EZH2 inhibitors significantly improves overall survival rates in the treatment of both newly diagnosed and R/R MM [23,41,42], emphasizing the advantages of a combinational therapeutic strategy for hematological cancers. First-line therapy for patients with DLBCL primarily comprises rituximab, cyclophosphamide, doxorubicin, vincristine and prednisone (R-CHOP) [31]. Recently, a modified regimen substituting vincristine with polatuzumab vedotin (pola-R-CHP) has also been approved [43]. In addition, several targeted agents have shown promise. BTK inhibitors, which block B-cell receptor (BCR) signaling, have proven particularly effective in DLBCL. ivosidenib, an IDH1 inhibitor, induces apoptosis and has demonstrated clinical benefits in patients with various hematological malignancies [31,44]. Despite these advancements in first-line therapies, patients with relapsed or refractory DLBCL continue to necessitate novel treatment strategies. In this context, MGD-C9 displayed remarkable synergistic effects when combined with BTK inhibitor (pirtobrutinib) and IDH1 inhibitor (ivosidenib) in multiple MM and AML, and DLBCL cells. This synergistic interaction suggests that MGD-C9 could enhance the efficacy of existing treatment regimens, offering a more potent therapeutic approach. Despite these noteworthy findings, more efforts should be conducted to investigate the effects of MGD-C9 as a single agent or combined with inhibitors of BTK and IDH1, respectively, in xenograft tumor models.

## 5 Conclusion

In conclusion, we report the development of a series of novel CRBN binding moieties, envisioned as a simple deletion of the methylene in the isoindole cores of lenalidomide. Through elaborate SAR, rational drug design and chemical synthesis of the modified derivatives, cell-based antiproliferation assays and degradation profiles investigations in multiple hematological cancer cells, we identified potent candidates, MGD-A7 and MGD-C9. Both degraders demonstrated IC_50_ values spanning from submicromolar to double-digit micromolar across a diverse spectrum of MM, AML, and DLBCL cell lines. Our study underscores the significant potential of MGD-C9, serve as a highly potent CRBN ligand and a promising candidate for the treatment of hematological malignancies, which not only stands out as a highly potent CRBN ligand but also emerges as a promising candidate for the treatment of hematological malignancies. Furthermore, these results may serve as a catalyst for the advancement of PROTAC and molecular glue technologies, opening new avenues for the development of more effective anti-cancer therapies.

## Supplementary Materials





Figure S1 The synthesis steps of the compounds. **A**. Synthesis of **MGD-AX. B**. Synthesis of **MGD-BX. C**. Synthesis of **MGD-CX. D**. Antiproliferative effects of avadomide. The data are presented as the mean ± SD, with n = 3.

Figure S2 **MGD-A7** and **MGD-C9** significantly induced IKZF1/3 degradation. **A-B**. Levels of IKZF1 and IKZF3 in engineered HEK293T (293T) cells with increasing doses of pomalidomide **(A)** or avadomide **(B)** for 24 h, respectively. **C**. Levels of IKZF1 and IKZF3 in engineered 293T cells with increasing treatment time of pomalidomide (1 µM), respectively. **D**. Levels of IKZF2 in engineered HEK293T (293T) cells with increasing doses of **MGD-A7** and **MGD-C9** for 24 h, respectively. Data shown are a representative graph of three independent experiments. **E**. Levels of IKZF2 in engineered HEK293T cells with increasing treatment time of **MGD-A7** and **MGD-C9** (1 µM), respectively. **F**. The degradation effects of **MGD-A7** (left) and **MGD-C9** (right) on IKZF1 and IKZF3 in MV-4-11 cells treated for 24 h at the indicated concentration, respectively. **G**. Levels of CK1α (left) and GSPT1 (right) in engineered HEK293T (293T) cells with increasing doses of **MGD-C9** for 24 h, respectively. **H**. The degradation effects of **MGD-C9** on CK1α and GSPT1 in NCI-H929 (left) and **MV-4-11** (right) cells treated for 24 h at the indicated concentration, respectively. Data shown are a representative graph of three independent experiments; mean ± SD of triplicates. Student’s *t* test, where “ns” indicates no significance, and “**” represents *p*-value < 0.01.

Figure S3 Viability of control vector and CRBN-/- MV-4-11 cells treated with **MGD-A7 (A)** and **MGD-C9 (B)** for 96 h. Data shown are a representative graph of three independent experiments; mean ± SD of triplicates.

Figure S4 **MGD-C9** displayed significant synergistic effects with standard-of-care agents. **A-B**. Effects of **MGD-C9** with pirtobrutinib **(A)** and ivosidenib **(B)** as single agents or drug combinations in NCI-H929 cells. Viability was measured 96 h after treatment with the indicated concentrations of drugs. **C**. The CI values were calculated from Fa values of 0.05-0.95 by the Chou-Talalay equation using multiple doses and response points in **(A, B)**, and the data are averages of three independent determinations. **D**. Representative flow cytometry images illustrate the cell cycle distributions in NCI-H929 (up) and MV-4-11 (bottom) cells treated with indicated doses of **MGD-C9**, pirtobrutinib and ivosidenib as single agents or drug combinations over a 72-hour period. Data are mean ± SD, n = 3.

## Data Availability

This research’s experimental data is available upon request from the first and corresponding authors.

## Supplementary Materials

The supplementary material is available online at https://www.techscience.com/doi/10.32604/or.2025.065123/s1.
