# Supplementary material for: Discovery and Characterization of Novel IKZF1/3 Glue Degraders against Multiple Hematological Cancer Cell Lines
Source: Oncol Res. 2025 Sep 26;33(10):2981–3006. doi: 10.32604/or.2025.065123 (PMC12494099; doi:10.32604/or.2025.065123)
Supplement: Supplementary file 1 [file OncolRes-33-65123-s001.docx]

**Supplementary figures**

**
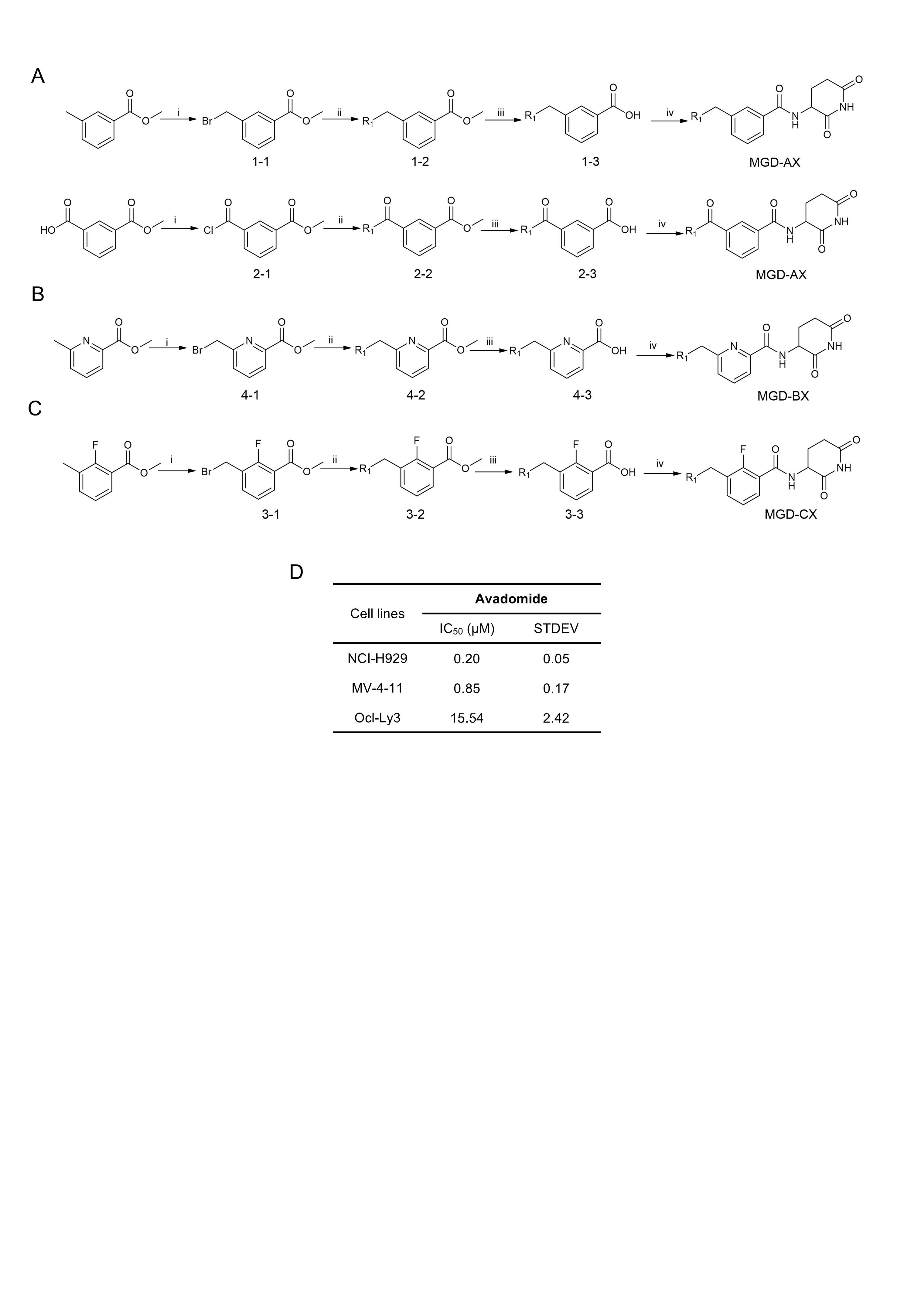
**

**FIGURE S1.** The synthesis steps of the compounds. **A**. Synthesis of **MGD-AX**. **B**. Synthesis of **MGD-BX**. **C**. Synthesis of **MGD-CX**. **D**. Antiproliferative effects of avadomide. The data are presented as the mean ± SD, with n = 3.

**
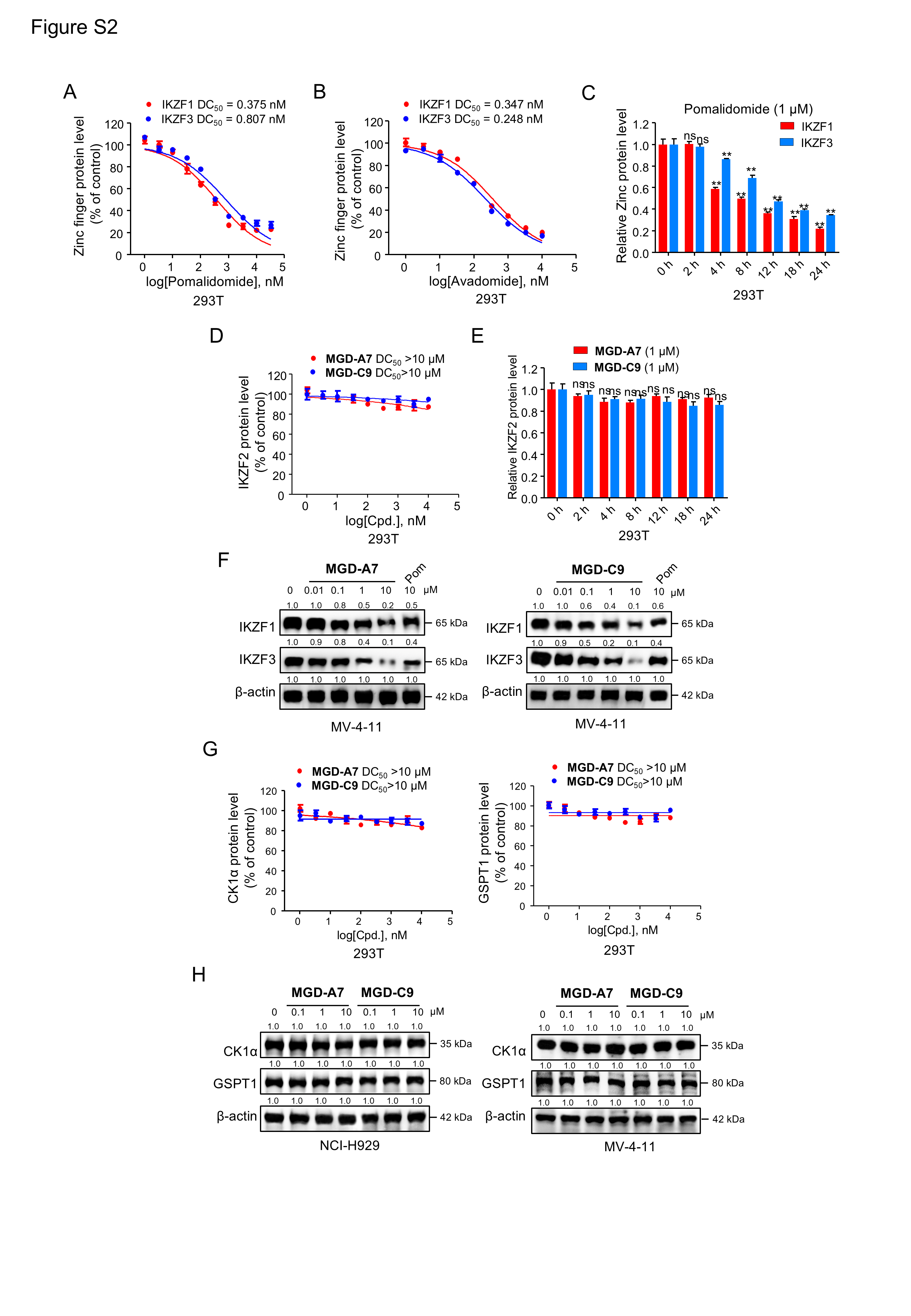
**

**FIGURE S2.**  **MGD-A7** and **MGD-C9** significantly induced IKZF1/3 degradation. **A-B**. Levels of IKZF1 and IKZF3 in engineered HEK293T (293T) cells with increasing doses of pomalidomide (**A**) or avadomide (**B**) for 24 h, respectively. **C**. Levels of IKZF1 and IKZF3 in engineered 293T cells with increasing treatment time of pomalidomide (1 µM), respectively. **D**. Levels of IKZF2 in engineered HEK293T (293T) cells with increasing doses of **MGD-A7** and **MGD-C9** for 24 h, respectively. Data shown are a representative graph of three independent experiments. **E**. Levels of IKZF2 in engineered HEK293T cells with increasing treatment time of **MGD-A7** and **MGD-C9** (1 µM), respectively. **F**. The degradation effects of **MGD-A7** (left) and **MGD-C9** (right) on IKZF1 and IKZF3 in MV-4-11 cells treated for 24 h at the indicated concentration, respectively. **G**. Levels of CK1α (left) and GSPT1 (right) in engineered HEK293T (293T) cells with increasing doses of **MGD-C9** for 24 h, respectively. **H**. The degradation effects of **MGD-C9** on CK1α and GSPT1 in NCI-H929 (left) and MV-4-11 (right) cells treated for 24 h at the indicated concentration, respectively. Data shown are a representative graph of three independent experiments; mean ± SD of triplicates. Student’s *t* test, where “ns” indicates no significance, and “**” represents *p*-value < 0.01.

**
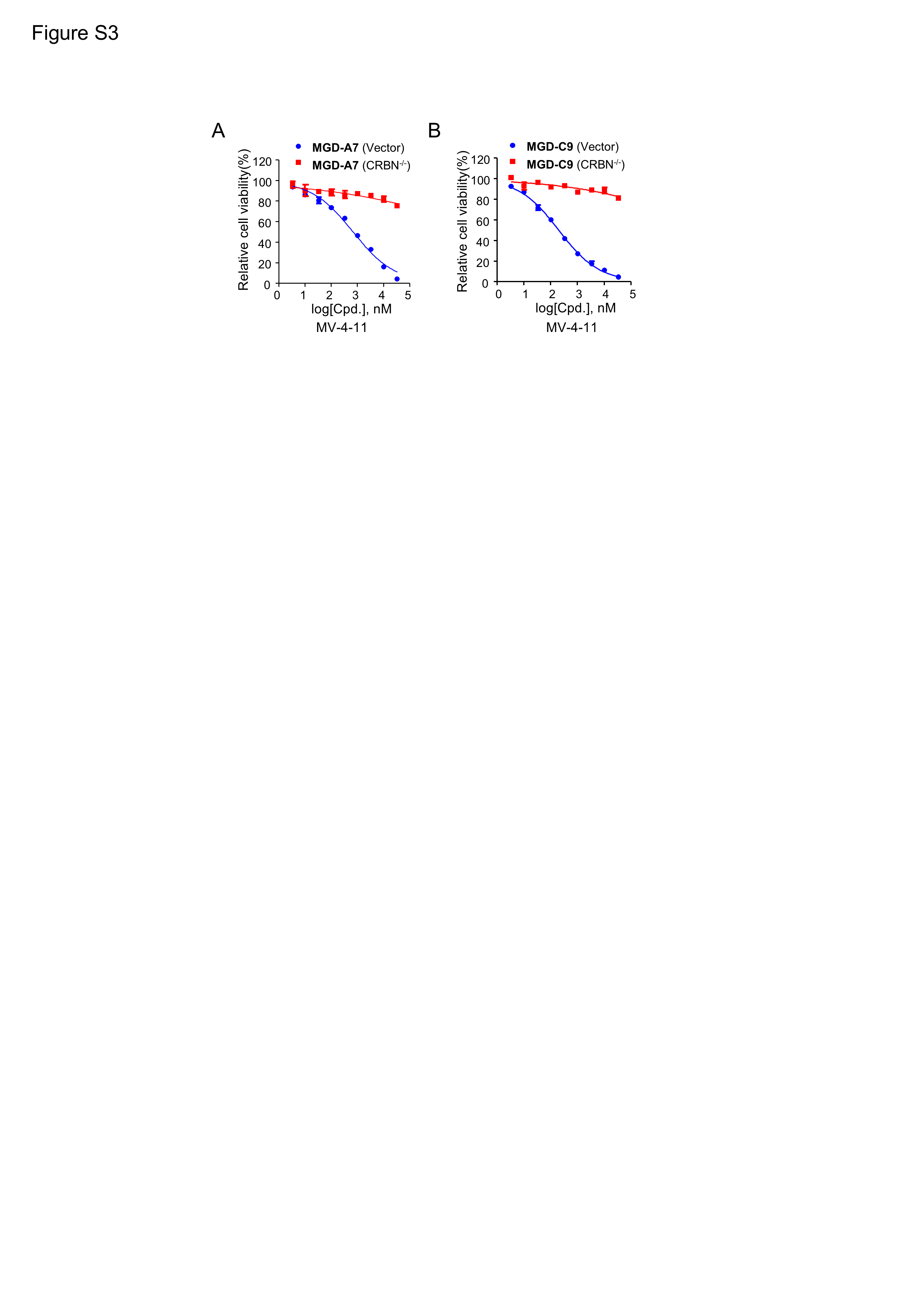
**

**FIGURE S3.** Viability of control vector and CRBN^−/−^ MV-4-11 cells treated with **MGD-A7** (**A**) and **MGD-C9** (**B**) for 96 h. Data shown are a representative graph of three independent experiments; mean ± SD of triplicates.

**
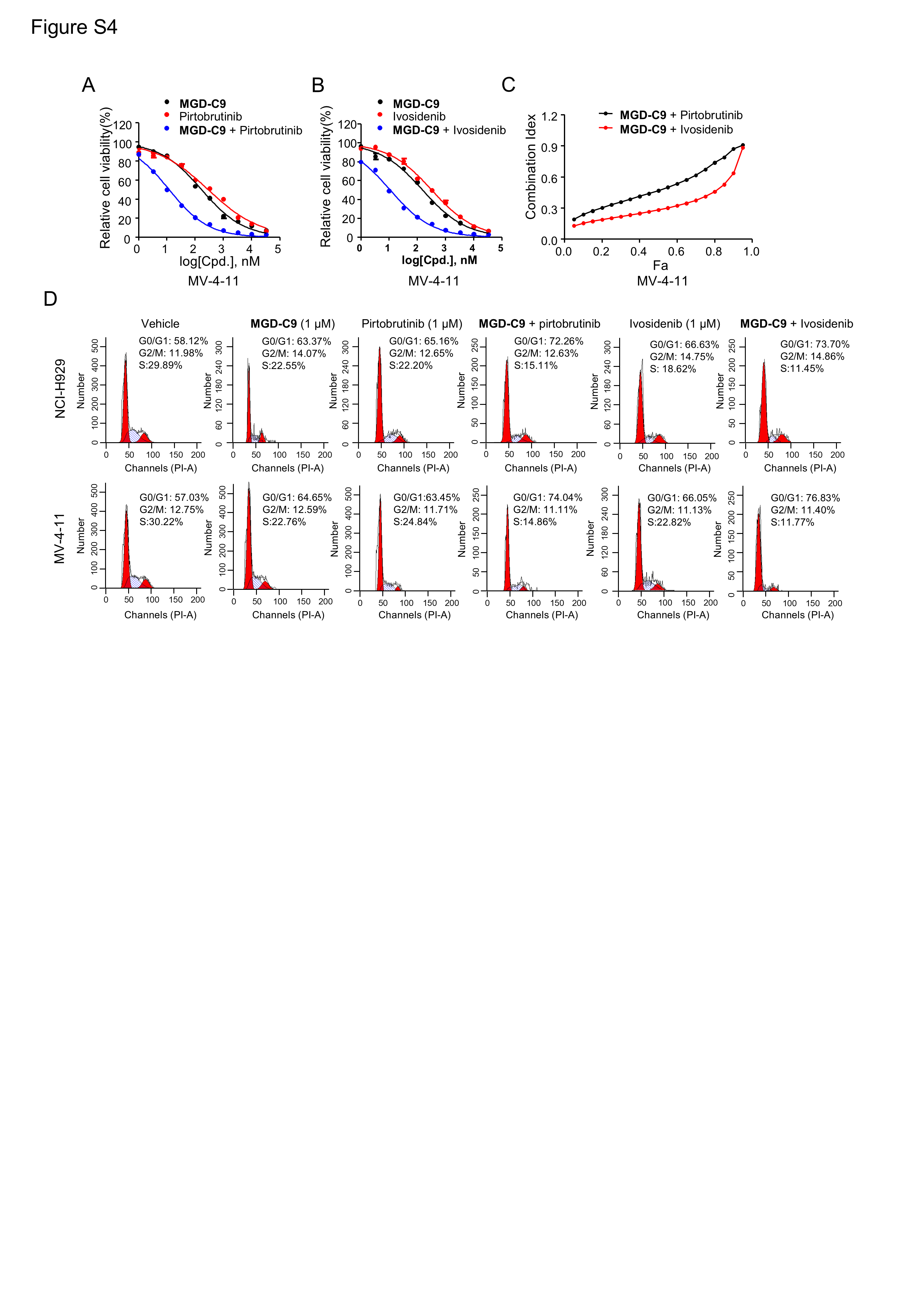
**

**FIGURE S4. MGD-C9** displayed significant synergistic effects with standard-of-care agents. **A-B**. Effects of **MGD-C9** with pirtobrutinib (**A**) and ivosidenib (**B**) as single agents or drug combinations in NCI-H929 cells. Viability was measured 96 h after treatment with the indicated concentrations of drugs. **C**. The CI values were calculated from Fa values of 0.05-0.95 by the Chou-Talalay equation using multiple doses and response points in (**A**, **B**), and the data are averages of three independent determinations. **D**. Representative flow cytometry images illustrate the cell cycle distributions in NCI-H929 (up) and MV-4-11 (bottom) cells treated with indicated doses of **MGD-C9**, pirtobrutinib and ivosidenib as single agents or drug combinations over a 72-hour period. Data are mean ± SD, n = 3.
