# Supplementary material for: Discovery and Characterization of Novel IKZF1/3 Glue Degraders against Multiple Hematological Cancer Cell Lines
Source: Oncol Res. 2025 Sep 26;33(10):2981–3006. doi: 10.32604/or.2025.065123 (PMC12494099; doi:10.32604/or.2025.065123)
Supplement: Supplementary file 2 [file OncolRes-33-65123-s002.docx]

**Supplementary** **Materials Section 2 and 3**

**Table of contents**

**1. Synthesis of compounds and compounds characterization**

**2. ^1^H NMR and ^13^C NMR of MGD-AX, MGD-BX, MGD-CX analogues.**

**1. Synthesis of compounds and compounds characterization**

**1.1** *methyl 3-(bromomethyl)cyclohexa-1,3-diene-1-carboxylate (****1-1****)*

To the solution of NBS (2.33 g, 13.08 mmol) in DCM (10 mL), methyl 3-methylcyclohexa-1,3-diene-1-carboxylate (1.81 g, 11.89 mmol) was added at 0℃. The mixture was stirred to 80 °C for 1 h, and then cooled to 0℃. After filtration, the filtrate was concentrated under reduced pressure. Then, the resulting concentrate was purified by silica gel column chromatography to obtain colorless oil liquid **1-1** (2.20 g, 74.83%).

**1.2** *methyl 3-((cyclopropylamino)methyl)cyclohexa-1,3-diene-1-carboxylate* ***(1-2)***

The reaction conditions for the synthesis of intermediates 1-2 can be divided into two categories: when the R group is a nitrogenous fatty heterocycle, potassium carbonate is selected; When the R group was a lactam ring, a strong base was used instead of sodium hydride for the reaction.

Taking the synthesis of compound **MGD-A1** as an example, **1-1** (1.20 g, 4.86 mmol) and cyclopropanamine (99.93 mg, 1.75 mmol) were dissolved in EA (15 mL). The mixture was stirred at 60°C to 1 h, then cooled to 0℃, After filtration, the filtrate was concentrated under reduced pressure. Then the crude product was purified by silica gel column chromatography to obtain colorless transparent liquid **1-2**, which can be directly used for the next reaction.

**1.3** *3-((cyclopropylamino)methyl)cyclohexa-1,3-diene-1-carboxylic acid (****1-3****)*

To a mixture of 5 mL MeOH and 5 mL H_2_O was added **1-2** (0.22 g, 0.87 mmol) followed by NaOH (69.60 mg, 1.74 mmol). The mixture was stirred at 25℃ for 1 h, and was concentrated under reduced pressure to obtain white solid **1-3**. The next reaction is carried out without purification.

**1.4** *3-((cyclopropylamino)methyl)-N-(2,6-dioxopiperidin-3-yl)benzamide* ***(MGD-A1)***

To a suspended solution **1-3** (crude product, 0.87 mmol) in DCM (10 mL), HATU (418.30 mg, 1.10 mmol) and triethylamine (238.30 μL, 1.74 mmol) were added under the ice bath, stirring for 30 min. Then 3-aminopiperidine-2,6-dione (181.10 mg, 1.41 mmol) was added and stirred at rt for overnight. The mixture (2 × 3 mL) was washed with water, the organic phase was concentrated and purified by silica gel column chromatography to obtain white solid **MGD-A1**. (0.17 g, 64.84%). ^1^H NMR (400 MHz, DMSO-*d_6_*) δ (ppm): 10.87 (s, 1H), 8.75 (dd, *J* = 8.4, 4.1 Hz, 1H), 7.81 – 7.70 (m, 2H), 7.57 – 7.38 (m, 2H), 4.79 (ddt, *J* = 13.0, 8.4, 4.9 Hz, 1H), 3.80 (s, 1H), 3.70 (s, 1H), 2.81 (ddd, *J* = 18.3, 13.4, 5.5 Hz, 1H), 2.59 – 2.50 (m, 2H), 2.24 – 2.06 (m, 1H), 1.98 (ddd, *J* = 13.1, 5.9, 2.7 Hz, 1H), 1.26 – 1.14 (m, 1H), 0.37 (dd, *J* = 6.5, 4.2 Hz, 1H), 0.32 – 0.27 (m, 1H), 0.21 (p, *J* = 4.2 Hz, 1H). ESI-MS *m/z*: 302.14 [M+H]^+^.

**1.5** *3-(((cyclopropylmethyl)amino)methyl)-N-(2,6-dioxopiperidin-3-yl)benzamide* ***(MGD-A2)***

**MGD-A2** can be obtained by following **Scheme 1**, and obtained white solid (0.17 g, 61.96%). ^1^H NMR (400 MHz, DMSO-*d_6_*) δ (ppm): 10.87 (s, 1H), 8.75 (dd, *J* = 8.4, 4.1 Hz, 1H), 7.81 – 7.70 (m, 2H), 7.57 – 7.38 (m, 2H), 4.79 (ddt, *J* = 13.0, 8.4, 4.9 Hz, 1H), 3.80 (s, 1H), 3.70 (s, 1H), 2.81 (ddd, *J* = 18.3, 13.4, 5.5 Hz, 1H), 2.59 – 2.50 (m, 2H), 2.24 – 2.06 (m, 1H), 1.98 (ddd, *J* = 13.1, 5.9, 2.7 Hz, 1H), 1.26 – 1.14 (m, 1H), 0.37 (dd, *J* = 6.5, 4.2 Hz, 1H), 0.32 – 0.27 (m, 1H), 0.21 (p, *J* = 4.2 Hz, 1H). ESI-MS *m/z*: 316.16 [M+H]^+^.

**1.6** *N-(2,6-dioxopiperidin-3-yl)-3-((2-methylpiperidin-1-yl)methyl)benzamide* ***(MGD-A3)***

**MGD-A3** can be obtained by following **Scheme 1**, and obtained white solid (0.18 g, 60.24%). ^1^H NMR (400 MHz, DMSO-*d_6_*) δ (ppm): 10.87 (s, 1H), 8.74 (d, *J* = 8.3 Hz, 1H), 7.79 (s, 1H), 7.74 (d, *J* = 7.7 Hz, 1H), 7.48 (d, *J* = 7.6 Hz, 1H), 7.42 (t, *J* = 7.6 Hz, 1H), 4.83 – 4.73 (m, 1H), 3.98 (d, *J* = 13.6 Hz, 1H), 3.17 (d, *J* = 13.6 Hz, 1H), 2.81 (ddd, *J* = 17.2, 13.4, 5.5 Hz, 1H), 2.65 – 2.54 (m, 1H), 2.33 (s, 1H), 2.18 – 2.10 (m, 1H), 2.02 – 1.93 (m, 2H), 1.68 – 1.54 (m, 2H), 1.51 – 1.32 (m, 1H), 1.30 – 1.24 (m, 2H), 1.12 (d, *J* = 6.1 Hz, 3H). ESI-MS *m/z*: 344.20 [M+H]^+^.

**1.7** *N-(2,6-dioxopiperidin-3-yl)-3-((3-methylpiperidin-1-yl)methyl)benzamide* ***(MGD-A4)***

**MGD-A4** can be obtained by following **Scheme 1**, and obtained white solid (0.17 g, 56.90%). ^1^H NMR (400 MHz, DMSO-*d_6_*) δ (ppm): 10.88 (s, 1H), 8.90 (d, *J* = 6.7 Hz, 1H), 7.51 – 7.44 (m, 0H), 7.37 (s, 1H), 7.10 (s, 1H), 6.84 (s, 1H), 4.80 (ddd, *J* = 13.0, 8.4, 5.4 Hz, 1H), 4.46 – 4.35 (m, 2H), 3.65 (s, 3H), 3.57 (s, 2H), 2.81 (ddd, *J* = 18.1, 13.3, 5.5 Hz, 1H), 2.60 – 2.51 (m, 3H), 2.41 (t, *J* = 7.7 Hz, 1H), 2.24 (t, *J* = 7.4 Hz, 2H), 2.03 – 1.92 (m, 2H), 0.84 (s, 5H). ESI-MS *m/z*: 344.20 [M+H]^+^.

**1.8** *N-(2,6-dioxopiperidin-3-yl)-3-((4-methylpiperidin-1-yl)methyl)benzamide* ***(MGD-A5)***

**MGD-A5** can be obtained by following **Scheme 1**, and obtained white solid (0.16 g, 53.55%). ^1^H NMR (600 MHz, DMSO-*d_6_*) δ (ppm): 10.86 (s, 1H), 8.76 (d, *J* = 8.3 Hz, 1H), 7.79 (d, *J* = 23.7 Hz, 2H), 7.46 (d, *J* = 24.2 Hz, 2H), 4.79 (ddd, *J* = 12.6, 8.4, 5.3 Hz, 1H), 3.49 (s, 2H), 2.81 (ddd, *J* = 17.2, 13.4, 5.5 Hz, 1H), 2.55 (dt, *J* = 17.3, 3.8 Hz, 1H), 2.50 (t, *J* = 1.9 Hz, 4H), 2.14 (qd, *J* = 13.0, 4.5 Hz, 1H), 2.01 – 1.94 (m, 2H), 1.58 (s, 2H), 1.35 (s, 1H), 1.13 (d, *J* = 41.8 Hz, 2H), 0.89 (d, *J* = 6.5 Hz, 3H). ESI-MS *m/z*: 344.20 [M+H]^+^.

**1.9** *3-((2,6-dimethylpiperidin-1-yl)methyl)-N-(2,6-dioxopiperidin-3-yl)benzamide* ***(MGD-A6)***

**MGD-A6** can be obtained by following **Scheme 1**, and obtained white solid (0.16 g, 51.45%). ^1^H NMR (400 MHz, DMSO-*d_6_*) δ (ppm): 10.87 (d, *J* = 3.8 Hz, 1H), 8.98 (dd, *J* = 19.6, 8.3 Hz, 1H), 8.12 (d, *J* = 34.7 Hz, 1H), 7.97 (d, *J* = 7.9 Hz, 1H), 7.89 – 7.72 (m, 2H), 7.57 (dt, *J* = 21.9, 7.7 Hz, 1H), 4.77 (dt, *J* = 13.4, 7.0 Hz, 1H), 4.54 (d, *J* = 2.7 Hz, 1H), 4.41 (d, *J* = 4.1 Hz, 1H), 2.81 (ddd, *J* = 16.9, 13.3, 5.4 Hz, 1H), 2.56 (d, *J* = 17.5 Hz, 1H), 2.19 (q, *J* = 13.7, 12.5 Hz, 1H), 1.99 (t, *J* = 8.1 Hz, 1H), 1.58 – 1.46 (m, 4H), 1.19 (d, *J* = 6.5 Hz, 2H). ESI-MS *m/z*: 358.21 [M+H]^+^.

**1.10** *N-(2,6-dioxopiperidin-3-yl)-3-((4-methylpiperazin-1-yl)methyl)benzamide* ***(MGD-A7)***

**MGD-A7** can be obtained by following **Scheme 1**, and obtained white solid (0.17 g, 56.73%). ^1^H NMR (400 MHz, DMSO-*d_6_*) δ (ppm): 10.86 (s, 1H), 8.75 (d, *J* = 8.4 Hz, 1H), 7.79 (d, *J* = 1.7 Hz, 1H), 7.76 (dt, *J* = 7.3, 1.7 Hz, 1H), 7.50 – 7.39 (m, 2H), 4.79 (ddd, *J* = 12.3, 8.3, 5.3 Hz, 1H), 3.49 (s, 2H), 2.81 (ddd, *J* = 17.2, 13.3, 5.5 Hz, 1H), 2.57 (dd, *J* = 4.4, 3.0 Hz, 1H), 2.36 (s, 7H), 2.15 (s, 3H), 2.10 (dd, *J* = 12.9, 4.4 Hz, 1H), 1.97 (dtd, *J* = 13.1, 5.5, 2.8 Hz, 1H). ESI-MS *m/z*: 345.19 [M+H]^+^.

**1.11** *N-(2,6-dioxopiperidin-3-yl)-3-((4-ethylpiperazin-1-yl)methyl)benzamide* ***(MGD-A8)***

**MGD-A8** can be obtained by following **Scheme 1**, and obtained white solid (0.16 g, 51.31%). ^1^H NMR (400 MHz, DMSO-*d_6_*) δ (ppm): 10.87 (s, 1H), 8.76 (d, *J* = 8.3 Hz, 1H), 7.79 (d, *J* = 1.8 Hz, 1H), 7.76 (dt, *J* = 7.3, 1.7 Hz, 1H), 7.51 – 7.39 (m, 2H), 4.79 (ddd, *J* = 12.9, 8.3, 5.3 Hz, 1H), 3.50 (s, 2H), 2.81 (ddd, *J* = 17.2, 13.3, 5.5 Hz, 1H), 2.56 (d, *J* = 3.9 Hz, 1H), 2.33 (s, 9H), 2.14 (qd, *J* = 12.9, 4.4 Hz, 1H), 1.97 (dtd, *J* = 13.1, 5.4, 2.9 Hz, 1H), 0.98 (t, *J* = 7.2 Hz, 3H). ESI-MS *m/z*: 359.21 [M+H]^+^.

**1.12** *N-(2,6-dioxopiperidin-3-yl)-3-(thiomorpholinomethyl)cyclohexa-1,3-diene-1-carboxamide* ***(MGD-A9)***

**MGD-A9** can be obtained by following **Scheme 1**, and obtained white solid (0.18 g, 57.25%). ^1^H NMR (600 MHz, DMSO-*d_6_*) δ (ppm): 10.86 (s, 1H), 8.75 (d, *J* = 8.4 Hz, 1H), 7.79 (s, 1H), 7.76 (d, *J* = 7.7 Hz, 1H), 7.48 (d, *J* = 7.5 Hz, 1H), 7.44 (t, *J* = 7.5 Hz, 1H), 4.79 (ddd, *J* = 13.0, 8.3, 5.3 Hz, 1H), 3.54 (s, 2H), 3.09 (qd, *J* = 7.2, 4.5 Hz, 1H), 2.81 (ddd, *J* = 17.2, 13.4, 5.5 Hz, 1H), 2.62 (q, *J* = 6.5, 5.8 Hz, 7H), 2.55 (dt, *J* = 17.1, 3.8 Hz, 1H), 2.14 (qd, *J* = 12.9, 4.5 Hz, 1H), 1.98 (dtd, *J* = 13.0, 5.4, 2.8 Hz, 1H). ESI-MS *m/z*: 348.14 [M+H]^+^.

**1.13** *methyl 3-(chlorocarbonyl)benzoate* ***(2-1)***

To the Methyl 3-carboxybenzoate (7.21 g, 40.02 mmol) in DCM (50 mL) was added sulfoxide chloride (7.14 g, 0.06 mol) at rt. Then the mixture was stirred at 50℃ and maintained for 10 h. The mixture was filtered, and the crude product was purified by silica gel column chromatography to obtain golden yellow transparent liquid **2-1** (11.08 g, 92.98%).

**1.14** *methyl 3-(morpholine-4-carbonyl)benzoate* ***(2-2)***

To a mixture of morpholine (0.19 g, 2.18 mmol) in EA (10 mL) was added **2-1** (0.40 g, 2.01 mmol) at 0℃ and stirred for 30 min. Then removed the ice bath device and stirred for 30 minutes. The reaction solution was concentrated under reduced pressure to obtain the crude product which was washed with water (3 × 20 mL) followed by saturated sodium bicarbonate (2 × 10 mL) and saturated sodium chloride (3 × 5 mL). The organic phase liquid was concentrated under reduced pressure to obtain colorless transparent liquid **2-2** (0.48 g, 95.80%).

**1.15** 3-(morpholine-4-carbonyl)benzoic acid ***(2-3)***

To the solution of **2-2** (0.37 g, 1.48 mmol) in the mixture of water (5 mL) and MeOH (5 mL), NaOH (0.07 g, 1.75 mmol) was added, and then was heated to 55℃ . After 6 h it was cooled to rt. The reaction solution was concentrated by reduced pressure to give a white solid. To solution of the white solid in water (6 mL), HCl (1 mol/L) was slowly added to the reaction system PH=2. After the mixture was filtered, the remaining solids were washed with ice water (3 × 5 mL) and dried to give white solids 2-3 (0.24 g, 68.10%).The next reaction is carried out without purification.

**1.16** *N-(2,6-dioxopiperidin-3-yl)-3-(morpholine-4-carbonyl)benzamide* ***(MGD-A14)***

To **2-3** (0.20 g, 0.85 mmol) in DCM (5 mL) was stirred at 0℃ for 10 min, HATU (0.39 g, 1.02 mmol) and triethylamine (0.10 g, 1.02 mmol) were added. After 1 h 3-aminopiperidine-2,6-dione (0.17 g, 1.02 mmol) was added. The reaction mixture was stirred overnight at rt. After the reaction was complete monitored by TLC which was concentrated under reduced pressure, and purified by silica gel column chromatography to obtain white solid **MGD-A14** (0.20 g, 68.13%). ^1^H NMR (600 MHz, DMSO-*d_6_*) δ (ppm): 10.87 (s, 1H), 8.89 (d, *J* = 8.4 Hz, 1H), 7.96 (dt, *J* = 7.1, 1.8 Hz, 1H), 7.93 – 7.90 (m, 1H), 7.60 – 7.55 (m, 2H), 4.80 (ddd, *J* = 12.5, 8.3, 5.3 Hz, 1H), 3.75 – 3.33 (m, 9H), 2.81 (ddd, *J* = 17.3, 13.4, 5.5 Hz, 1H), 2.59 – 2.52 (m, 1H), 2.13 (qd, *J* = 12.9, 4.5 Hz, 1H), 1.99 (dtd, *J* = 13.0, 5.4, 2.8 Hz, 1H). ESI-MS *m/z*: 346.14 [M+H]^+^.

**1.17** *N-(2,6-dioxopiperidin-3-yl)-3-(pyrrolidine-1-carbonyl)benzamide* ***(MGD-A10)***

**MGD-A10** was synthesized along the same route as **MGD-A14**, and obtained white solid (0.22 g, 65.53%). ^1^H NMR (600 MHz, DMSO-*d_6_*) δ (ppm): 10.87 (s, 1H), 8.88 (d, *J* = 8.4 Hz, 1H), 8.00 (t, *J* = 1.7 Hz, 1H), 7.96 (dt, *J* = 7.8, 1.5 Hz, 1H), 7.69 (dt, *J* = 7.7, 1.4 Hz, 1H), 7.56 (t, *J* = 7.7 Hz, 1H), 4.81 (ddd, *J* = 12.4, 8.3, 5.3 Hz, 1H), 3.49 (t, *J* = 6.9 Hz, 2H), 3.37 (t, *J* = 6.6 Hz, 2H), 3.09 (d, *J* = 7.4 Hz, 2H), 2.81 (ddd, *J* = 17.2, 13.3, 5.5 Hz, 1H), 2.59 – 2.52 (m, 1H), 2.13 (qd, *J* = 12.9, 4.5 Hz, 1H), 1.99 (dtd, *J* = 13.0, 5.4, 2.9 Hz, 1H), 1.92 – 1.85 (m, 2H), 1.82 (p, *J* = 6.3 Hz, 2H). ESI-MS *m/z*: 330.14 [M+H]^+^.

**1.18** *N-(2,6-dioxopiperidin-3-yl)-3-(piperidine-1-carbonyl)benzamide* ***(MGD-A11)***

**MGD-A11** was synthesized along the same route as **MGD-A14**, and obtained white solid (0.20 g, 57.10%). ^1^H NMR (600 MHz, DMSO-*d_6_*) δ (ppm): 10.87 (s, 1H), 8.88 (d, *J* = 8.4 Hz, 1H), 7.94 (dt, *J* = 7.2, 1.8 Hz, 1H), 7.87 (t, *J* = 1.6 Hz, 1H), 7.59 – 7.52 (m, 2H), 4.80 (ddd, *J* = 12.5, 8.3, 5.3 Hz, 1H), 3.61 (s, 2H), 3.27 (s, 2H), 2.81 (ddd, *J* = 17.3, 13.4, 5.5 Hz, 1H), 2.59 – 2.52 (m, 1H), 2.13 (qd, *J* = 12.9, 4.5 Hz, 1H), 1.99 (dtd, *J* = 12.8, 5.4, 2.9 Hz, 1H), 1.62 (td, *J* = 6.6, 3.6 Hz, 2H), 1.57 (s, 3H), 1.46 (s, 2H). ESI-MS *m/z*: 344.16 [M+H]^+^.

**1.19** *N-(2,6-dioxopiperidin-3-yl)-3-(4-methylpiperidine-1-carbonyl)benzamide* ***(MGD-A12)***

**MGD-A12** was synthesized along the same route as **MGD-A14**, and obtained white solid (0.21 g, 57.60%). ^1^H NMR (600 MHz, DMSO-*d_6_*) δ (ppm): 10.87 (s, 1H), 8.88 (d, *J* = 8.3 Hz, 1H), 7.94 (dt, *J* = 7.1, 1.9 Hz, 1H), 7.87 (t, *J* = 1.7 Hz, 1H), 7.59 – 7.52 (m, 2H), 4.80 (ddd, *J* = 12.4, 8.3, 5.3 Hz, 1H), 3.50 (s, 1H), 3.04 (s, 1H), 2.80 (ddd, *J* = 17.3, 13.4, 5.5 Hz, 1H), 2.59 – 2.52 (m, 1H), 2.13 (qd, *J* = 13.0, 4.5 Hz, 1H), 1.99 (dtd, *J* = 12.8, 5.4, 2.9 Hz, 1H), 1.76 – 1.48 (m, 3H), 1.07 (s, 2H), 0.93 (d, *J* = 6.5 Hz, 3H). ESI-MS *m/z*: 358.18 [M+H]^+^.

**1.20** *3-(2,6-dimethylpiperidine-1-carbonyl)-N-(2,6-dioxopiperidin-3-yl)benzamide* ***(MGD-A13)***

**MGD-A13** was synthesized along the same route as **MGD-A14**, and obtained white solid (0.18 g, 47.51%). ^1^H NMR (600 MHz, DMSO-*d_6_*) δ (ppm): 10.87 (s, 1H), 8.87 (d, *J* = 8.4 Hz, 1H), 7.92 (dt, *J* = 7.6, 1.6 Hz, 1H), 7.83 (t, *J* = 1.8 Hz, 1H), 7.58 – 7.49 (m, 2H), 4.80 (ddd, *J* = 12.5, 8.3, 5.3 Hz, 1H), 3.09 (q, *J* = 7.3 Hz, 3H), 2.81 (ddd, *J* = 17.2, 13.4, 5.4 Hz, 1H), 2.55 (dt, *J* = 17.5, 3.6 Hz, 1H), 2.13 (qd, *J* = 13.0, 4.5 Hz, 1H), 1.99 (dtd, *J* = 12.7, 5.4, 2.8 Hz, 1H), 1.88 – 1.79 (m, 1H), 1.62 (tdd, *J* = 13.1, 6.0, 3.9 Hz, 2H), 1.58 – 1.43 (m, 2H), 1.21 (d, *J* = 6.9 Hz, 6H). ESI-MS *m/z*: 372.19 [M+H]^+^.

**1.21** *N-(2,6-dioxopiperidin-3-yl)-3-(thiomorpholine-4-carbonyl)benzamide* ***(MGD-A15)***

**MGD-A15** was synthesized along the same route as **MGD-A14**, and obtained white solid (0.22 g, 59.68%). ^1^H NMR (600 MHz, DMSO-*d_6_*) δ (ppm): 10.88 (s, 1H), 8.88 (d, *J* = 8.4 Hz, 1H), 7.96 (dt, *J* = 6.4, 2.1 Hz, 1H), 7.61 – 7.54 (m, 2H), 4.81 (ddd, *J* = 12.5, 8.3, 5.3 Hz, 1H), 3.89 (s, 2H), 3.54 (s, 2H), 3.31 (s, 3H), 2.81 (ddd, *J* = 17.3, 13.4, 5.5 Hz, 1H), 2.73 – 2.58 (m, 3H), 2.55 (dt, *J* = 17.7, 4.0 Hz, 1H), 2.13 (qd, *J* = 13.0, 4.5 Hz, 1H), 1.99 (dtd, *J* = 13.0, 5.4, 2.9 Hz, 1H). ESI-MS *m/z*: 362.12 [M+H]^+^.

**1.22** *methyl 6-bromopicolinate (****3-1****)*

To DCM (20 mL) was Methyl 6-methylpicolinate (1.80 g ,11.89 mmol) followed by AIBN (97.70 mg,0.60 mmol) and heated up to 80 ℃ and hold until the solids were dissolved. The temperature was restored to 80 after adding NBS (2.33 g, 13.08 mmol) in batches. After 1 h the heating was stopped and the reaction liquid was gradually cooled to 0℃, a large number of white crystals were precipitate and filtered out. The crude product was concentrated and separated by silica gel column chromatography (PE : EA = 100 : 1) to obtain colorless oil liquid **3-1** (2.20 g, 74.83%).

**1.23** *methyl 6-(morpholinomethyl)picolinate* ***(3-2)***

The reaction conditions for the synthesis of intermediates **1-2** can be divided into two categories: when the R group is a nitrogenous fatty heterocycle, potassium carbonate is selected; When the R group was a lactam ring, a strong base was used instead of sodium hydride for the reaction.

Taking the synthesis of compound **MGD-B4** as an example, to morpholine (175.30 mg, 2.01 mmol) in EA (20 mL) was added **3-1** (0.73 g, 4.86 mmol) and heated to 60°C one-time. After stirring for 1 hour, the mixture was cooled to rt and filtered, and the resulting filtrate was concentrated under reduced pressure which purified through a silica gel column chromatography to get colorless transparent liquid **1-2**, which can be directly used for the next reaction.

**1.24** *6-(morpholinomethyl)picolinic acid (****3-3****)*

Dissolve **3-2** (0.87 mmol) of the colorless oily liquid obtained in the previous step in a mixed solution of 5 mL methanol and 5 mL H_2_O, add NaOH (69.60 mg, 1.74 mmol) at rt for 1 h, **3-3** water solubility is excellent, directly dry the solvent to obtain a white solid. Do not do purification directly for the next reaction.

**1.25** *N-(2,6-dioxopiperidin-3-yl)-6-(pyrrolidin-1-ylmethyl)picolinamide (****MGD-B1****)*

**MGD-B1** can be obtained by following **Scheme 2**, and obtained white solid (0.12 g, 43.60%). ^1^H-NMR (600 MHz, DMSO-*d_6_*) δ (ppm): 10.91 (s, 1H), 8.96 (d, *J =* 8.3 Hz, 1H), 8.02*-*7.90 (m, 2H), 7.66 (dd, *J =* 7.6, 1.3 Hz, 1H), 4.80 (ddd, *J =* 13.2, 8.3, 5.4 Hz, 1H), 3.82 (d, *J =* 2.5 Hz, 2H), 3.65*-*3.45 (m, 1H), 2.86*-*2.72 (m, 1H), 2.53 (s, 4H), 2.24 (qd, *J =* 13.0, 4.4 Hz, 1H), 2.08*-*1.99 (m, 1H), 1.73 (q, *J =* 3.3 Hz, 4H). ^13^C-NMR (151 MHz, DMSO) δ (ppm): 173.43, 172.56, 163.96, 149.27, 139.50 (2C), 126.45, 121.87 (2C), 54.52 (2C), 50.08, 31.50, 24.60, 23.46. ESI-HRMS *m/z*: 317.1608 [M+H]^+^.

**1.26** *N-(2,6-dioxopiperidin-3-yl)-6-(piperidin-1-ylmethyl)picolinamide (****MGD-B2****)*

**MGD-B2** can be obtained by following **Scheme 2**, and obtained white solid (0.15 g, 52.18%). ^1^H-NMR (600 MHz, DMSO-*d_6_*) δ (ppm): 10.89 (s, 1H), 8.94 (d, *J =* 8.1 Hz, 1H), 7.95 (dd, *J =* 26.8, 7.6 Hz, 2H), 7.67 (d, *J =* 7.7 Hz, 1H), 4.79 (ddd, *J =* 13.3, 8.3, 5.4 Hz, 1H), 3.65 (s, 2H), 3.30 (s, 1H), 2.81 (ddd, *J =* 17.3, 13.8, 5.5 Hz, 1H), 2.45*-*2.30 (m, 4H), 2.23 (qd, *J =* 13.1, 4.4 Hz, 1H), 2.02 (dtd, *J =* 12.9, 5.4, 2.5 Hz, 1H), 1.53 (s, 4H), 1.41 (s, 2H). ^13^C-NMR (151 MHz, DMSO) δ (ppm): 173.45, 172.70, 164.28, 158.72, 149.20, 138.64, 125.90, 120.84, 64.47, 54.57 (2C), 49.98, 31.48, 25.87 (2C), 24.43, 24.20. ESI-HRMS *m/z*:331.1767 [M+H]^+^.

**1.27** *N-(2,6-dioxopiperidin-3-yl)-6-((4-methylpiperazin-1-yl)methyl)picolinamide* ***(MGD-B3)***

**MGD-B3** can be obtained by following **Scheme 2**, and obtained white solid (0.12 g, 39.93%). ^1^H-NMR (600 MHz, DMSO-*d_6_*) δ (ppm): 10.75 (s, 1H), 8.81 (d, *J =* 8.3 Hz, 1H), 7.84 (t, *J =* 7.6 Hz, 1H), 7.79 (d, *J =* 7.6 Hz, 1H), 7.51 (d, *J =* 7.6 Hz, 1H), 4.64 (ddd, *J =* 13.1, 8.3, 5.4 Hz, 1H), 3.56 (s, 2H), 2.65 (ddd, *J =* 17.4, 13.7, 5.5 Hz, 1H), 2.37*-*2.28 (m, 4H), 2.14 (s, 4H), 2.07 (td, *J =* 13.1, 4.4 Hz, 1H), 1.87 (ddt, *J =* 13.1, 8.1, 4.0 Hz, 1H). ^13^C-NMR (151 MHz, DMSO) δ (ppm): 173.45, 172.69, 164.26, 158.16, 149.27, 138.71, 126.06, 120.97, 63.60, 54.59 (2C), 52.43 (2C), 50.00, 45.33, 31.48, 24.42. ESI-HRMS *m/z*: 346.1874 [M+H]^+^.

**1.28** *N-(2,6-dioxopiperidin-3-yl)-6-(morpholinomethyl)picolinamide* ***(MGD-B4)***

**MGD-B4** can be obtained by following **Scheme 2**, and obtained white solid (0.15 g, 51.88%). ^1^H-NMR (600 MHz, Chloroform-*d*) δ (ppm): 8.71 (s, 1H), 8.53 (s, 1H), 8.08 (dd, *J =* 7.6, 1.1 Hz, 1H), 7.85 (t, *J =* 7.7 Hz, 1H), 7.62 (d, *J =* 7.8 Hz, 1H), 4.82 (ddd, *J =* 12.5, 7.1, 5.2 Hz, 1H), 3.76 (s, 6H), 2.78 (ddd, *J =* 18.1, 13.2, 5.3 Hz, 1H), 2.57 (s, 5H), 2.10 (s, 1H), 1.74 (s, 1H). ^13^C-NMR (151 MHz, DMSO) δ (ppm): 173.45, 172.70, 164.25, 158.02, 149.31, 138.71, 126.14, 121.00, 66.63 (2C), 64.24, 53.81, 50.00 (2C), 31.48, 24.42. ESI-HRMS *m/z*: 333.1556 [M+H]^+^.

**1.29** *N-(2,6-dioxopiperidin-3-yl)-6-(thiomorpholinomethyl)picolinamide (***MGD-B5***)*

**MGD-B5** can be obtained by following **Scheme 2**, and obtained white solid (0.15 g, 49.48%). ^1^H-NMR (600 MHz, DMSO-*d_6_*) δ (ppm): 10.90 (s, 1H), 9.00*-*8.89 (m, 1H), 7.97 (dd, *J =* 25.2, 7.7 Hz, 2H), 7.68 (d, *J =* 7.7 Hz, 1H), 4.79 (ddd, *J =* 13.2, 8.3, 5.4 Hz, 1H), 3.73 (s, 2H), 3.17 (d, *J =* 5.1 Hz, 1H), 2.81 (ddd, *J =* 17.2, 13.8, 5.5 Hz, 1H), 2.76*-*2.68 (m, 4H), 2.65 (s, 4H), 2.24 (q, *J =* 13.1, 12.6 Hz, 1H), 2.08*-*1.98 (m, 1H). ^13^C-NMR (151 MHz, DMSO) δ (ppm): 173.45, 172.70, 164.25, 158.37, 149.25, 138.69, 125.89, 120.93, 64.42, 55.15, 50.00, 49.07, 31.48, 27.62 (2C), 24.43. ESI-HRMS *m/z*: 349.1329 [M+H]^+^.

**1.30** *N-(2,6-dioxopiperidin-3-yl)-6-((2-oxopyrrolidin-1-yl)methyl)picolinamide (****MGD-B6****)*

**MGD-B6** can be obtained by following **Scheme 2**, and obtained white solid (0.13 g, 45.23%). ^1^H-NMR (600 MHz, DMSO-*d_6_*) δ (ppm): 10.93 (s, 1H), 8.90 (d, *J =* 8.0 Hz, 1H), 8.00 (t, *J =* 7.7 Hz, 1H), 7.96 (d, *J =* 7.6 Hz, 1H), 7.46 (d, *J =* 7.6 Hz, 1H), 4.79 (ddd, *J =* 13.0, 8.0, 5.3 Hz, 1H), 4.62*-*4.53 (m, 2H), 3.42 (t, *J =* 7.1 Hz, 2H), 3.21 (t, *J =* 6.9 Hz, 1H), 2.82 (ddd, *J =* 17.3, 13.7, 5.6 Hz, 1H), 2.35 (t, *J =* 8.1 Hz, 2H), 2.20 (qd, *J =* 12.9, 4.5 Hz, 1H), 2.12*-*2.05 (m, 2H), 2.02 (q, *J =* 7.5 Hz, 2H), 1.96 (dt, *J =* 15.2, 7.7 Hz, 1H). ^13^C-NMR (151 MHz, DMSO) δ (ppm): 174.99, 173.40, 172.70, 164.11, 156.69, 149.46, 139.20, 124.62, 121.06, 50.12, 47.70, 47.42, 40.40, 40.27, 40.13, 39.99, 39.85, 39.71, 39.57, 31.45, 30.57, 24.42, 18.09. ESI-HRMS *m/z*: 331.1401 [M+H]^+^.

**1.31** *N-(2,6-dioxopiperidin-3-yl)-6-((2-oxopiperidin-1-yl)methyl)picolinamide (****MGD-B7****)*

**MGD-B7** can be obtained by following **Scheme 2**, and obtained white solid (0.08 g, 26.70%). ^1^H-NMR (600 MHz, DMSO-*d_6_*) δ (ppm): 10.94 (s, 1H), 8.90 (d, *J =* 8.0 Hz, 1H), 7.99 (t, *J =* 7.7 Hz, 1H), 7.95 (d, *J =* 7.1 Hz, 1H), 7.45*-*7.40 (m, 1H), 4.80 (ddd, *J =* 13.0, 8.0, 5.4 Hz, 1H), 4.73*-*4.61 (m, 2H), 3.37 (d, *J =* 5.8 Hz, 2H), 2.82 (ddd, *J =* 17.3, 13.6, 5.6 Hz, 1H), 2.58*-*2.52 (m, 1H), 2.40*-*2.31 (m, 2H), 2.20 (qd, *J =* 12.9, 4.5 Hz, 1H), 2.14*-*2.06 (m, 1H), 1.79 (hept, *J =* 5.8 Hz, 4H). ^13^C-NMR (151 MHz, DMSO) δ (ppm): 173.40, 172.70, 169.53, 164.17, 157.38, 149.38, 139.07, 124.24, 120.83, 51.82, 50.10, 48.74, 32.42, 31.46, 24.44, 23.22, 21.46. ESI-HRMS *m/z*: 345.1557 [M+H]^+^.

**1.32** *N-(2,6-dioxopiperidin-3-yl)-6-((4-methyl-2-oxopiperazin-1-yl)methyl)picolinamide (****MGD-B8****)*

**MGD-B8** can be obtained by following **Scheme 2**, and obtained white solid (0.12 mg, 38.38%). ^1^H-NMR (600 MHz, DMSO-*d_6_*) δ (ppm): 10.93 (s, 1H), 8.90 (d, *J =* 8.2 Hz, 1H), 8.00 (t, *J =* 7.7 Hz, 1H), 7.96 (dd, *J =* 7.7, 1.1 Hz, 1H), 7.42 (dd, *J =* 7.8, 1.1 Hz, 1H), 4.81 (ddd, *J =* 13.0, 8.2, 5.4 Hz, 1H), 4.68 (s, 2H), 3.40 (t, *J =* 5.5 Hz, 2H), 3.06 (d, *J =* 2.9 Hz, 2H), 2.82 (ddd, *J =* 17.3, 13.7, 5.5 Hz, 1H), 2.65 (dd, *J =* 6.5, 4.7 Hz, 2H), 2.58*-*2.52 (m, 1H), 2.25 (s, 3H), 2.22*-*2.15 (m, 1H), 2.07 (dtd, *J =* 12.8, 5.4, 2.4 Hz, 1H). ^13^C-NMR (151 MHz, DMSO) δ (ppm): 173.41, 172.64, 166.84, 164.12, 156.62, 149.41, 139.19, 124.41, 120.99, 58.97, 51.54, 50.97, 50.03, 47.26, 44.86, 31.46, 24.52. ESI-HRMS *m/z*: 360.1666 [M+H]^+^.

**1.33** *N-(2,6-dioxopiperidin-3-yl)-6-((3-oxomorpholino)methyl)picolinamide (****MGD-B9****)*

**MGD-B9** can be obtained by following **Scheme 2**, and obtained white solid (0.10 g, 33.19%). ^1^H-NMR (600 MHz, DMSO-*d_6_*) δ (ppm): 10.94 (s, 1H), 8.89 (d, *J =* 8.0 Hz, 1H), 8.02 (t, *J =* 7.7 Hz, 1H), 7.97 (dd, *J =* 7.7, 1.2 Hz, 1H), 7.51 (dd, *J =* 7.7, 1.2 Hz, 1H), 4.80 (ddd, *J =* 13.0, 8.0, 5.4 Hz, 1H), 4.77*-*4.68 (m, 2H), 4.17 (s, 2H), 3.91 (t, *J =* 5.1 Hz, 2H), 3.47 (td, *J =* 4.8, 1.3 Hz, 2H), 2.82 (ddd, *J =* 17.3, 13.7, 5.6 Hz, 1H), 2.20 (qd, *J =* 12.8, 4.5 Hz, 1H), 2.09 (dtd, *J =* 12.8, 5.5, 2.5 Hz, 1H). ^13^C-NMR (151 MHz, DMSO) δ (ppm): 173.40, 172.68, 167.04, 164.12, 156.49, 149.49, 139.22, 124.62, 121.08, 67.91, 63.79, 50.97, 50.11, 47.29, 31.45, 24.44. ESI-HRMS *m/z*: 347.1350 [M+H]^+^.

**1.34** *N-(2,6-dioxopiperidin-3-yl)-6-((3-oxothiomorpholino)methyl)picolinamide (****MGD-B10****)*

**MGD-B10** can be obtained by following **Scheme 2**, and obtained white solid (0.09 mg, 28.54%). ^1^H-NMR (600 MHz, DMSO-*d_6_*) δ (ppm): 10.94 (s, 1H), 8.89 (d, *J =* 8.2 Hz, 1H), 8.01 (t, *J =* 7.7 Hz, 1H), 7.96 (dd, *J =* 7.7, 1.1 Hz, 1H), 7.49 (dd, *J =* 7.7, 1.1 Hz, 1H), 4.85*-*4.79 (m, 1H), 4.79*-*4.69 (m, 2H), 3.76 (dd, *J =* 6.6, 5.1 Hz, 2H), 3.38 (d, *J =* 4.0 Hz, 2H), 2.94 (td, *J =* 5.5, 2.1 Hz, 2H), 2.83 (ddd, *J =* 17.3, 13.7, 5.6 Hz, 1H), 2.58*-*2.52 (m, 1H), 2.20 (qd, *J =* 12.9, 4.5 Hz, 1H), 2.08 (dtd, *J =* 12.9, 5.5, 2.4 Hz, 1H). ^13^C-NMR (151 MHz, DMSO) δ (ppm): 173.40, 172.67, 167.51, 164.12, 157.02, 149.31, 139.11, 124.45, 120.92, 52.25, 50.03, 49.79, 31.46, 29.81, 26.34, 24.54. ESI-HRMS *m/z*: 363.1122 [M+H]^+^.

**1.35** *methyl 3-(bromomethyl)-2-fluorobenzoate (****4-1****)*

Methyl 2-fluoro-3-methylbenzoate (2.00 g, 11.89 mmol) and AIBN (97.70 mg, 0.60 mmol) were added to DCM (20 mL). The temperature was raised to 80 ℃, and NBS (2.33 g, 13.08 mmol) was added in batches at rt. After reaction for 1 h, TLC detection showed that the reaction was complete, and the heating was stopped and the reaction liquid was gradually cooled to 0 ℃. After 1 h, large amounts of white crystals are precipitated and filtered off leaving a liquid, which was concentrated and purified by silica gel column chromatography (PE : EA = 100 : 1) to obtain colorless oil liquid **4-1** (2.20 g, 74.83%).

**1.36** *methyl 2-fluoro-3-(morpholinomethyl)benzoate* ***(4-2)***

The reaction conditions for the synthesis of intermediates **4-2** can be divided into two categories: when the R group is a nitrogenous fatty heterocycle, potassium carbonate is selected; When the R group was a lactam ring, a strong base was used instead of sodium hydride for the reaction. Taking the synthesis of compound **MGD-C3** as an example,**4-1** (1.20 g, 0.87 mmol) was dissolved in EA (10 mL), and morpholine (175.30 mg, 1.75 mmol) was added, and the reaction was maintained for 60℃ for about 1 h. TLC test shows that the raw material has been completely reacted, a large amount of solid precipitates were removed by filtration, and the remaining liquid was concentrated under reduced pressure to obtain the intermediate **4-2**, which can be directly used for the next reaction.

**1.37** *2-fluoro-3-(morpholinomethyl)benzoicacid (****4-3****)*

Dissolve **4-2** (0.87 mmol) of the colorless oily liquid obtained in the previous step in a mixed solution of 5 mL methanol and 5 mL H_2_O, add NaOH (69.60 mg, 1.74 mmol) at rt for about 1 h, the TLC test reaction is complete, 3 water solubility is excellent, and directly dry the solvent to obtain a white solid. Do not do purification directly for the next reaction.

**1.38** *N-(2,6-dioxopiperidin-3-yl)-2-fluoro-3-(morpholinomethyl)benzamide (****MGD-C3****)*

The white solid obtained in the previous step (crude product, 0.87 mmol) was suspended in 10 mL anhydrous dichloromethane, and HATU (418.30 mg, 1.10 mmol) and triethylamine (238.30 μL, 1.74 mmol) were added under the ice bath, stirring for 30 min. Furthermore, 3-aminopiperidine-2,6-dione (181.10 mg, 1.10 mmol) was added and transferred to rt for overnight reaction. TLC test showed that after the reaction was complete, the reaction liquid was washed with water (2 × 3 mL). The organic solution was evaporated and further purified via silica gel column chromatography, yielding a white solid compound. ***MGD-C3*** (0.18 g, 59.22%). ^1^H-NMR (600 MHz, DMSO-*d_6_*) δ (ppm): 10.87 (s, 1H), 8.59 (dd, *J =* 8.2, 2.6 Hz, 1H), 7.56 (dtd, *J =* 14.6, 7.2, 1.9 Hz, 2H), 7.27 (t, *J =* 7.6 Hz, 1H), 4.77 (ddd, *J =* 12.3, 8.2, 5.3 Hz, 1H), 3.57 (t, *J =* 4.6 Hz, 4H), 3.55 (s, 2H), 2.79 (ddd, *J =* 17.2, 13.3, 5.6 Hz, 1H), 2.55 (dd, *J =* 4.5, 2.9 Hz, 1H), 2.40 (t, *J =* 4.8 Hz, 4H), 2.13*-*2.05 (m, 1H), 2.01 (dtd, *J =* 12.8, 5.5, 2.8 Hz, 1H). ^13^C-NMR (151 MHz, DMSO) δ (ppm): 173.43, 172.35, 164.17, 159.08-157.42, 134.44, 129.65 (2C), 124.44-124.41, 123.95-123.85, 66.56, 55.36, 53.46 (2C), 50.08 (2C), 31.37, 24.50. ESI-HRMS *m/z*: 350.1511 [M+H]^+^.

**1.39** *N-(2,6-dioxopiperidin-3-yl)-2-fluoro-3-(piperidin-1-ylmethyl)benzamide* ***(MGD-C1)***

**MGD-C1** can be obtained by following **Scheme 3**, and obtained white solid (0.17 g, 58.62%). ^1^H-NMR (600 MHz, DMSO- *d_6_*) δ (ppm): 10.87 (s, 1H), 8.63*-*8.56 (m, 1H), 7.57 (t, *J =* 7.2 Hz, 2H), 7.27 (t, *J =* 7.7 Hz, 1H), 4.77 (ddd, *J =* 12.4, 8.2, 5.4 Hz, 1H), 3.29 (s, 4H), 2.79 (ddd, *J =* 17.3, 13.3, 5.6 Hz, 1H), 2.57*-*2.53 (m, 1H), 2.15*-*1.96 (m, 2H), 1.72 (s, 4H). ^13^C-NMR (151 MHz, DMSO) δ (ppm): 173.43, 172.33, 164.15, 158.81-157.15, 134.23, 129.70 (2C), 124.54-124.52, 123.94-123.84, 53.79, 52.10, 50.08 (2C), 31.37, 24.50, 23.47 (2C). ESI-HRMS *m/z*: 334.1560 [M+H]^+^.

**1.40** *N-(2,6-dioxopiperidin-3-yl)-2-fluoro-3-((4-methylpiperazin-1-yl)methyl)benzamide* ***(MGD-C2)***

**MGD-C2** can be obtained by following **Scheme 3**, and obtained white solid (0.11 g, 53.92%). ^1^H-NMR (600 MHz, DMSO-*d_6_*) δ (ppm): 10.87 (s, 1H), 8.62 (dd, *J =* 8.2, 2.5 Hz, 1H), 7.61*-*7.57 (m, 1H), 7.55 (t, *J =* 7.2 Hz, 1H), 7.29 (t, *J =* 7.6 Hz, 1H), 4.77 (ddd, *J =* 12.9, 8.2, 5.4 Hz, 1H), 3.64 (s, 2H), 3.06 (q, *J =* 7.2 Hz, 3H), 2.79 (ddd, *J =* 18.2, 13.3, 5.6 Hz, 2H), 2.70 (s, 3H), 2.55 (t, *J =* 3.8 Hz, 1H), 2.52 (s, 1H), 2.10 (qd, *J =* 12.9, 4.5 Hz, 1H), 2.04*-*1.98 (m, 1H), 1.20 (t, *J =* 7.3 Hz, 3H). ^13^C-NMR (151 MHz, DMSO) δ (ppm): 173.44, 172.35, 164.20, 159.03-157.37, 134.33-134.30, 129.54-129.52, 126.05-125.95, 124.43-124.40, 123.91-123.81, 58.68, 54.87, 52.57, 50.10, 50.07, 45.81, 45.68, 31.37, 24.49. ESI-HRMS *m/z*: 363.1826 [M+H]^+^.

**1.41** *N-(2,6-dioxopiperidin-3-yl)-2-fluoro-3-(thiomorpholinomethyl)benzamide* ***(MGD-C4)***

**MGD-C4** can be obtained by following **Scheme 3**, and obtained white solid (0.13 g, 35.58%). ^1^H-NMR (600 MHz, DMSO-*d_6_*) δ (ppm): 10.87 (s, 1H), 8.59 (dd, *J =* 8.4, 2.5 Hz, 1H), 7.55 (ddt, *J =* 13.7, 7.2, 3.6 Hz, 2H), 7.27 (t, *J =* 7.6 Hz, 1H), 4.77 (ddd, *J =* 12.9, 8.2, 5.4 Hz, 1H), 3.58 (s, 2H), 2.79 (ddd, *J =* 17.3, 13.3, 5.6 Hz, 1H), 2.71*-*2.64 (m, 4H), 2.64*-*2.57 (m, 4H), 2.56*-*2.52 (m, 1H), 2.10 (qd, *J =* 12.9, 4.5 Hz, 1H), 2.04*-*1.98 (m, 1H). ^13^C-NMR (151 MHz, DMSO) δ (ppm): 173.43, 172.34, 164.20, 159.05-157.39, 134.27, 129.56(2C) 124.43-124.41, 123.96-123.86, 55.58, 54.74 (2C), 50.08, 31.37, 27.60(2C), 24.50. ESI-HRMS *m/z*: 366.1282 [M+H]^+^.

**1.42** *N-(2,6-dioxopiperidin-3-yl)-2-fluoro-3-((2-oxopyrrolidin-1-yl)methyl)benzamide (****MGD-C5)***

**MGD-C5** can be obtained by following **Scheme 3**, and obtained white solid (0.08 g, 26.47%). ^1^H-NMR (600 MHz, DMSO-*d_6_*) δ (ppm): 10.88 (s, 1H), 8.63 (dd, *J =* 8.3, 2.3 Hz, 1H), 7.59 (td, *J =* 7.2, 1.8 Hz, 1H), 7.41 (td, *J =* 7.3, 1.8 Hz, 1H), 7.28 (t, *J =* 7.6 Hz, 1H), 4.78 (ddd, *J =* 12.4, 8.2, 5.4 Hz, 1H), 4.46 (s, 2H), 3.29 (t, *J =* 7.0 Hz, 2H), 2.80 (ddd, *J =* 17.3, 13.3, 5.6 Hz, 1H), 2.58*-*2.52 (m, 1H), 2.30 (t, *J =* 8.1 Hz, 2H), 2.10 (qd, *J =* 12.8, 4.5 Hz, 1H), 2.02 (dtd, *J =* 12.7, 5.5, 2.9 Hz, 1H), 1.99*-*1.91 (m, 2H). ^13^C-NMR (151 MHz, DMSO) δ (ppm): 174.55, 173.43, 172.31, 164.01, 158.62-156.96, 132.86-132.83, 129.85-129.83, 125.11-125.00, 124.87-124.84, 124.13-124.03, 50.09, 46.80 (2C), 31.36, 30.58, 24.50, 17.83. ESI-HRMS *m/z*: 348.1354 [M+H]^+^.

**1.43** *N-(2,6-dioxopiperidin-3-yl)-2-fluoro-3-((2-oxopiperidin-1-yl)methyl)benzamide* ***(MGD-C6)***

**MGD-C6** can be obtained by following **Scheme 3**, and obtained white solid (0.14 g, 44.53%). ^1^H-NMR (600 MHz, DMSO-*d_6_*) δ (ppm): 10.88 (s, 1H), 8.63 (dd, *J =* 8.1, 2.3 Hz, 1H), 7.61*-*7.51 (m, 1H), 7.37 (t, *J =* 7.4 Hz, 1H), 7.27 (t, *J =* 7.6 Hz, 1H), 4.78 (ddd, *J =* 13.0, 8.2, 5.4 Hz, 1H), 4.58 (s, 2H), 3.26 (t, *J =* 5.6 Hz, 2H), 2.80 (ddd, *J =* 18.1, 13.2, 5.6 Hz, 1H), 2.58*-*2.52 (m, 1H), 2.32 (t, *J =* 6.0 Hz, 2H), 2.10 (qd, *J =* 12.8, 4.5 Hz, 1H), 2.03 (ddt, *J =* 10.3, 7.6, 3.0 Hz, 1H), 1.75 (dq, *J =* 8.9, 5.5 Hz, 4H). ^13^C-NMR (151 MHz, DMSO) δ (ppm): 173.43, 172.31, 169.35, 164.09, 158.66-157.01, 132.12-132.09, 129.40-129.39, 125.75-125.64, 124.79-124.76, 124.05-123.95, 50.08, 47.98, 43.62, 32.47, 31.36, 24.51, 23.14, 21.41. ESI-HRMS *m/z*: 362.1512 [M+H]^+^.

**1.44** *N-(2,6-dioxopiperidin-3-yl)-2-fluoro-3-((3-oxomorpholino)methyl)benzamide* ***(MGD-C7)***

**MGD-B7** can be obtained by following **Scheme 3**, and obtained white solid (0.12 g, 37.96%). ^1^H-NMR (600 MHz, DMSO-*d_6_*) δ (ppm): 10.87 (s, 1H), 8.64 (dd, *J =* 8.2, 2.2 Hz, 1H), 7.58 (td, *J =* 7.2, 1.8 Hz, 1H), 7.44 (td, *J =* 7.3, 1.8 Hz, 1H), 7.29 (t, *J =* 7.6 Hz, 1H), 4.78 (ddd, *J =* 12.4, 8.2, 5.4 Hz, 1H), 4.64 (s, 2H), 4.12 (s, 2H), 3.85 (dd, *J =* 6.0, 4.3 Hz, 2H), 3.33 (d, *J =* 5.1 Hz, 2H), 2.79 (ddd, *J =* 17.3, 13.2, 5.6 Hz, 1H), 2.09 (qd, *J =* 12.9, 4.5 Hz, 1H), 2.05*-*1.98 (m, 1H). ^13^C-NMR (151 MHz, DMSO) δ (ppm): 173.42, 172.30, 166.73, 164.03, 158.72-157.06, 132.69-132.67, 129.82-129.81, 124.88-124.85, 124.79, 124.18-124.09, 67.91, 63.69, 50.09, 46.46, 43.14, 31.36, 24.51. ESI-HRMS *m/z*: 364.1303 [M+H]^+^.

**1.45** *N-(2,6-dioxopiperidin-3-yl)-2-fluoro-3-((2-oxopiperidin-1-yl)methyl)benzamide* ***(MGD-C8)***

**MGD-C8** can be obtained by following **Scheme 3**, and obtained white solid (0.14 g, 44.53%). ^1^H-NMR (600 MHz, DMSO-*d_6_*) δ (ppm): 10.88 (s, 1H), 8.63 (dd, *J =* 8.1, 2.3 Hz, 1H), 7.61*-*7.51 (m, 1H), 7.37 (t, *J =* 7.4 Hz, 1H), 7.27 (t, *J =* 7.6 Hz, 1H), 4.78 (ddd, *J =* 13.0, 8.2, 5.4 Hz, 1H), 4.58 (s, 2H), 3.26 (t, *J =* 5.6 Hz, 2H), 2.80 (ddd, *J =* 18.1, 13.2, 5.6 Hz, 1H), 2.58*-*2.52 (m, 1H), 2.32 (t, *J =* 6.0 Hz, 2H), 2.10 (qd, *J =* 12.8, 4.5 Hz, 1H), 2.03 (ddt, *J =* 10.3, 7.6, 3.0 Hz, 1H), 1.75 (dq, *J =* 8.9, 5.5 Hz, 4H). ^13^C-NMR (151 MHz, DMSO) δ (ppm): 173.43, 172.31, 169.35, 164.09, 158.66-157.01, 132.12-132.09, 129.40-129.39, 125.75-125.64, 124.79-124.76, 124.05-123.95, 50.08, 47.98, 43.62, 32.47, 31.36, 24.51, 23.14, 21.41. ESI-HRMS *m/z*: 362.1512 [M+H]^+^.

**1.46** *N-(2,6-dioxopiperidin-3-yl)-2-fluoro-3-((4-methyl-2-oxopiperazin-1-yl)methyl)benzamide* ***(MGD-C9)***

**MGD-C9** can be obtained by following **Scheme 3**, and obtained white solid (0.07 g, 21.38%). ^1^H-NMR (600 MHz, DMSO-*d_6_*) δ (ppm): 10.88 (s, 1H), 8.64 (dd, *J =* 8.2, 2.2 Hz, 1H), 7.57 (td, *J =* 7.2, 1.8 Hz, 1H), 7.38 (td, *J =* 7.3, 1.8 Hz, 1H), 7.28 (t, *J =* 7.6 Hz, 1H), 4.78 (ddd, *J =* 12.3, 8.2, 5.4 Hz, 1H), 4.61 (s, 2H), 3.27 (dd, *J =* 6.3, 4.7 Hz, 2H), 3.04 (s, 2H), 2.80 (ddd, *J =* 17.2, 13.2, 5.6 Hz, 1H), 2.64*-*2.59 (m, 2H), 2.55 (td, *J =* 3.3, 1.6 Hz, 1H), 2.23 (s, 3H), 2.10 (qd, *J =* 12.9, 4.5 Hz, 1H), 2.06*-*1.98 (m, 1H). ^13^C-NMR (151 MHz, DMSO) δ (ppm): 177.69, 174.99, 173.41, 172.70, 164.11, 156.69, 149.46, 139.21, 124.62, 121.06, 50.12, 47.70, 41.83, 31.45, 30.57, 24.42, 20.88, 18.09. ESI-HRMS *m/z*: 377.1620 [M+H]^+^.

**2. ^1^H NMR and ^13^C NMR of MGD-AX, MGD-BX, MGD-CX analogues.**


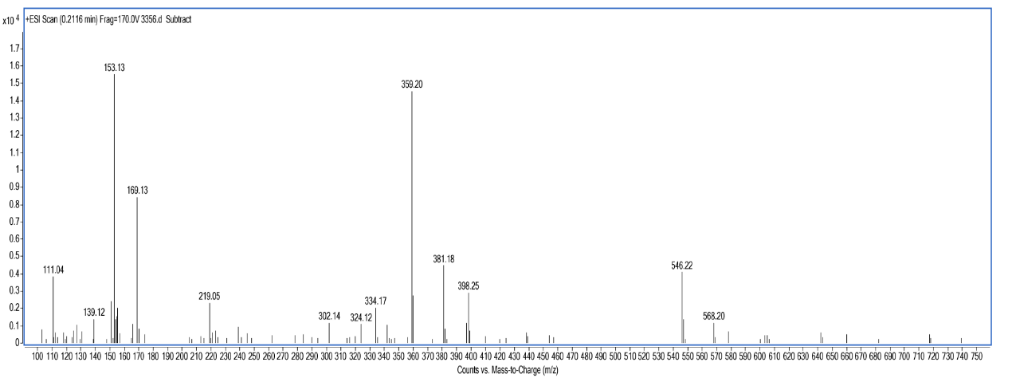


ESI-MS spectra of **MGD-A1**


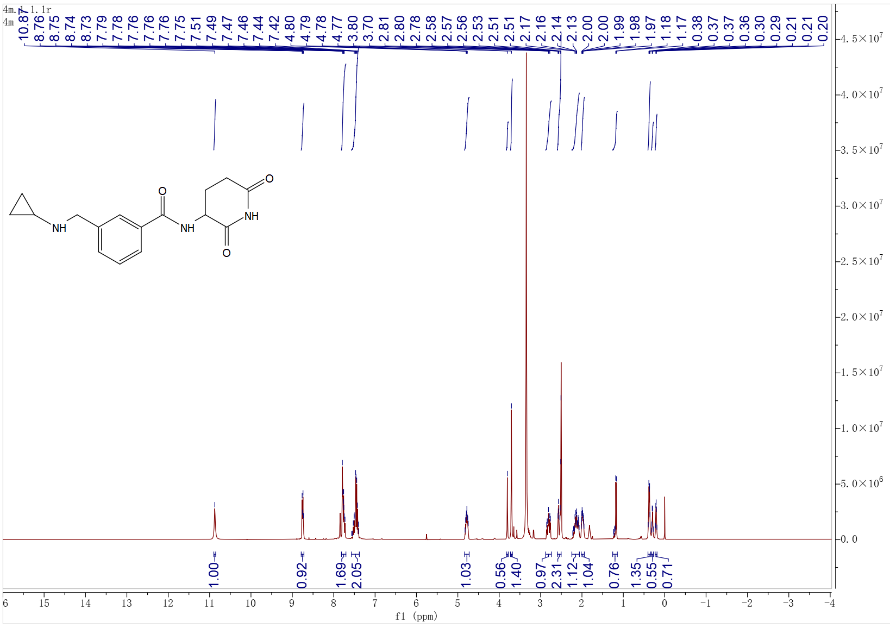


^1^H-NMR spectra of **MGD-A1**


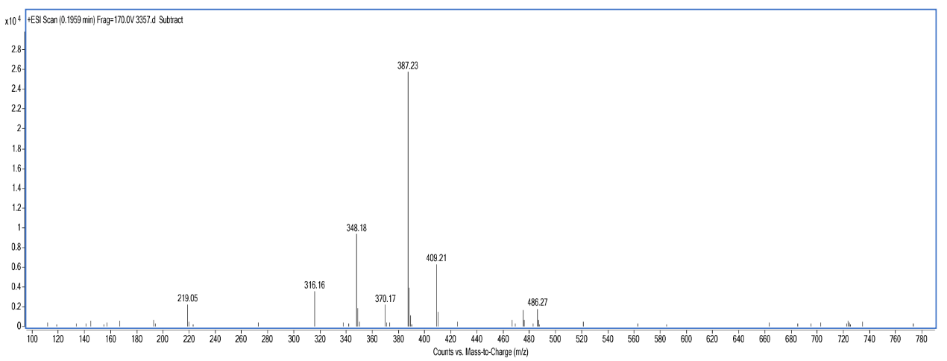


ESI-MS spectra of **MGD-A2**


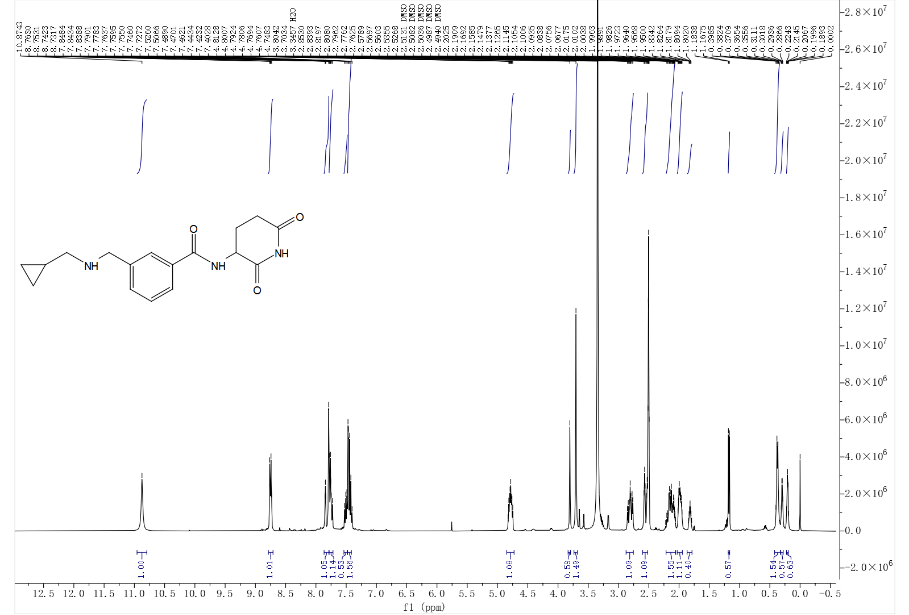


^1^H-NMR spectra of **MGD-A2**


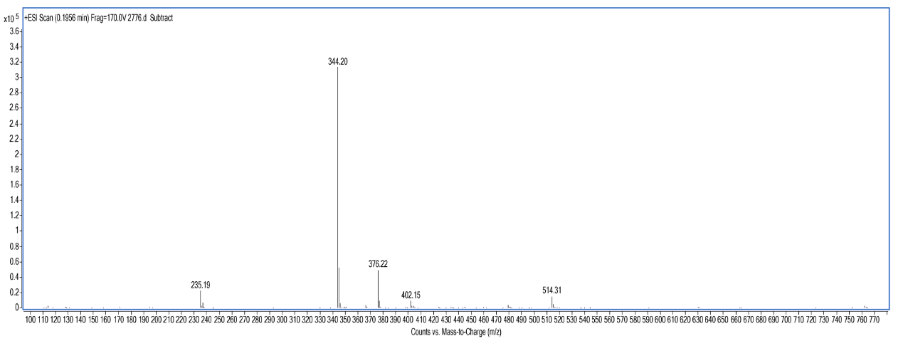


ESI-MS spectra of **MGD-A3**


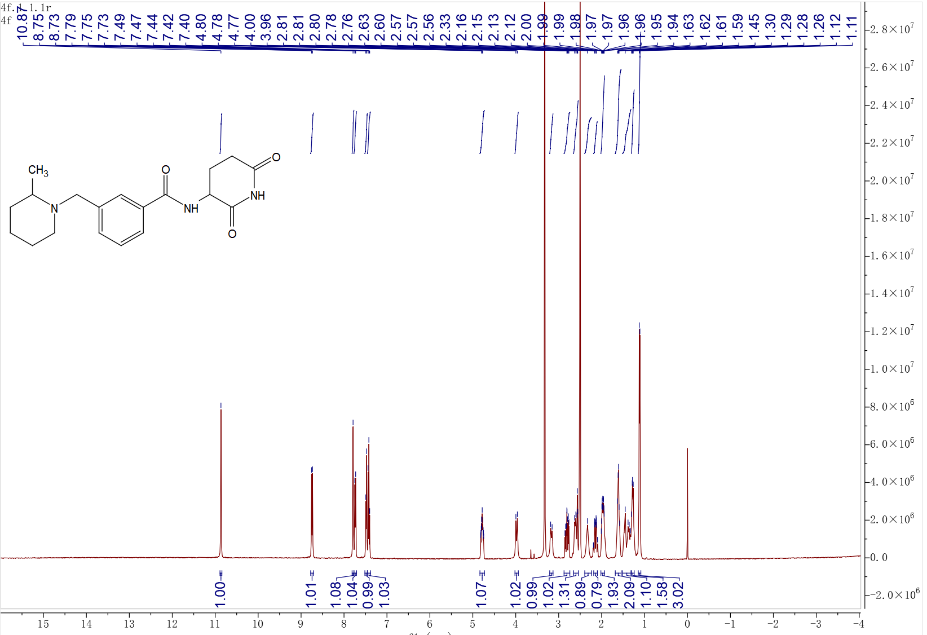


^1^H-NMR spectra of **MGD-A3**


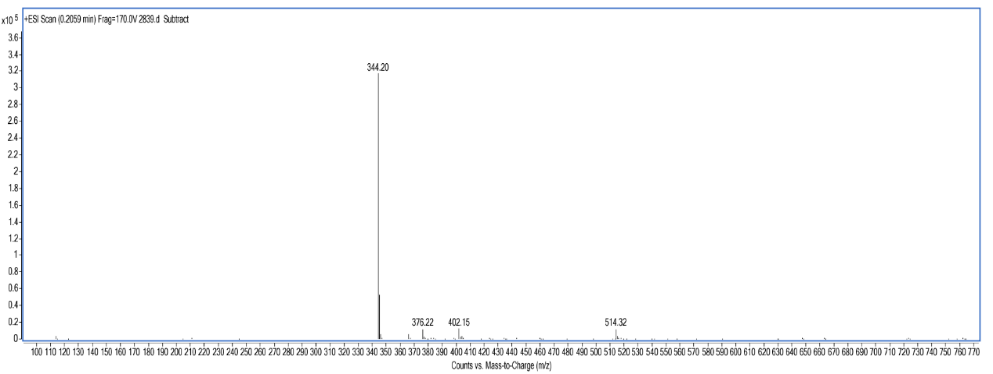


ESI-MS spectra of **MGD-A4**


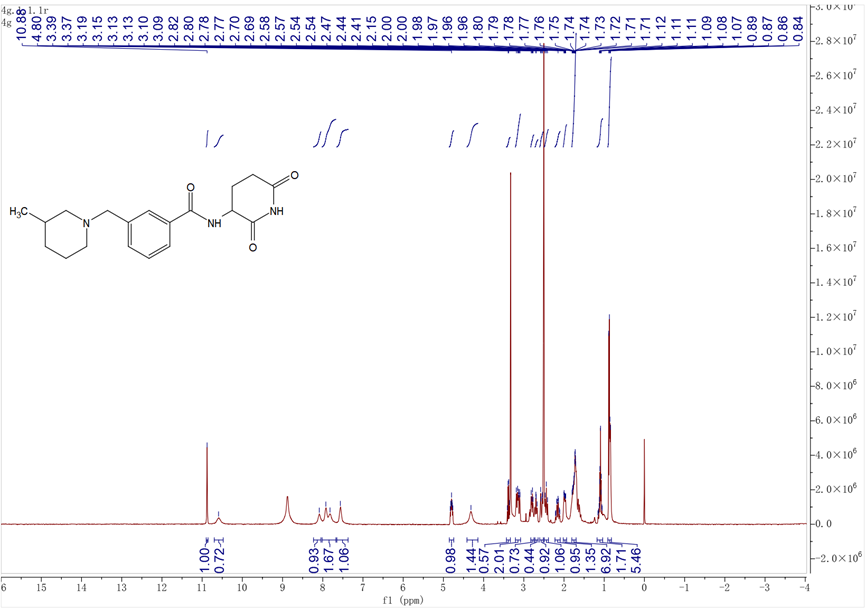


^1^H-NMR spectra of **MGD-A4**


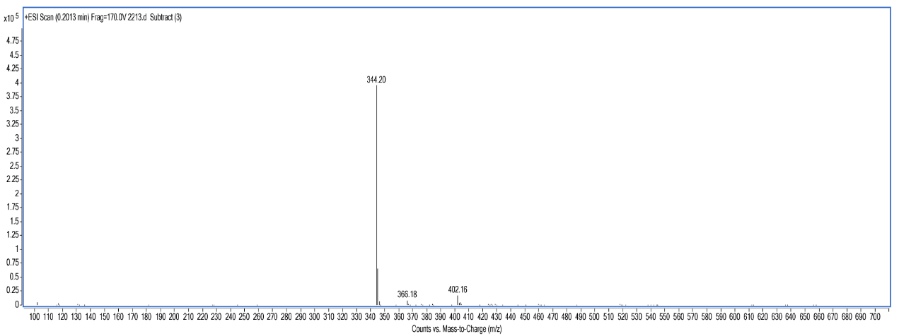


ESI-MS spectra of **MGD-A5**


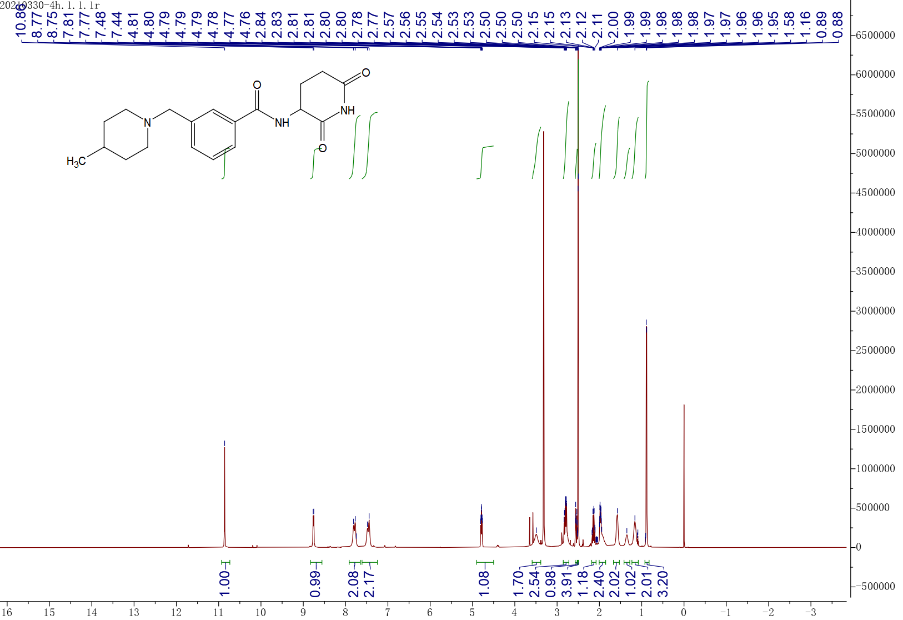


^1^H-NMR spectra of **MGD-A5**


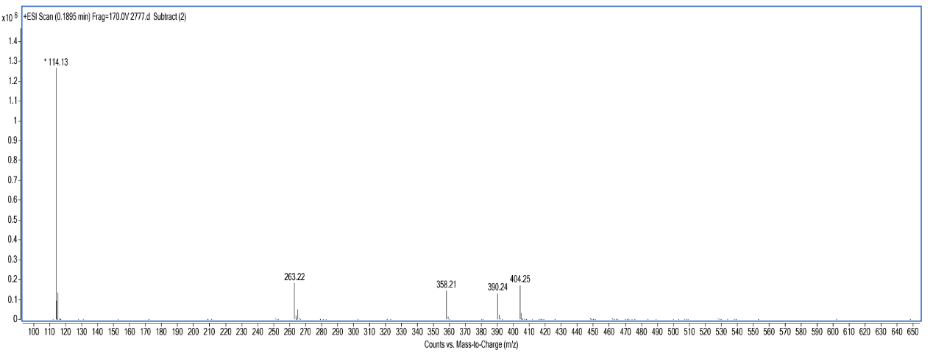


ESI-MS spectra of **MGD-A6**


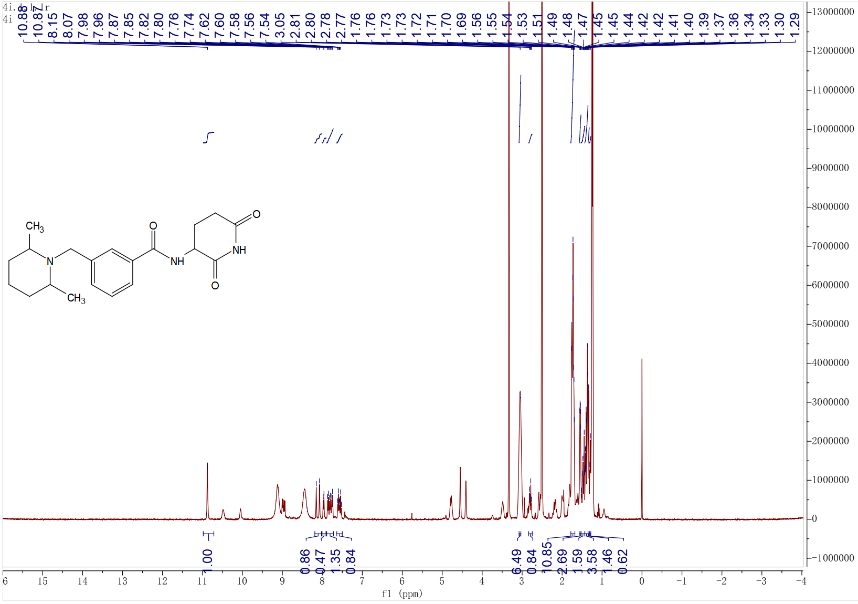


^1^H-NMR spectra of **MGD-A6**


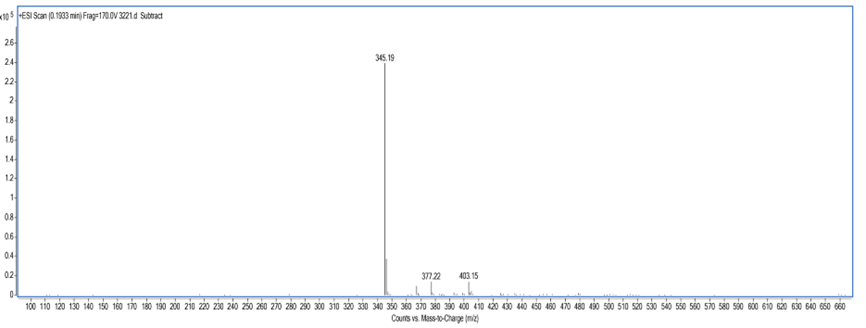


ESI-MS spectra of **MGD-A7**


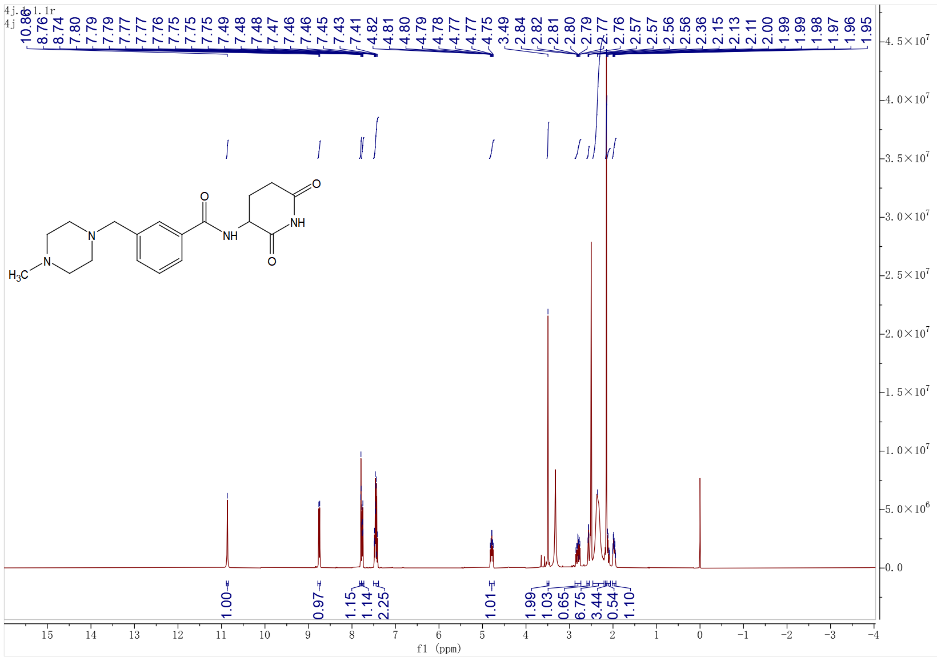


^1^H-NMR spectra of **MGD-A7**


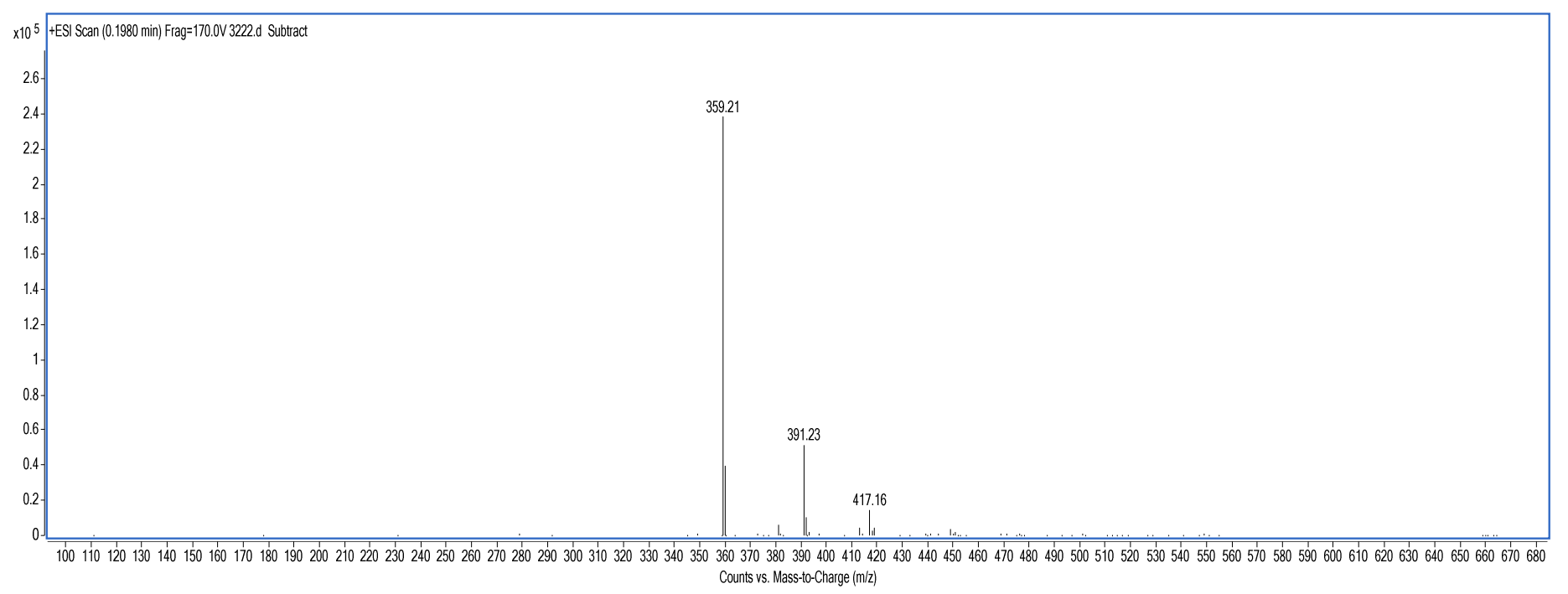


ESI-MS spectra of **MGD-A8**


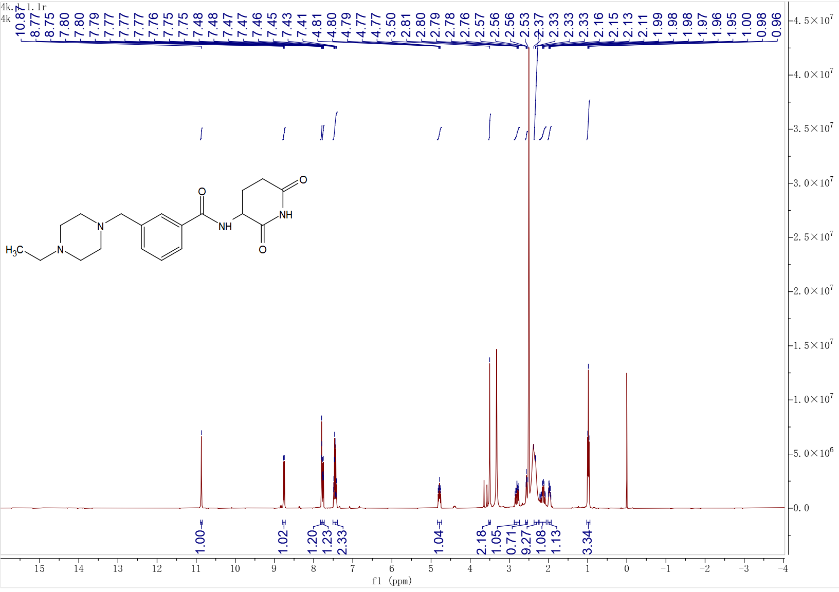


^1^H-NMR spectra of **MGD-A8**


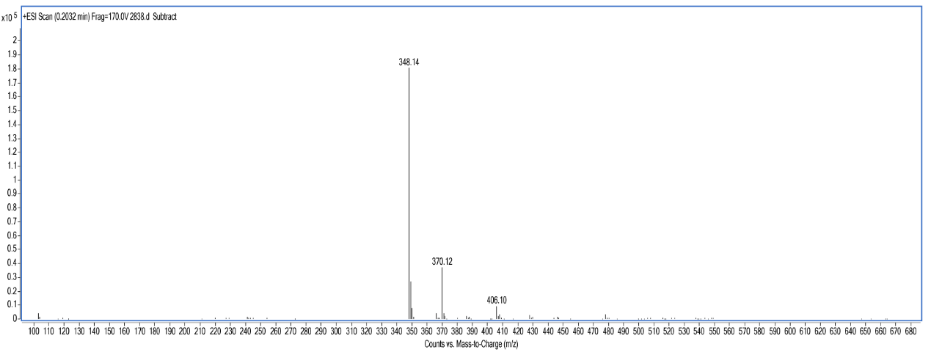


ESI-MS spectra of **MGD-A9**


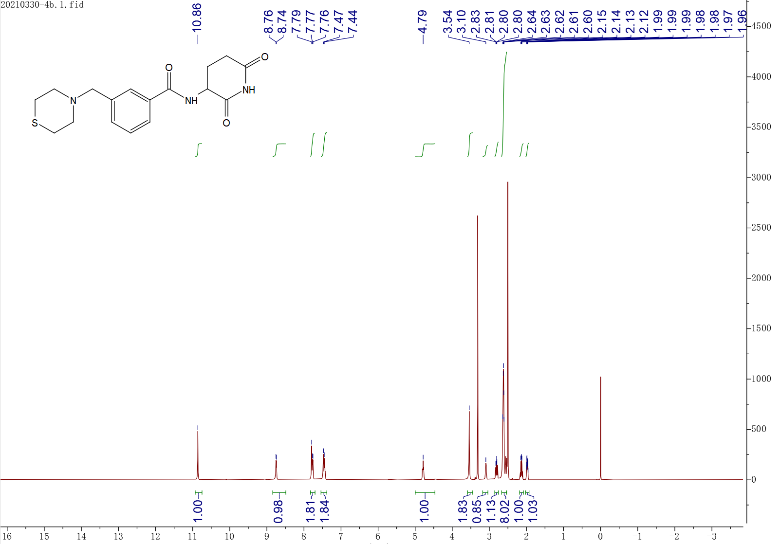


^1^H-NMR spectra of **MGD-A9**


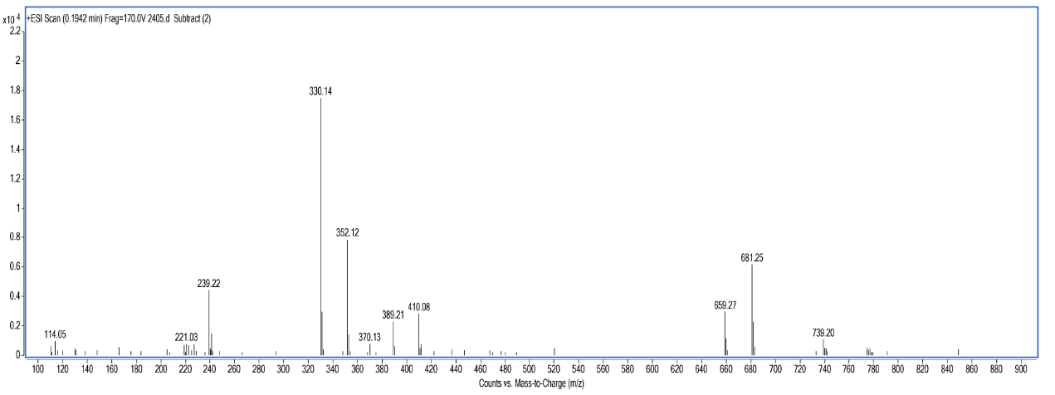


ESI-MS spectra of **MGD-A10**


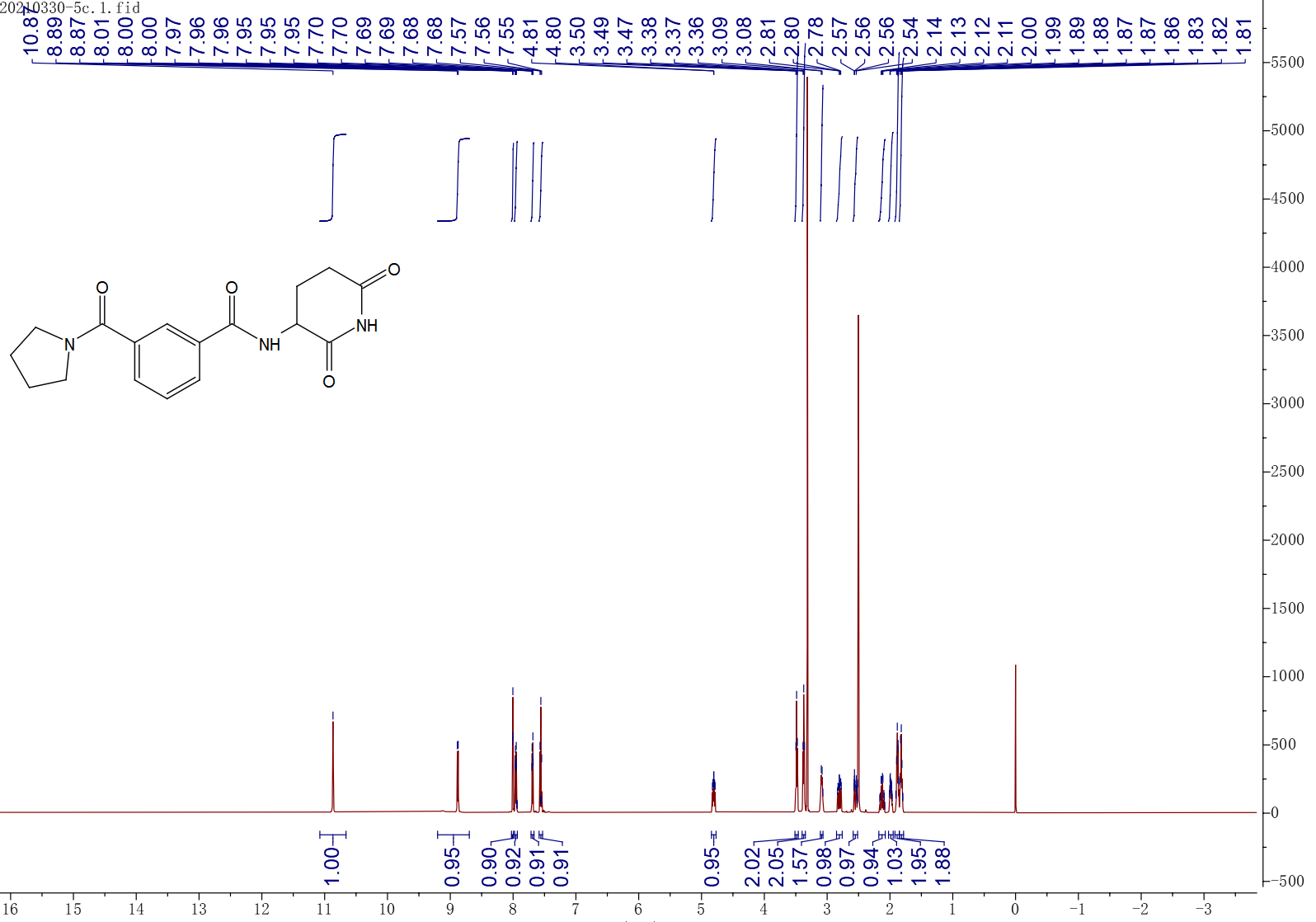


^1^H-NMR spectra of **MGD-A10**


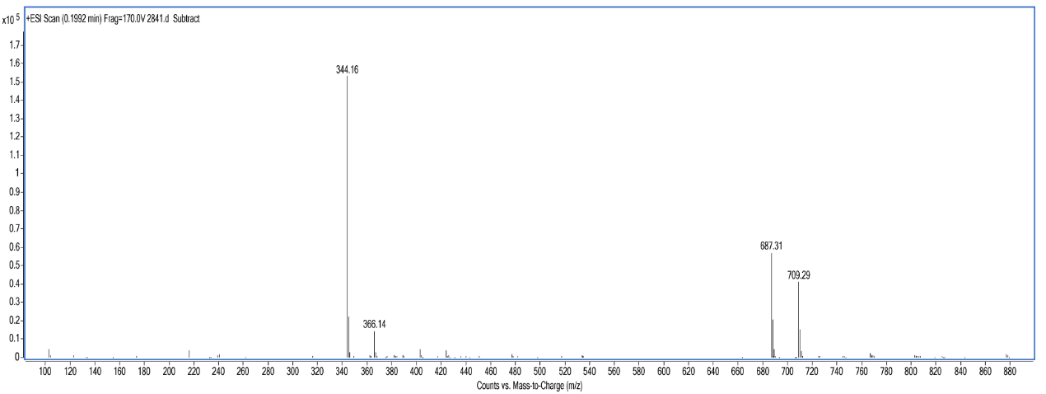


ESI-MS spectra of **MGD-A11**


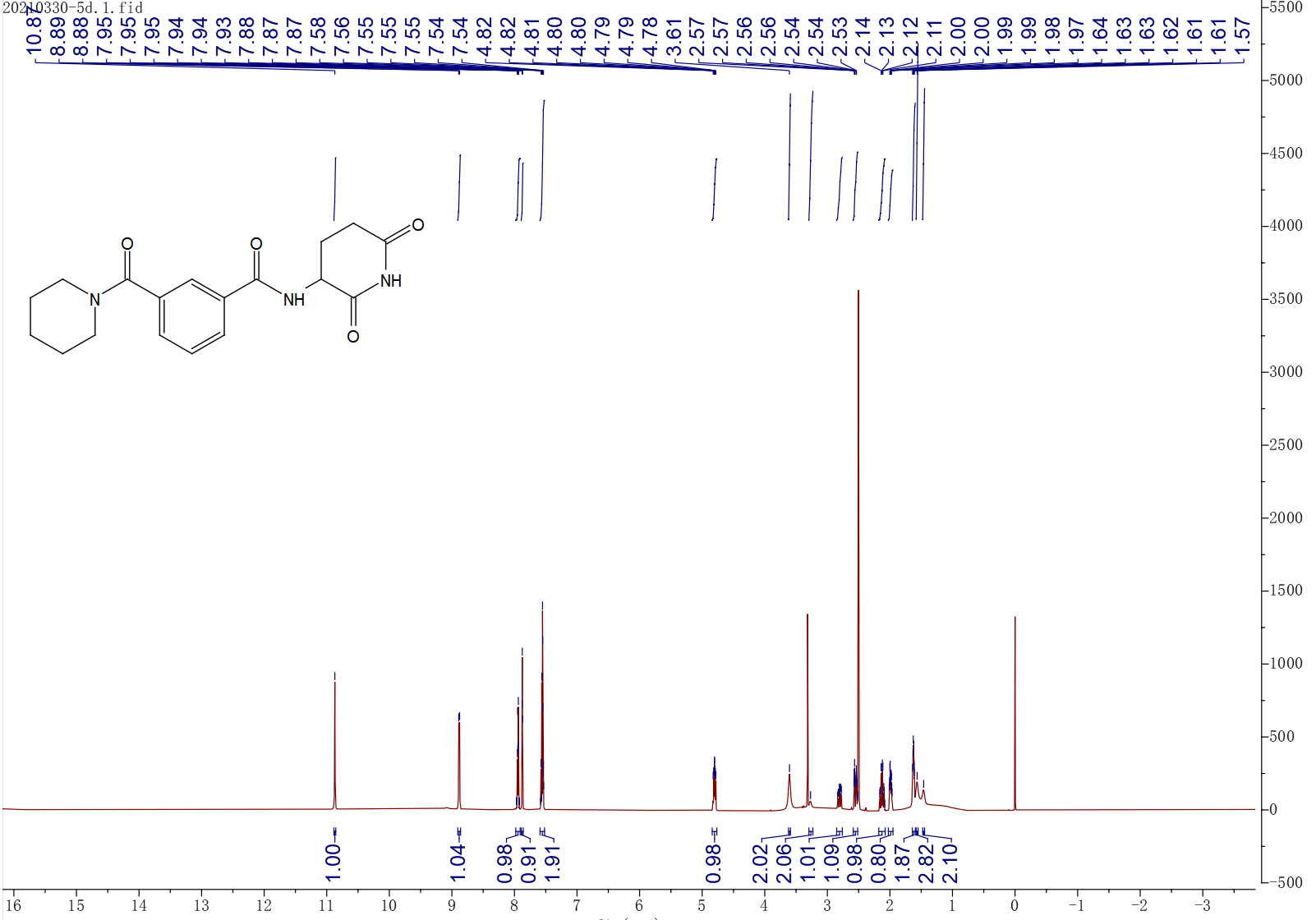


^1^H-NMR spectra of **MGD-A11**


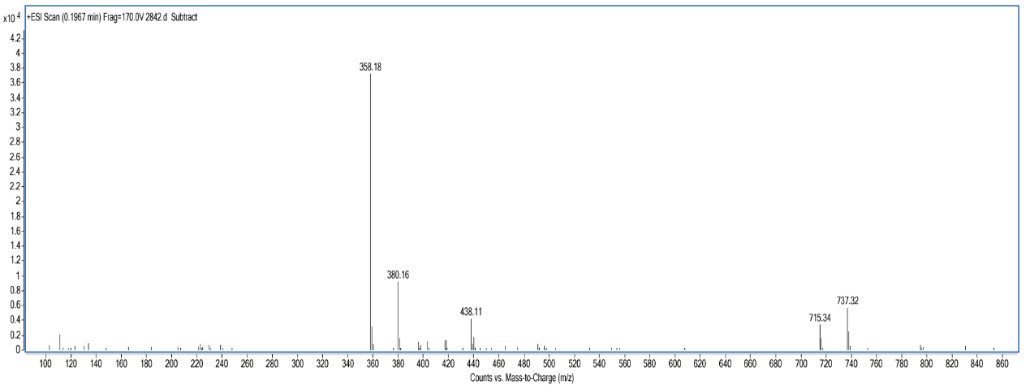


ESI-MS spectra of **MGD-A12**


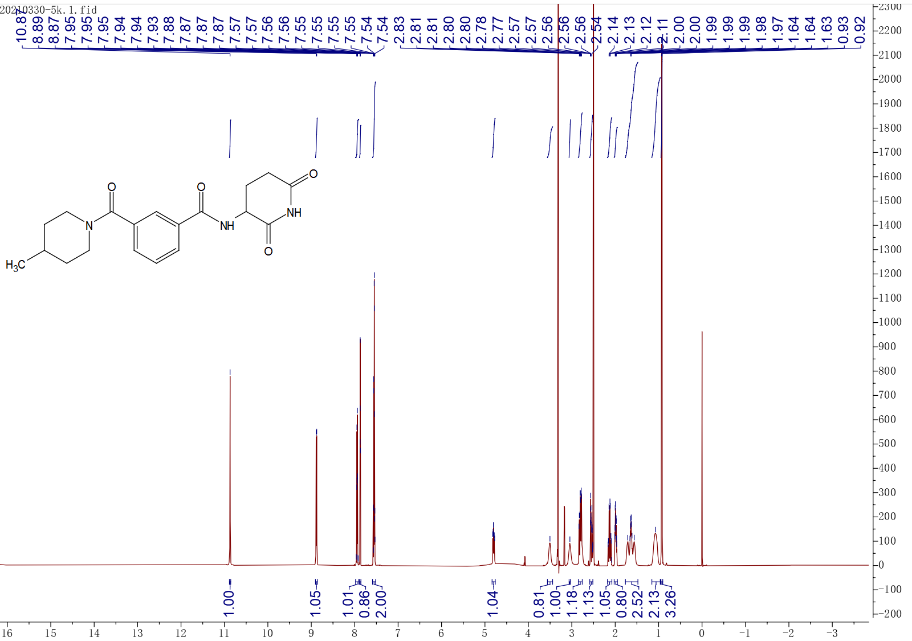


^1^H-NMR spectra of **MGD-A12**


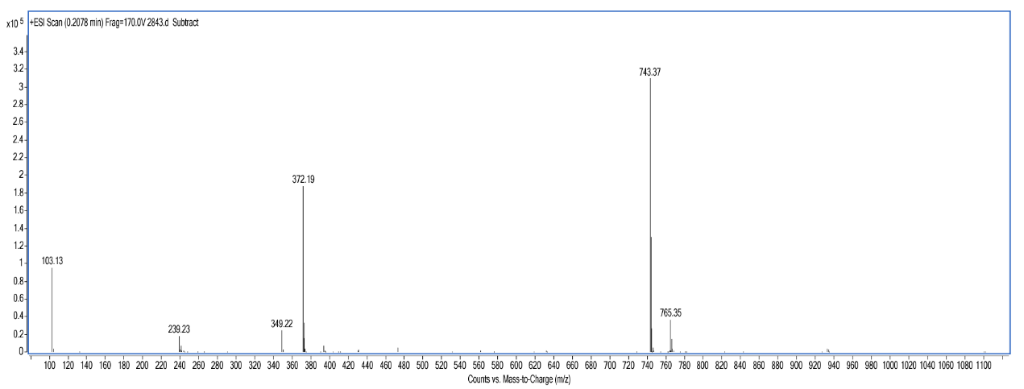


ESI-MS spectra of **MGD-A13**


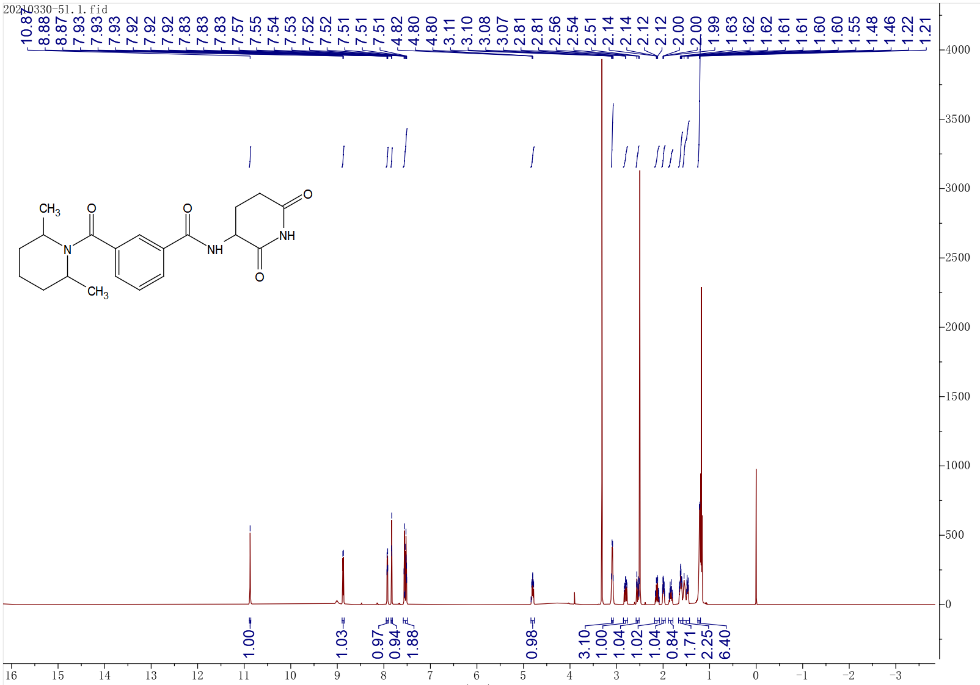


^1^H-NMR spectra of **MGD-A13**


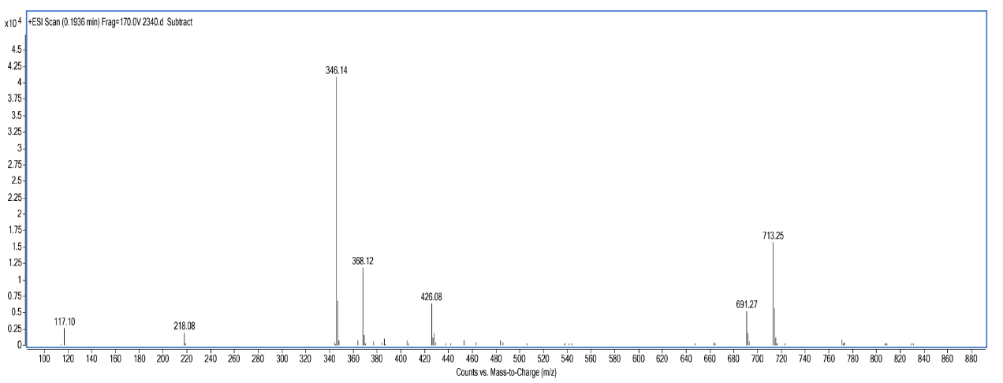


ESI-MS spectra of **MGD-A14**


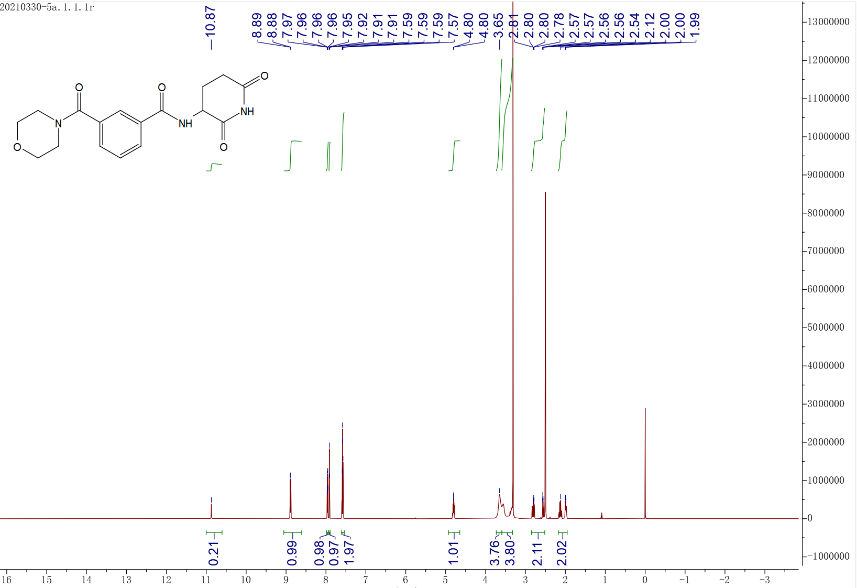


^1^H-NMR spectra of **MGD-A14**


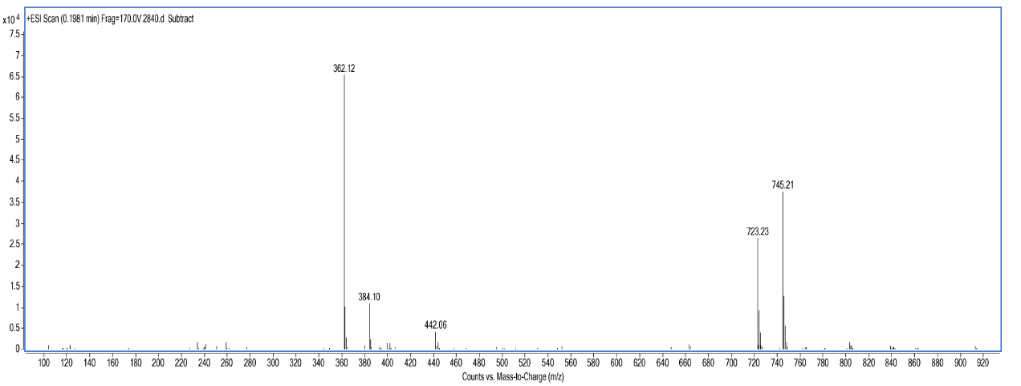


ESI-MS spectra of **MGD-A15**


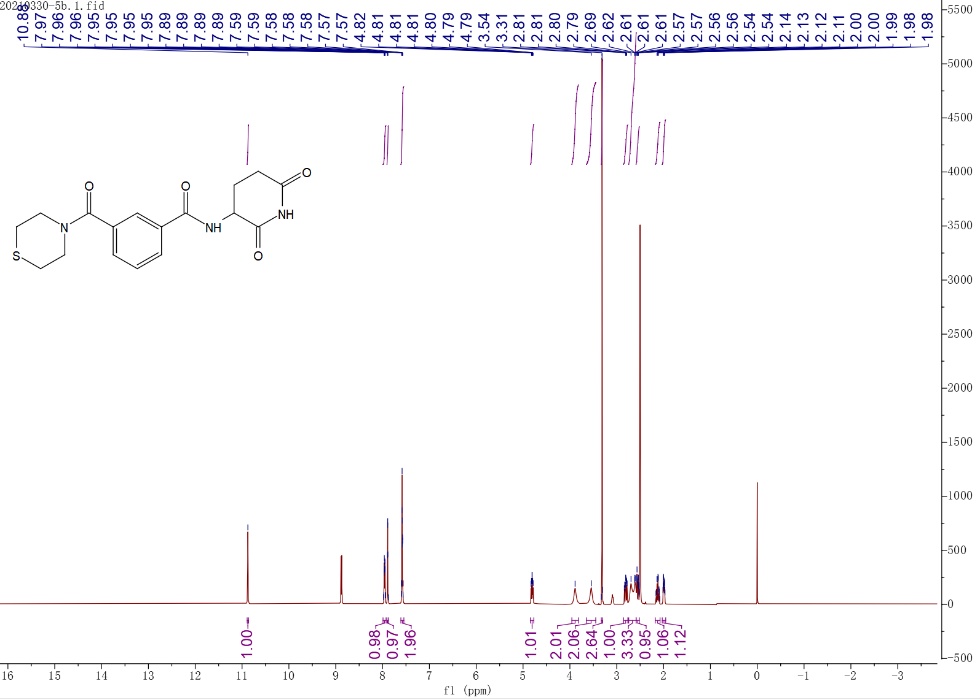


^1^H-NMR spectra of **MGD-A15**


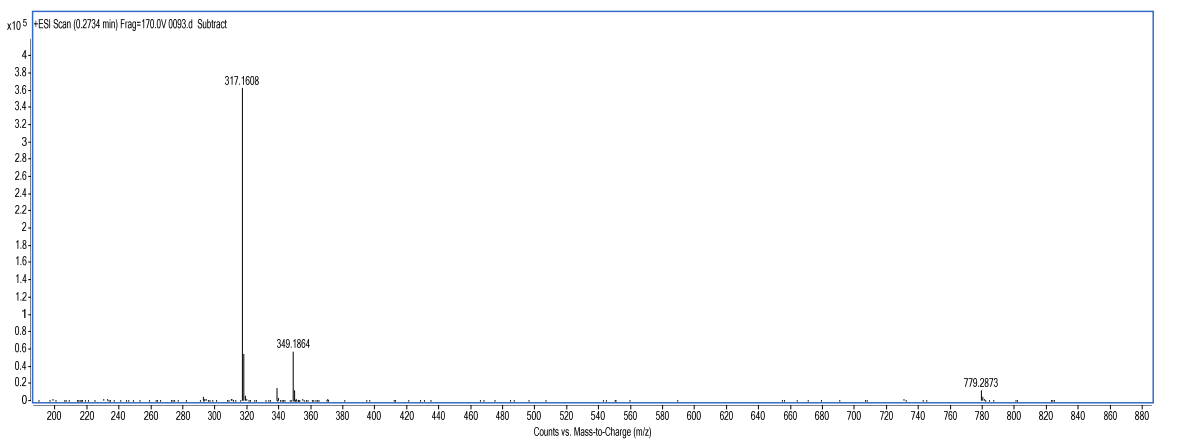


ESI-MS spectra of **MGD-B1**


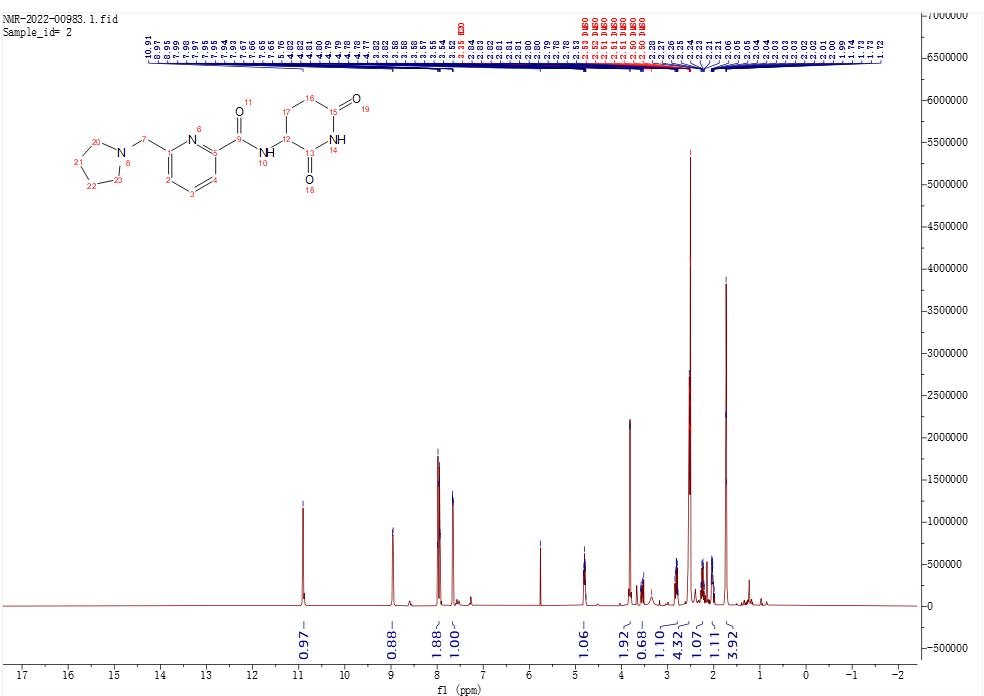


^1^H-NMR spectra of **MGD-B1**


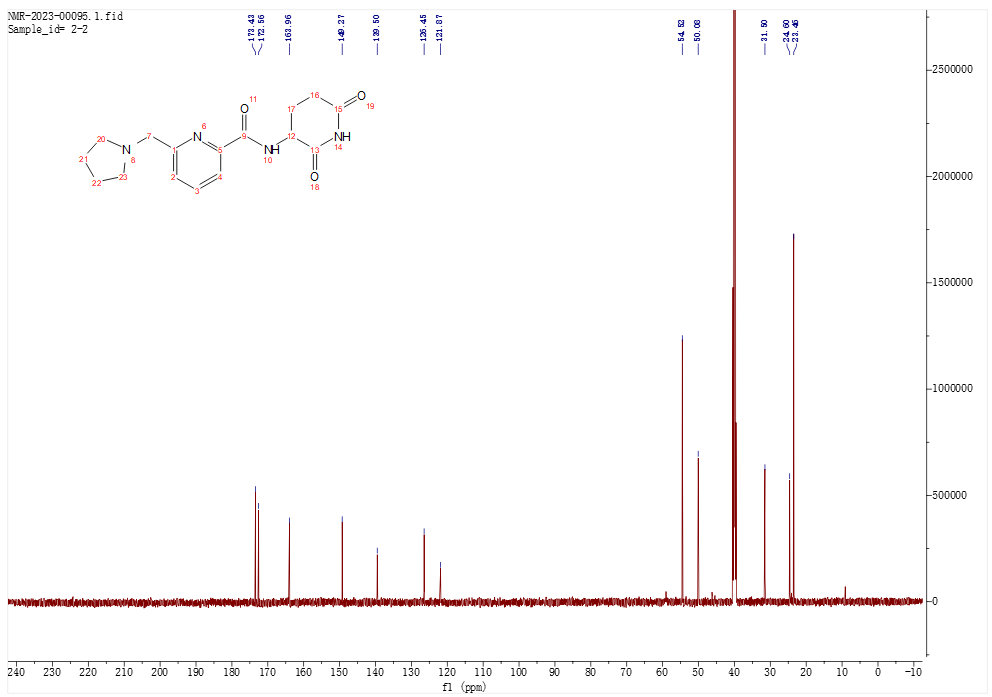


^13^C-NMR spectra of **MGD-B1**


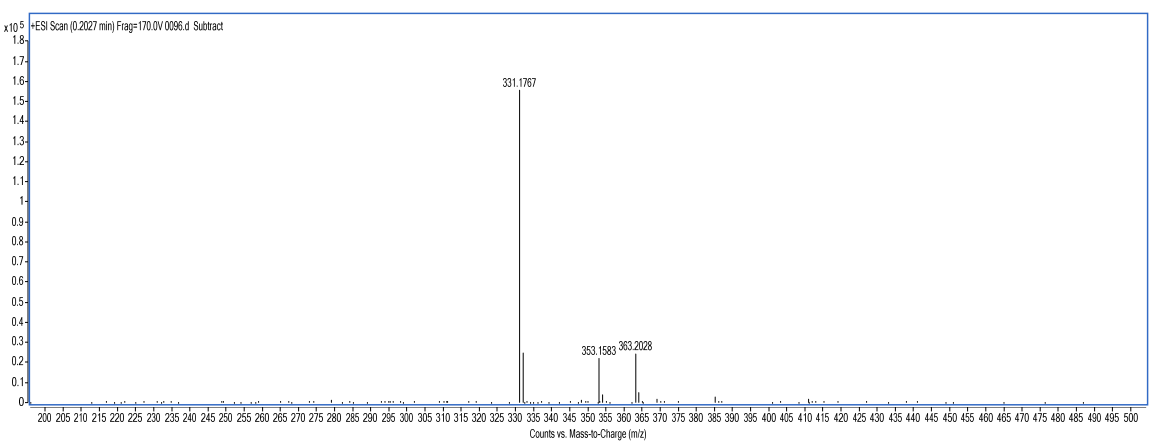


ESI-MS spectra of **MGD-B2**


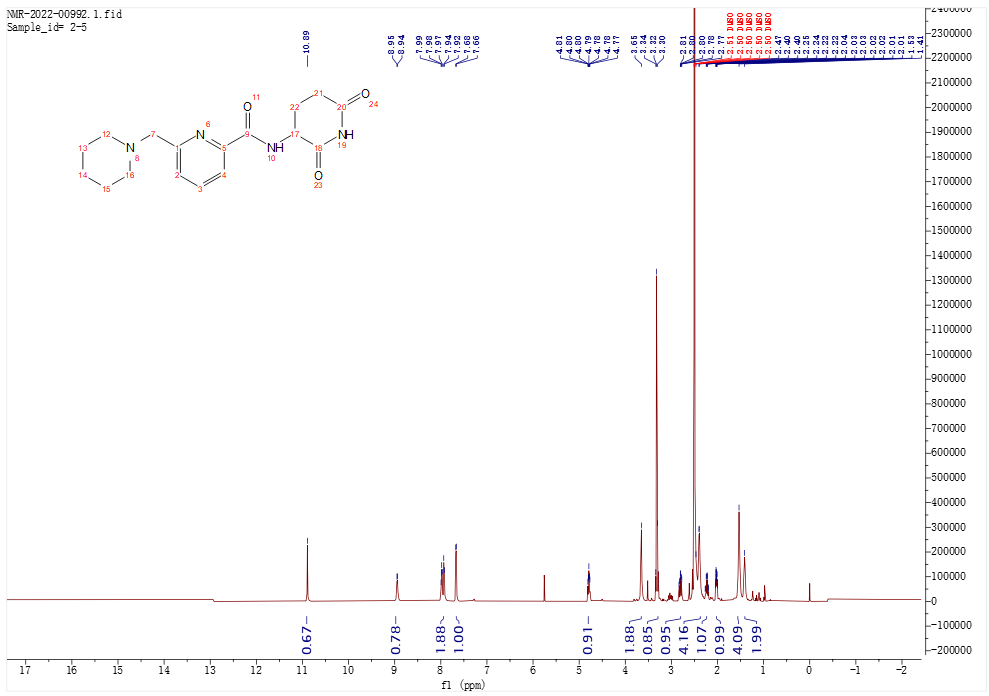


^1^H-NMR spectra of **MGD-B2**


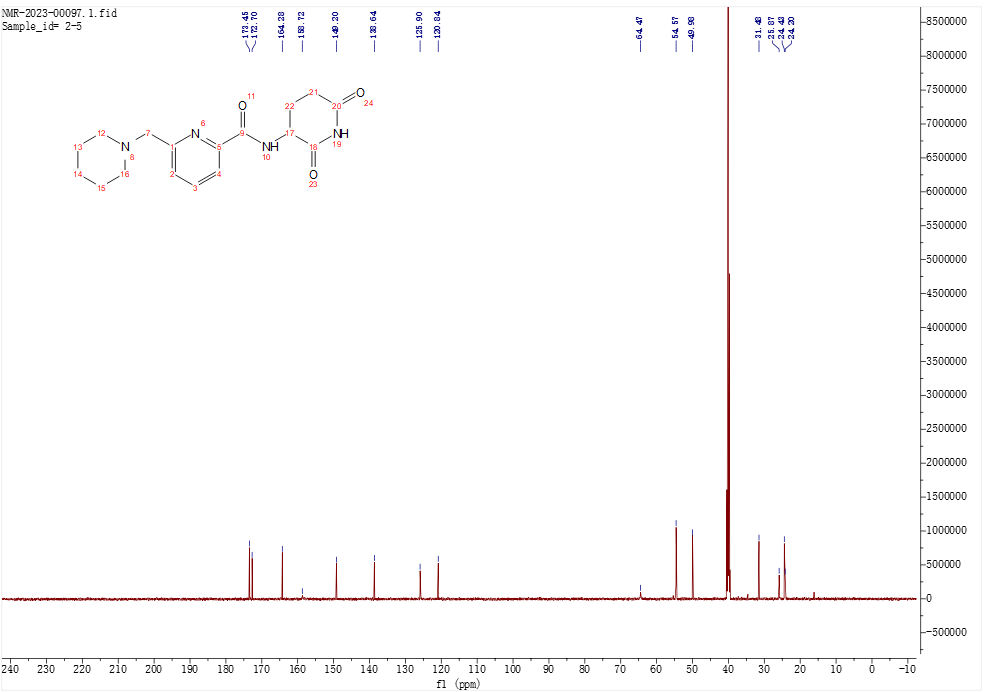


^13^C-NMR spectra of **MGD-B2**


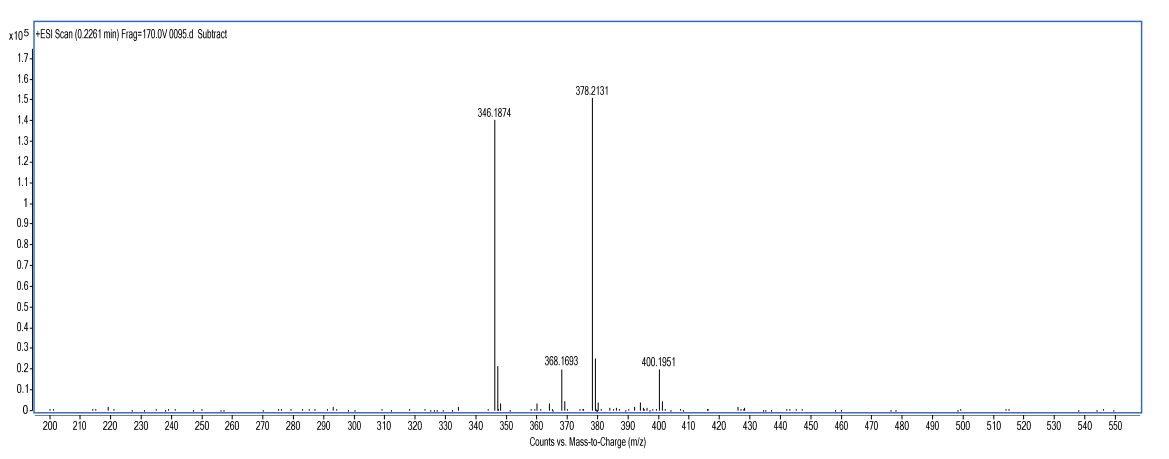


ESI-MS spectra of **MGD-B3**


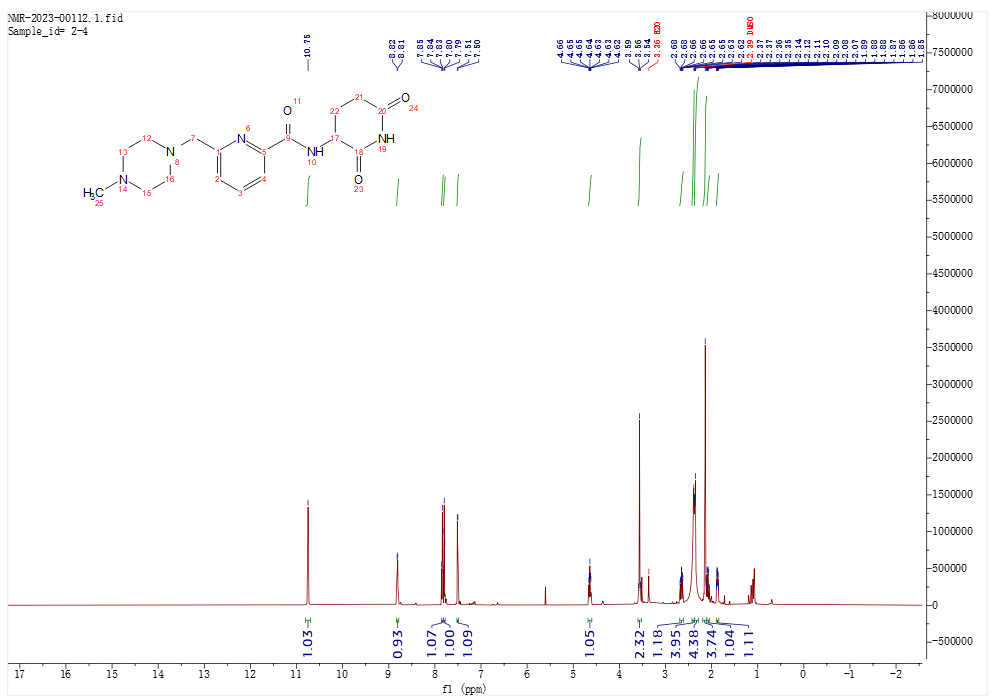


^1^H-NMR spectra of **MGD-B3**


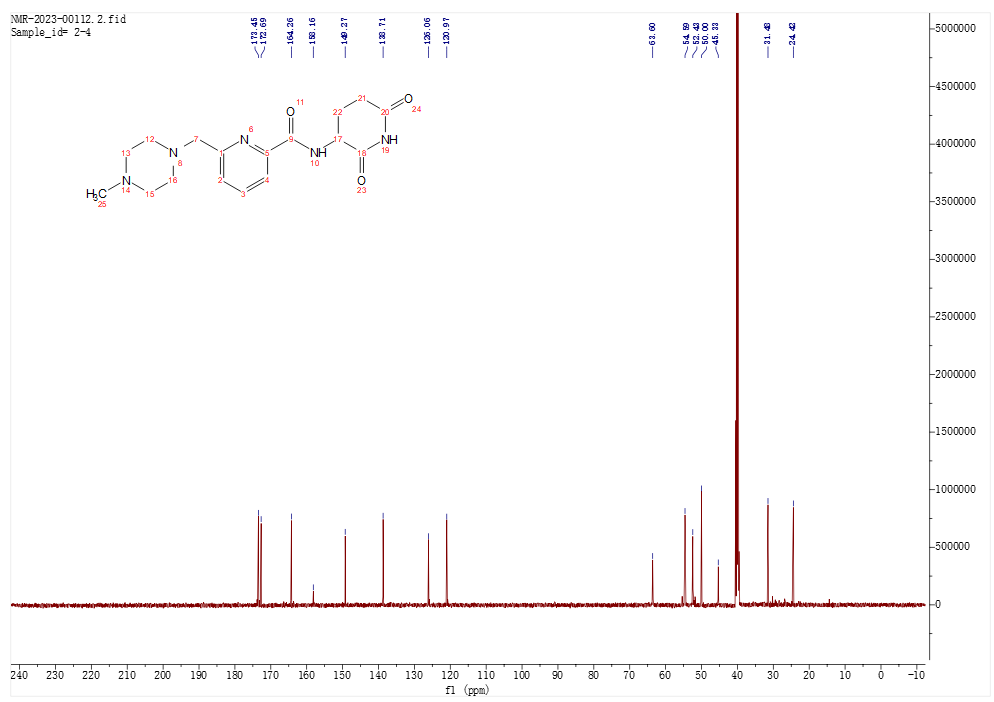


^13^C-NMR spectra of **MGD-B3**


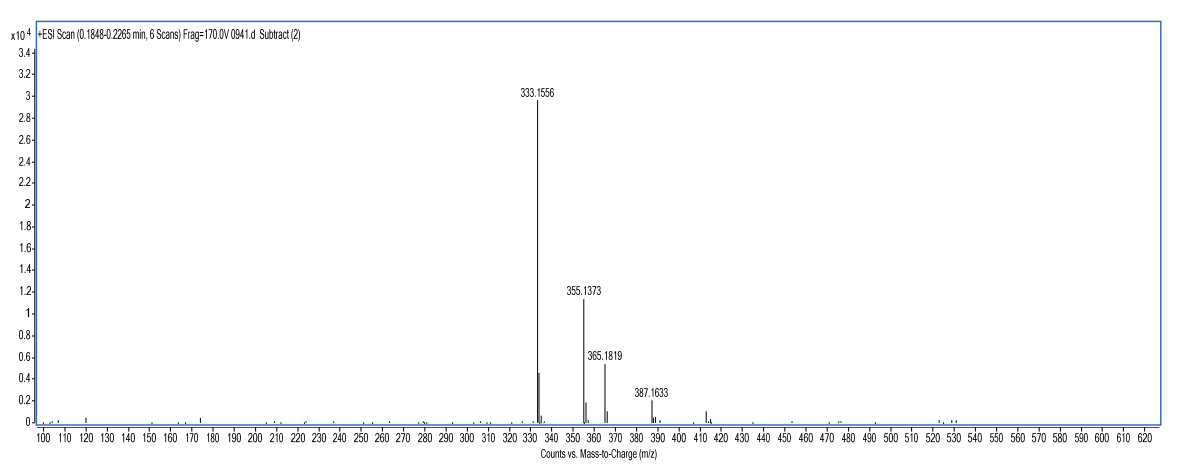


ESI-MS spectra of **MGD-B4**


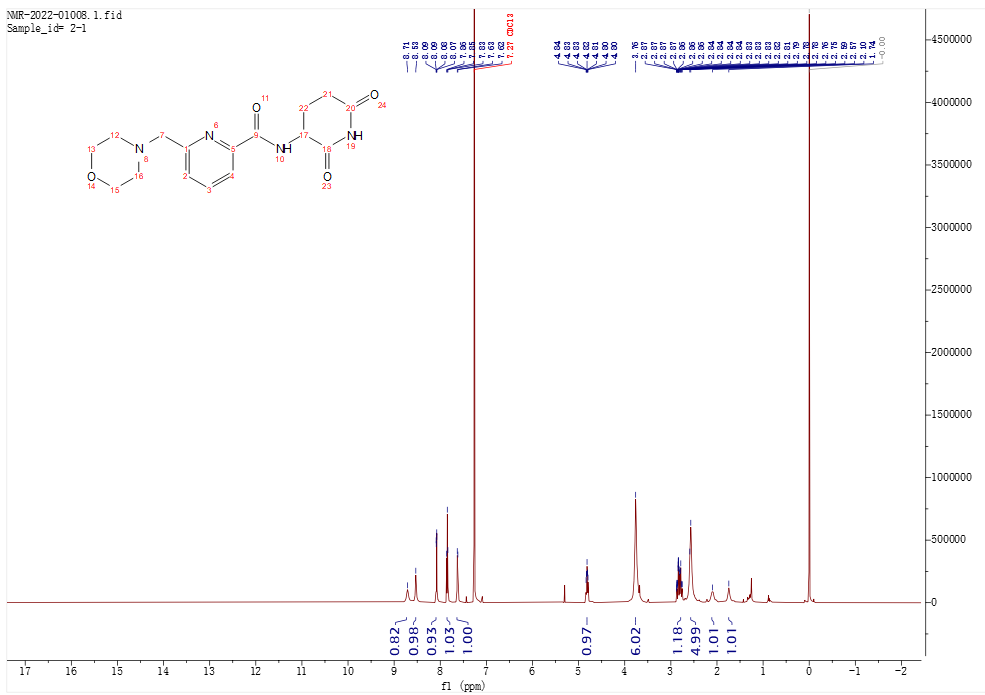


^1^H-NMR spectra of **MGD-B4**


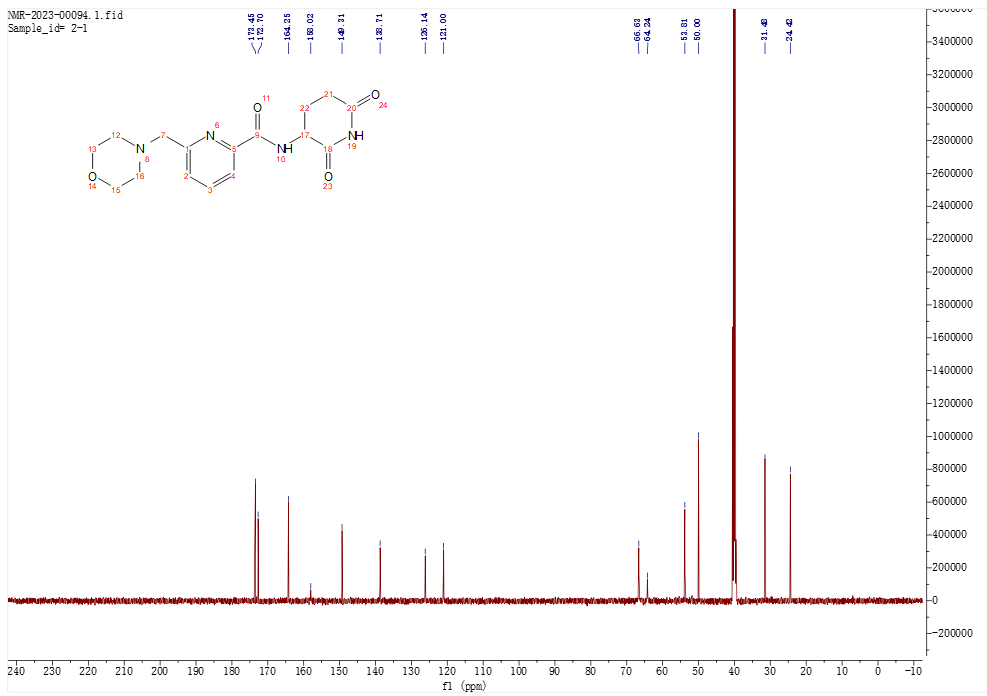


^13^C-NMR spectra of **MGD-B4**


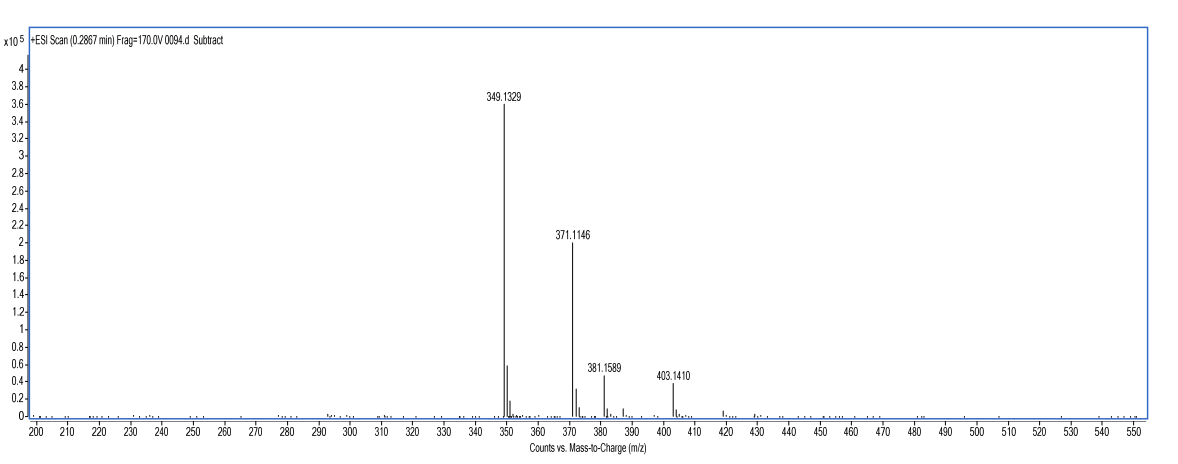


ESI-MS spectra of **MGD-B5**


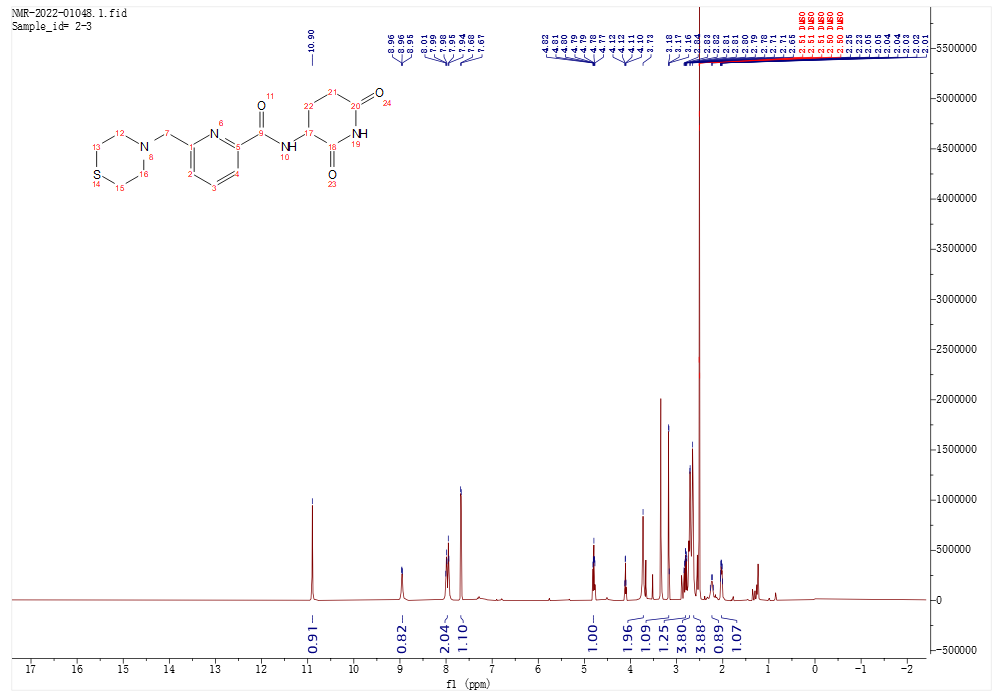


^1^H-NMR spectra of **MGD-B5**


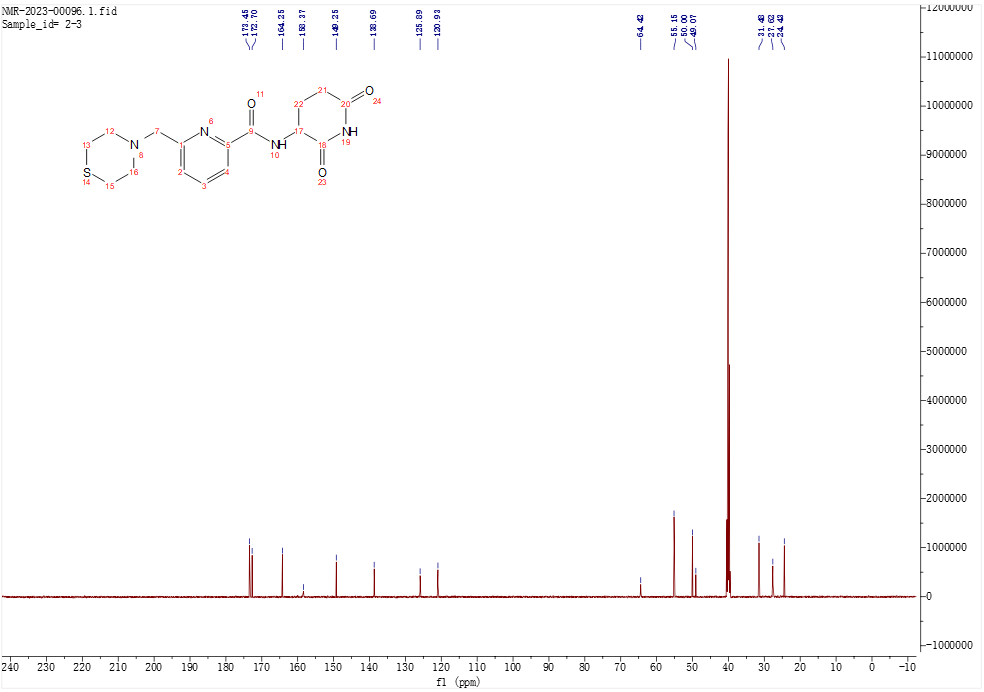


^13^C-NMR spectra of **MGD-B5**


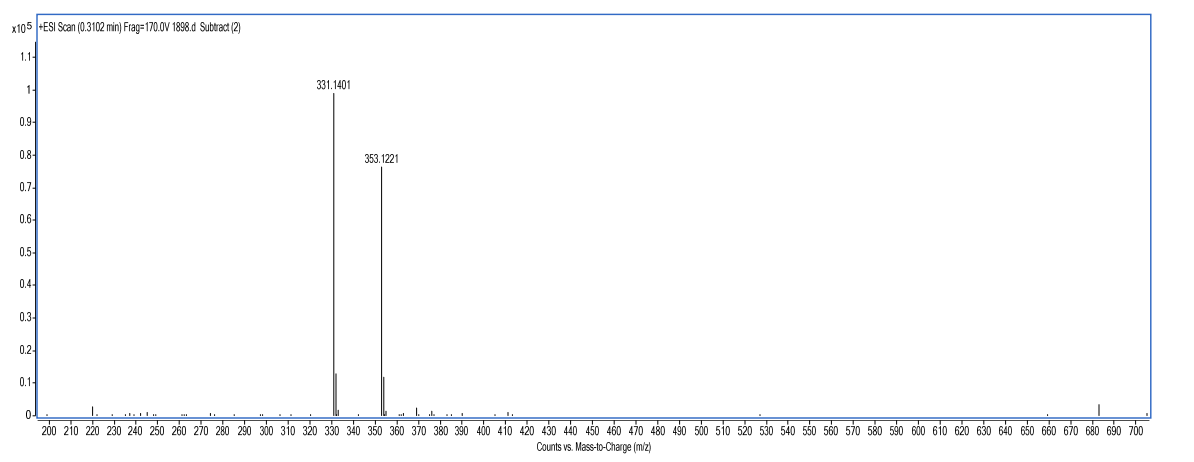


ESI-MS spectra of **MGD-B6**


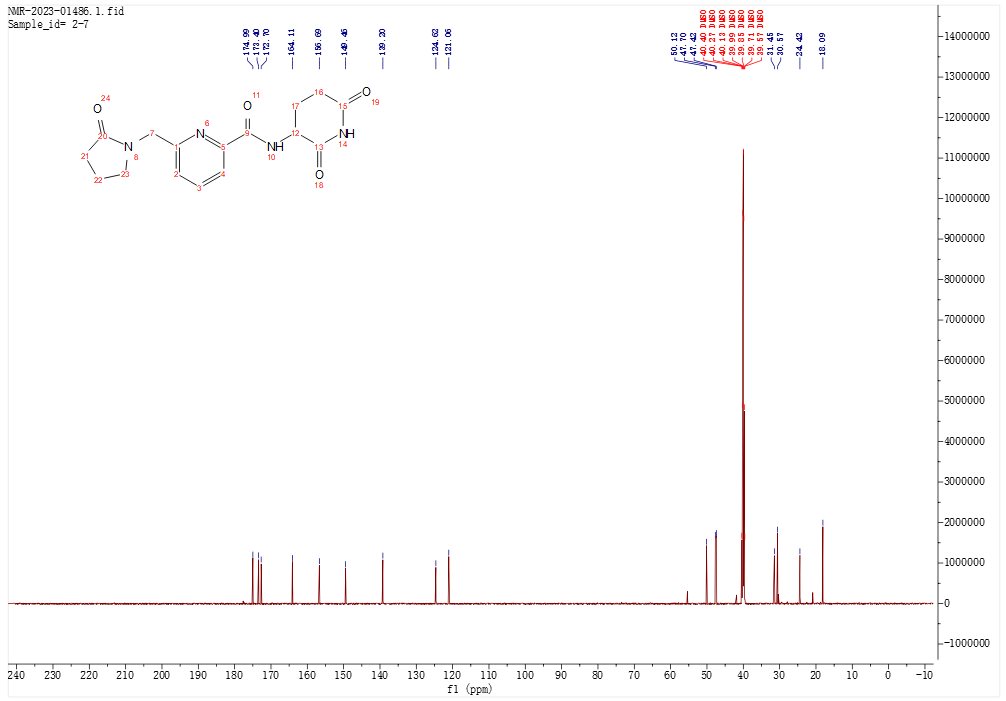


^13^C-NMR spectra of **MGD-B6**


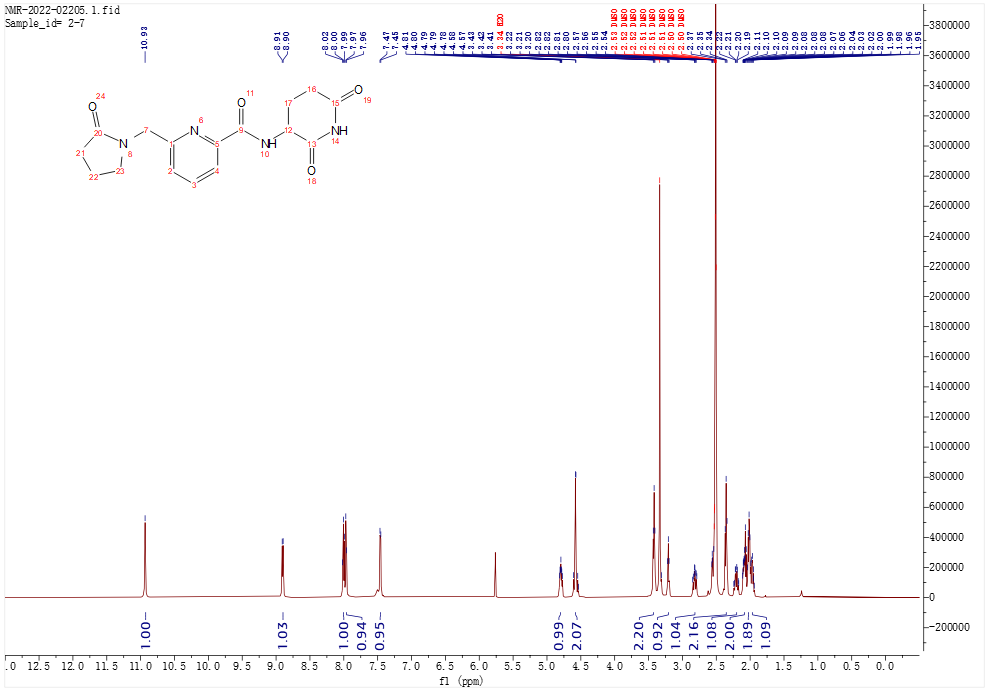


^1^H-NMR spectra of **MGD-B6**


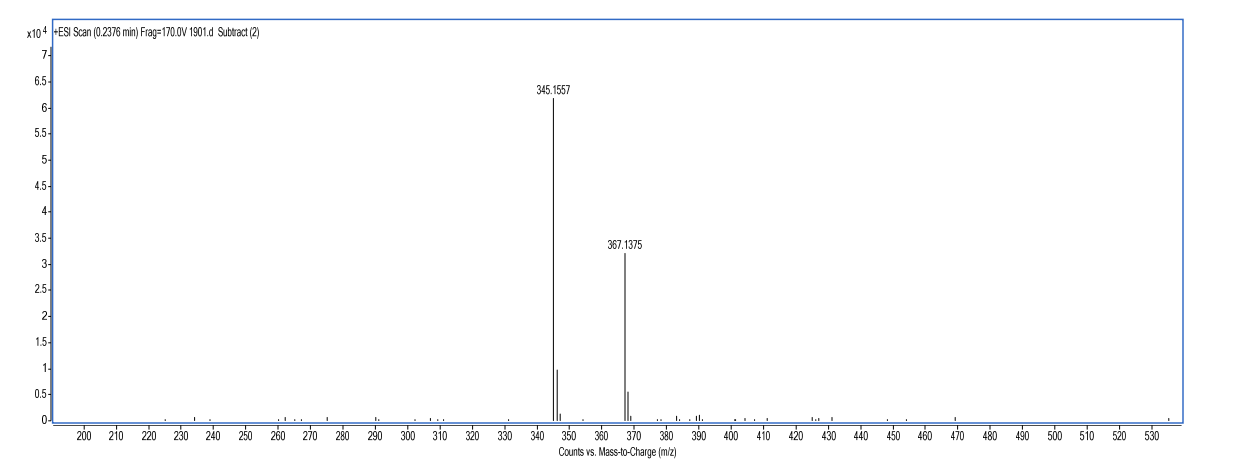


ESI-MS spectra of **MGD-B7**


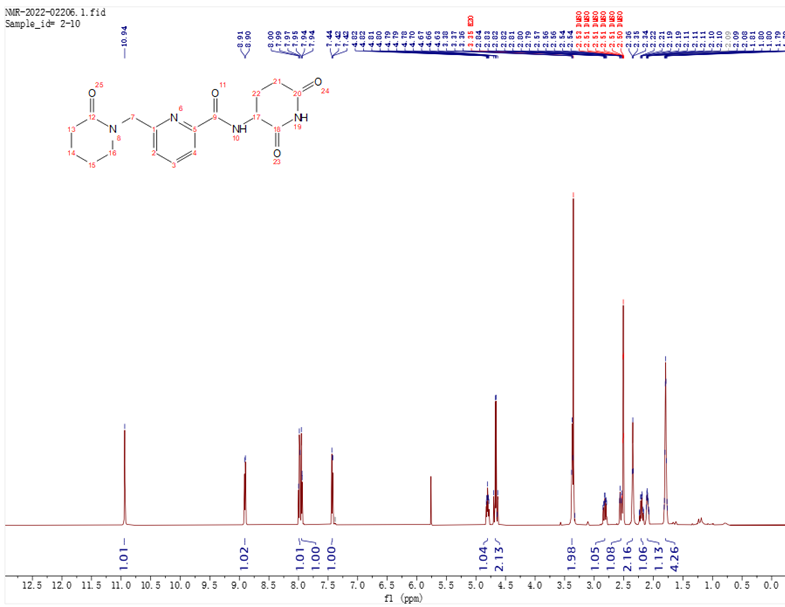


^1^H-NMR spectra of **MGD-B7**


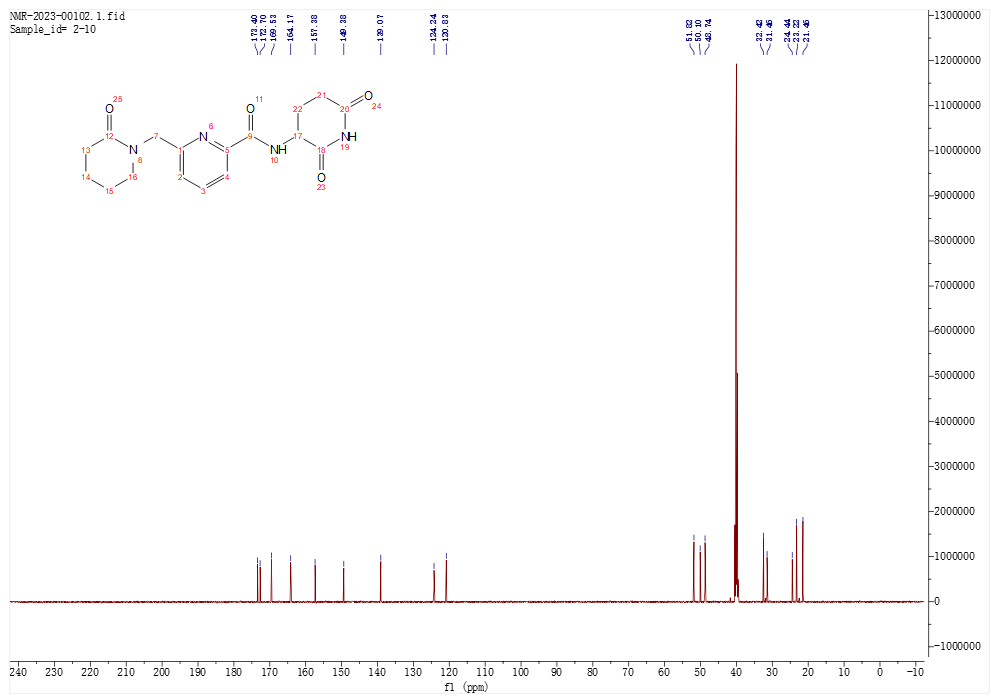


^13^C-NMR spectra of **MGD-B7**


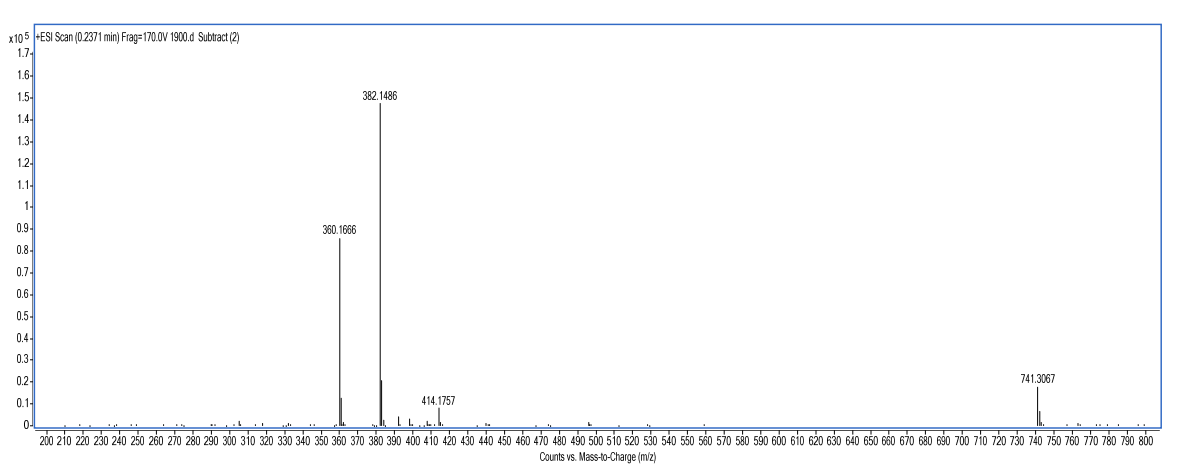


ESI-MS spectra of **MGD-B8**


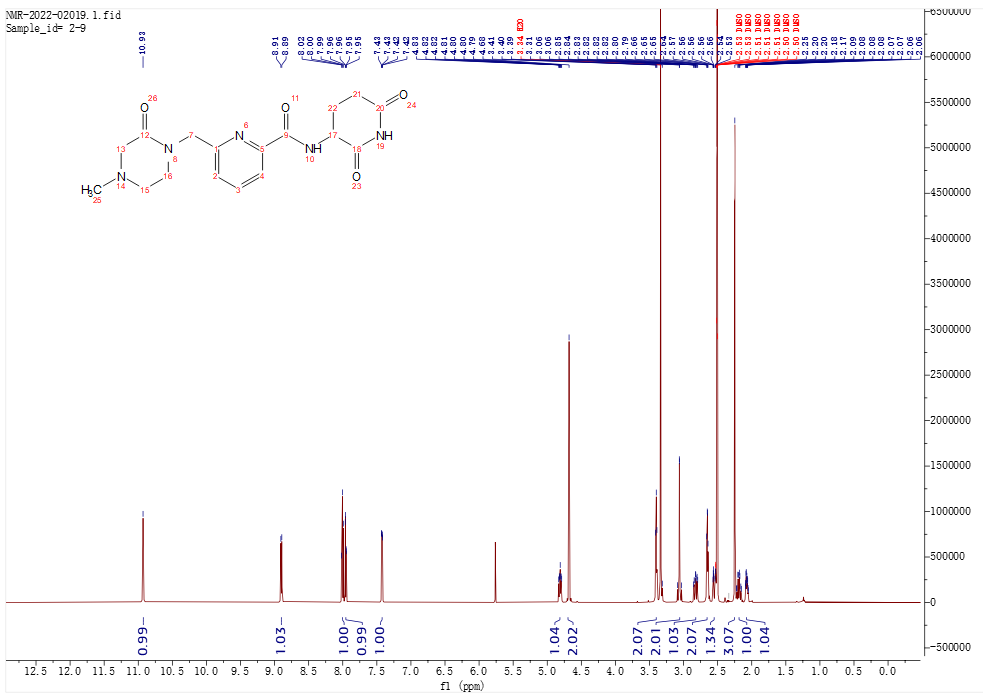


^1^H-NMR spectra of **MGD-B8**


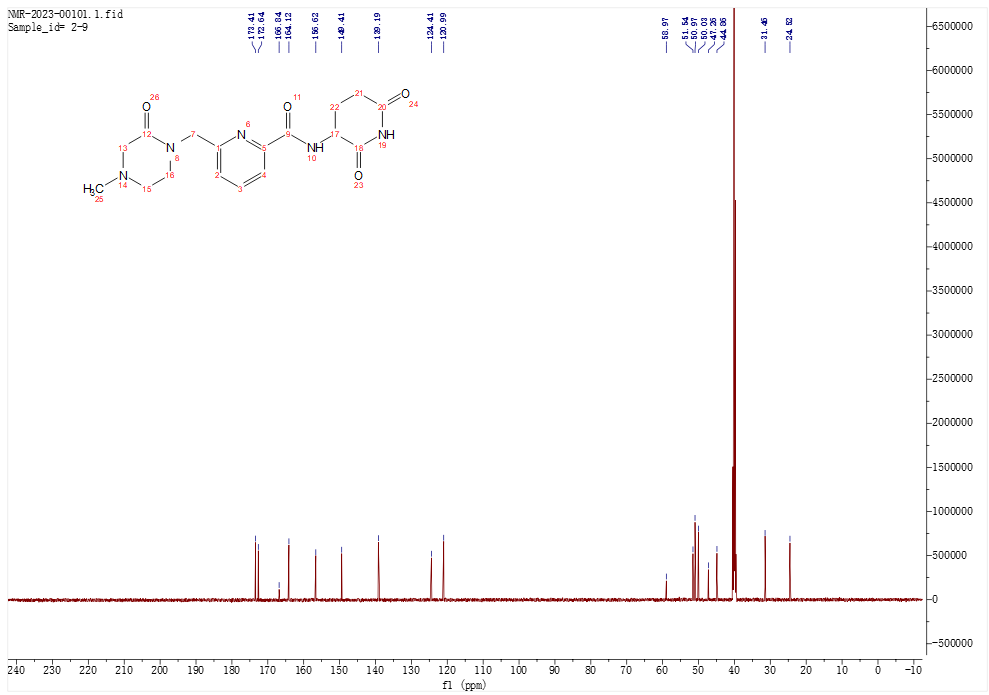


^13^C-NMR spectra of **MGD-B8**


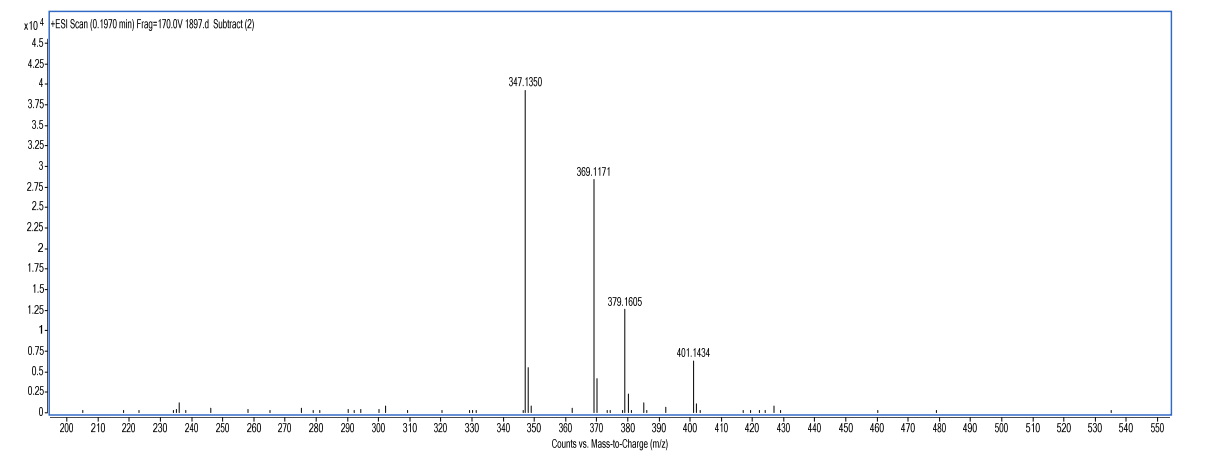


ESI-MS spectra of **MGD-B9**


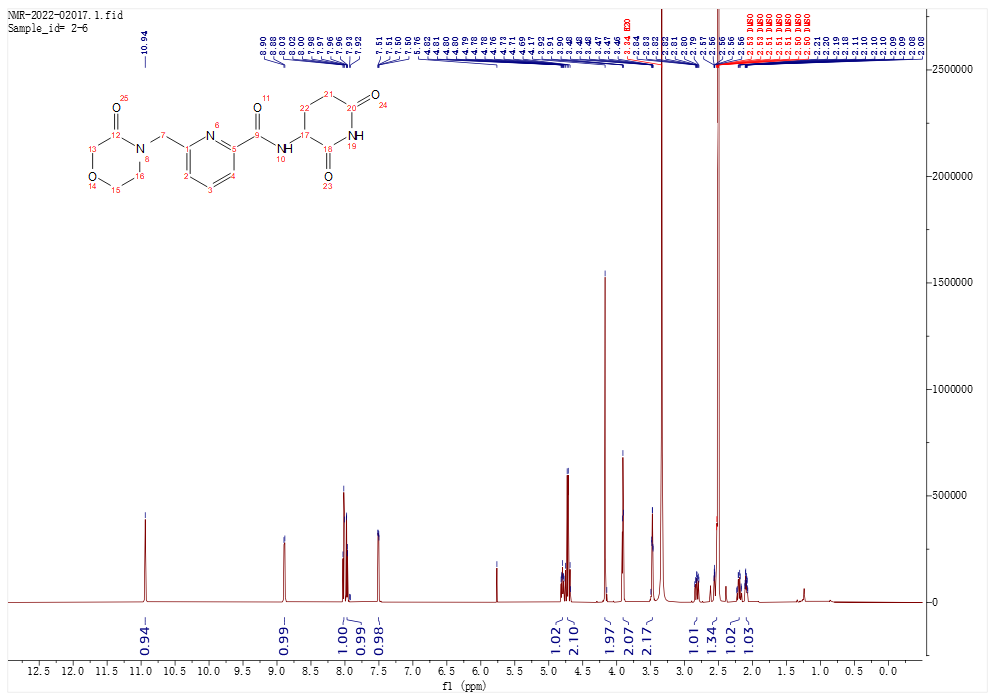


^1^H-NMR spectra of **MGD-B9**


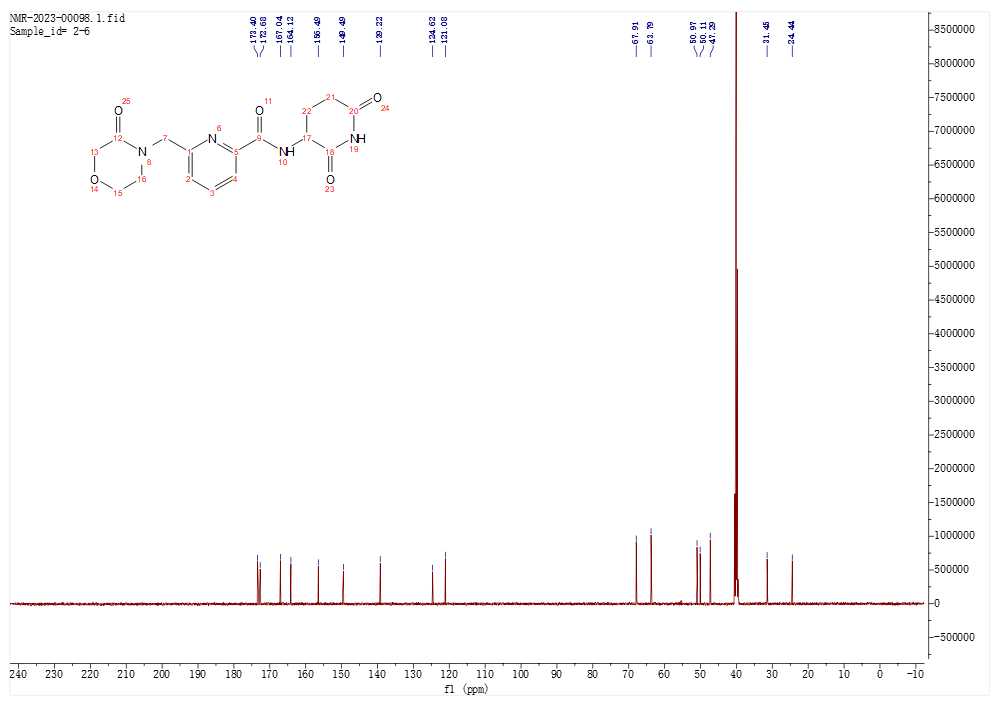


^13^C-NMR spectra of **MGD-B9**


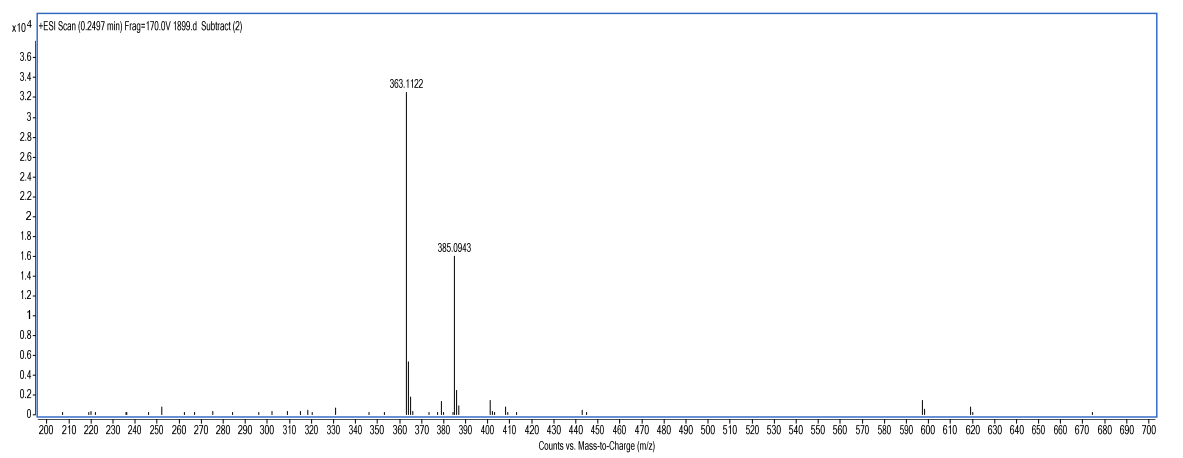


ESI-MS spectra of **MGD-B10**


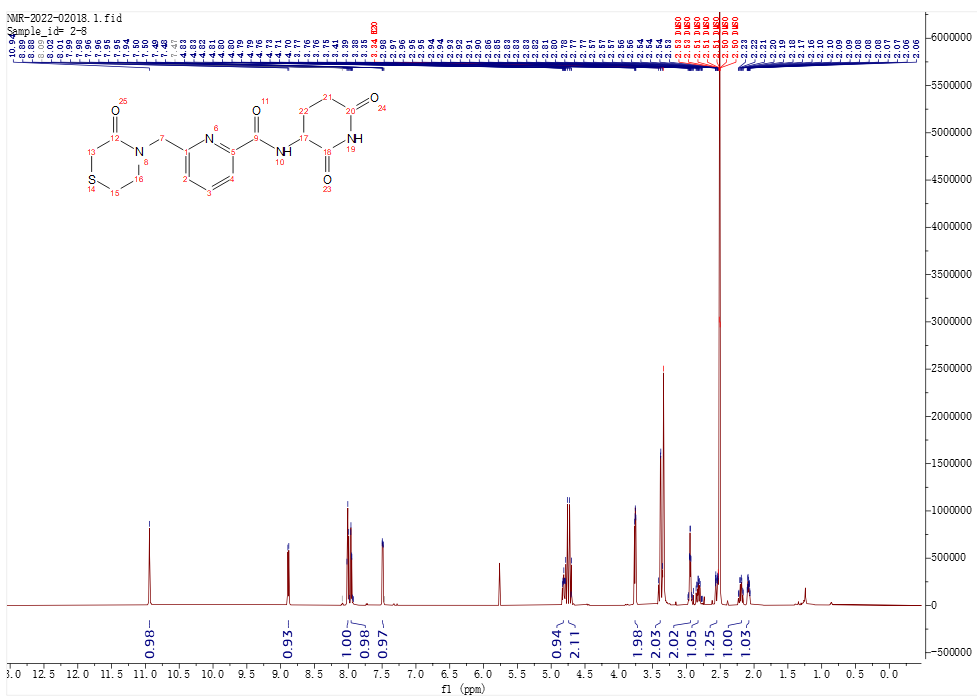


^1^H-NMR spectra of **MGD-B10**


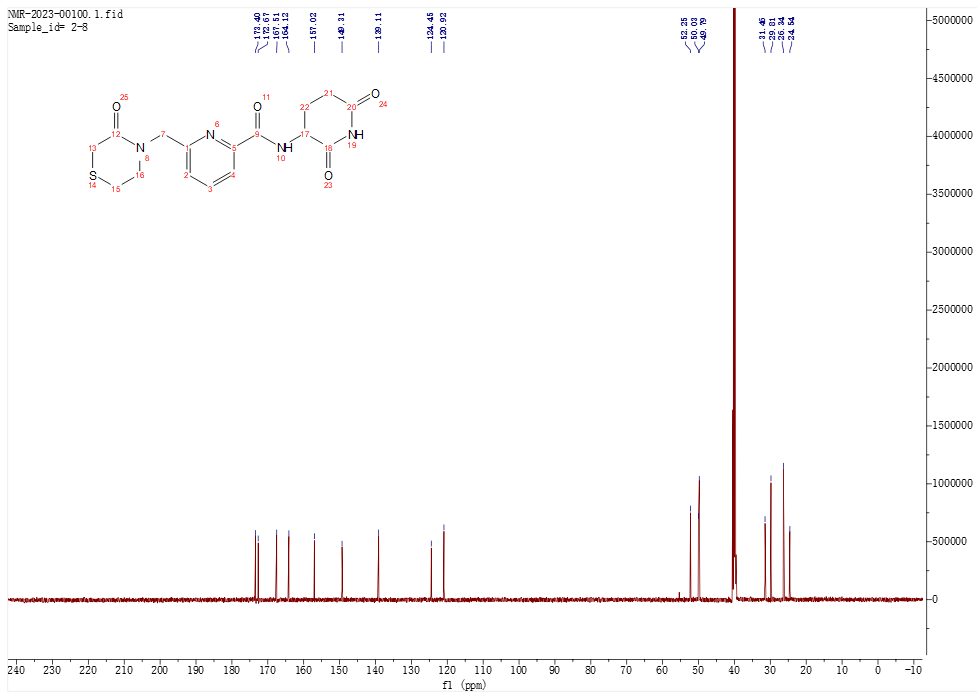


^13^C-NMR spectra of **MGD-B10**


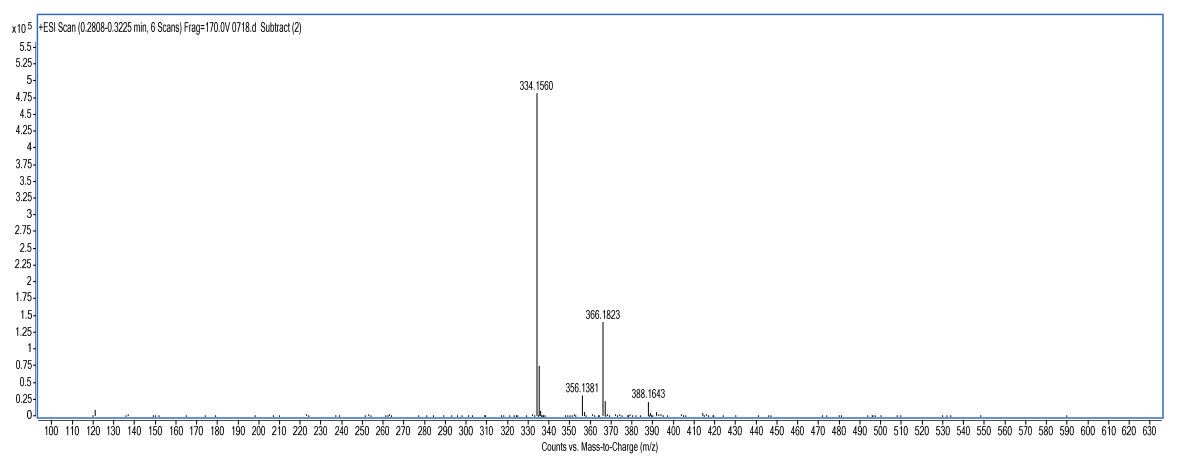


ESI-MS spectra of **MGD-C1**


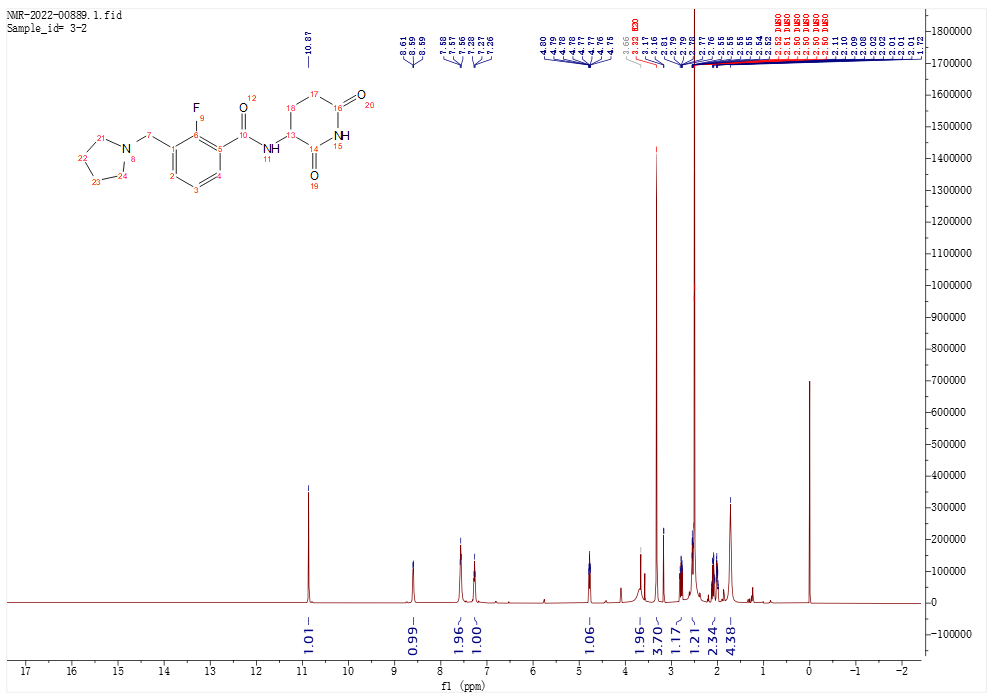


^1^H-NMR spectra of **MGD-C1**


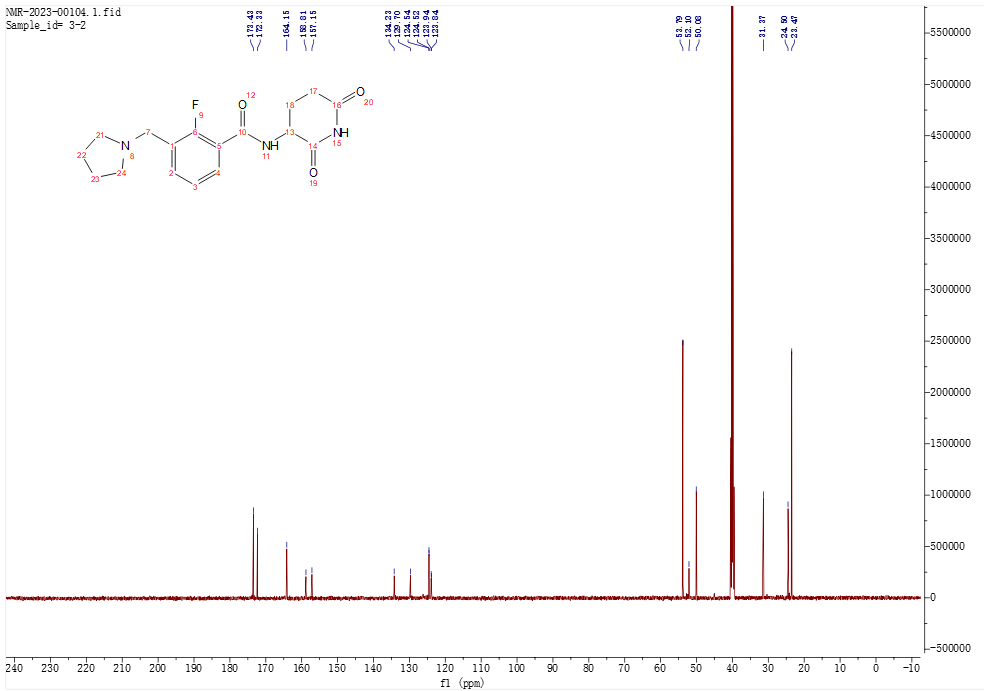


^13^C-NMR spectra of **MGD-C1**


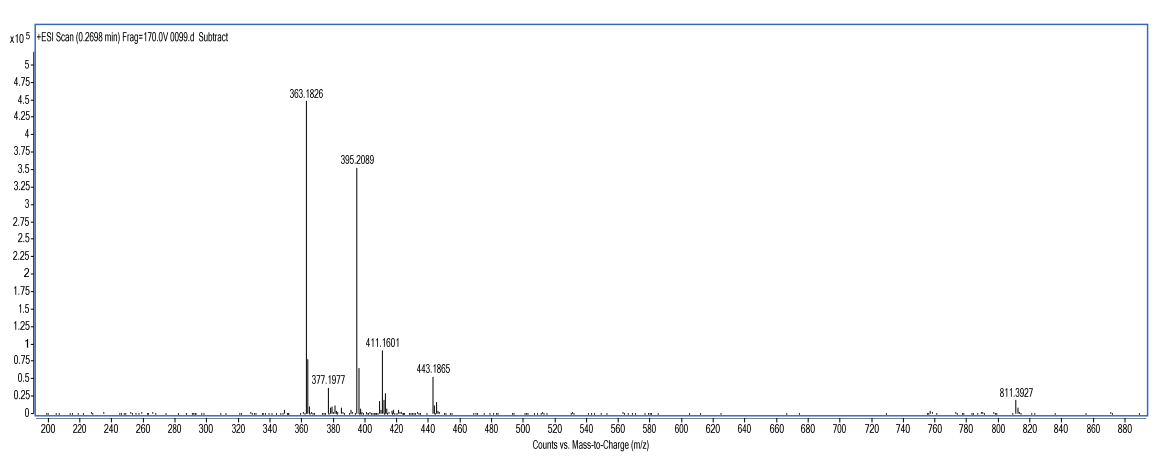


ESI-MS spectra of **MGD-C2**


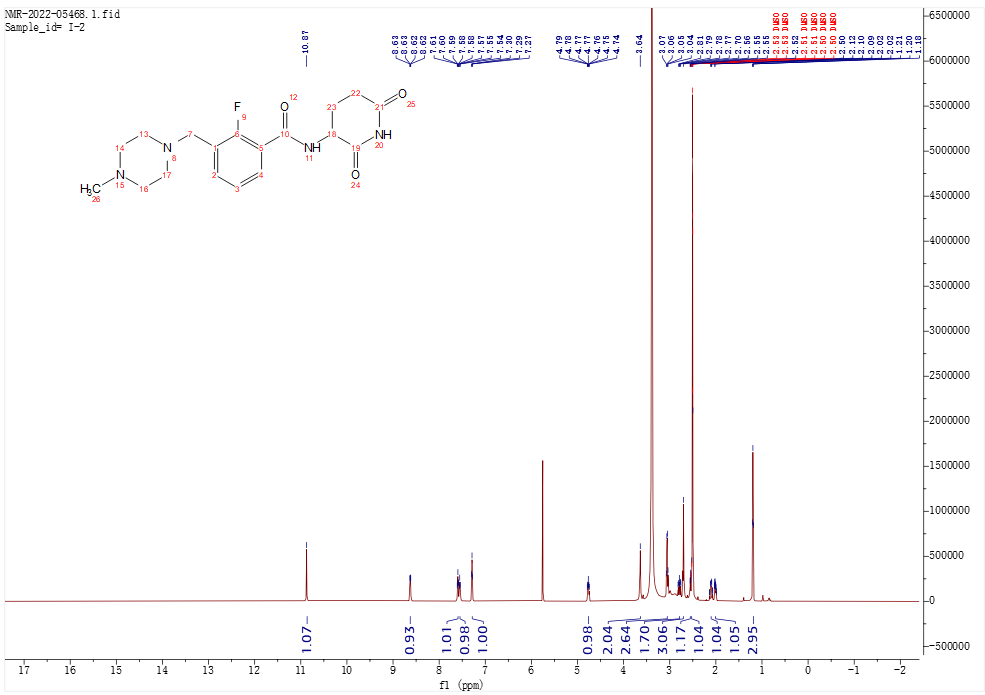


^1^H-NMR spectra of **MGD-C2**


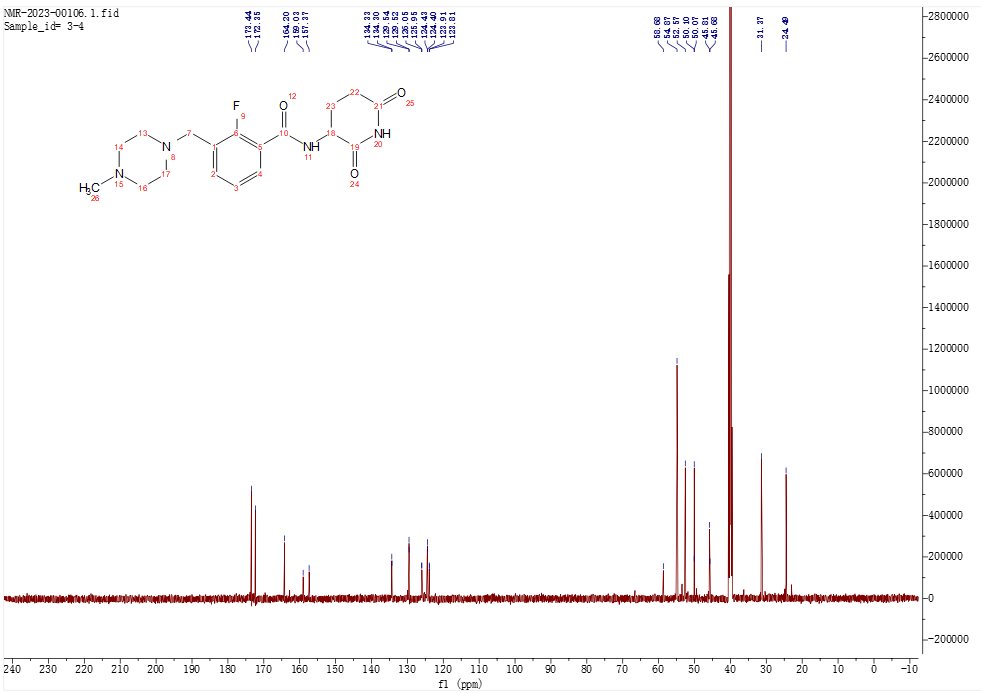


^13^C-NMR spectra of **MGD-C2**


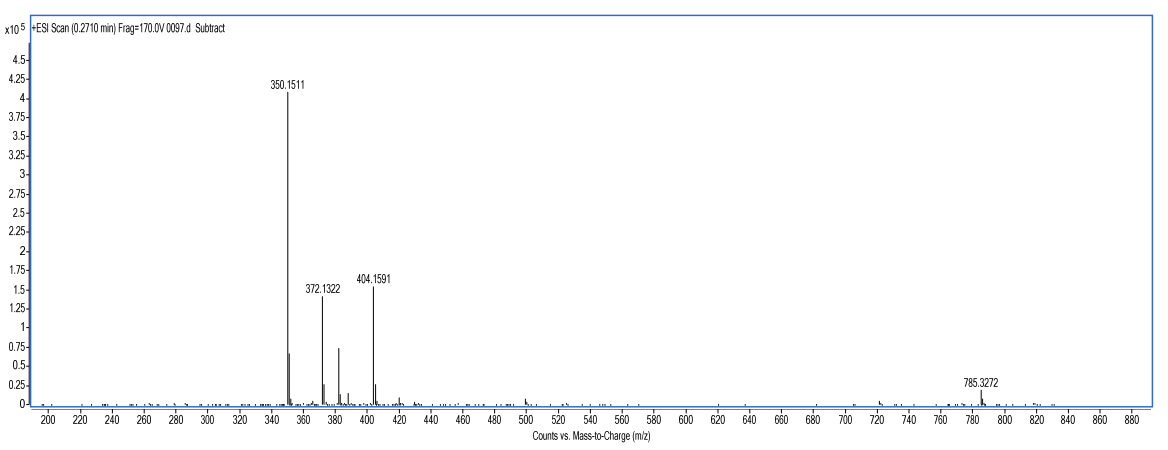


ESI-MS spectra of **MGD-C3**


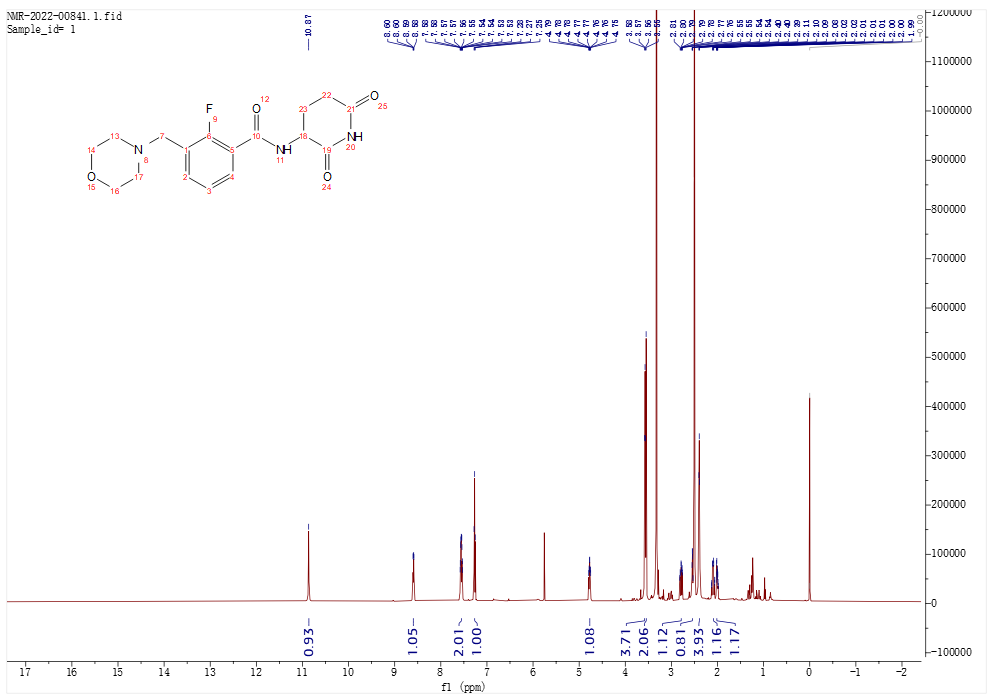


^1^H-NMR spectra of **MGD-C3**


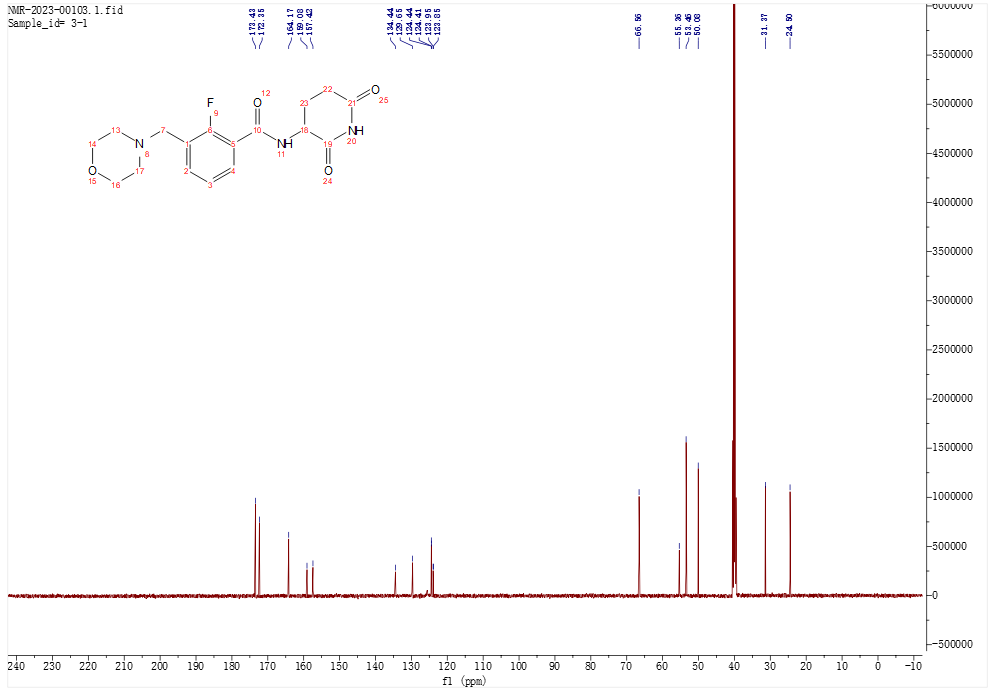


^13^C-NMR spectra of **MGD-C3**


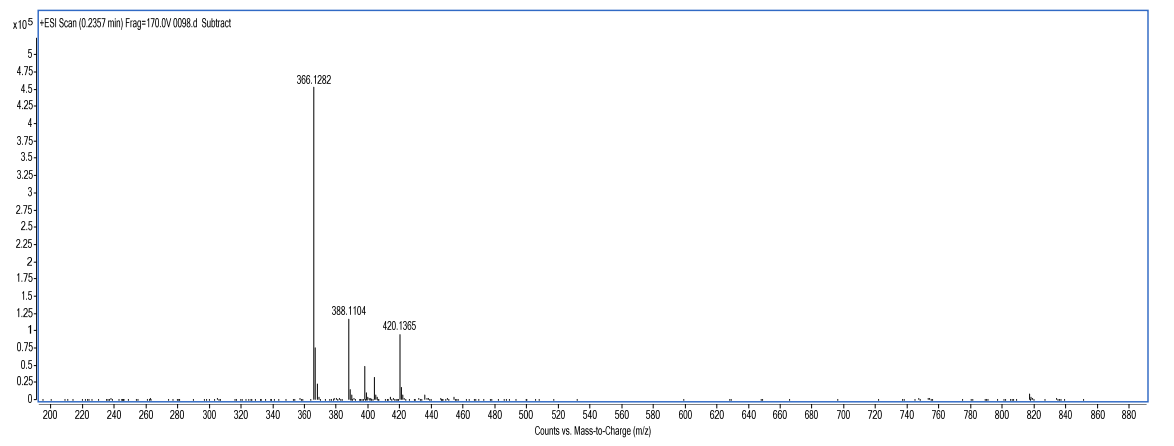


ESI-MS spectra of **MGD-C4**


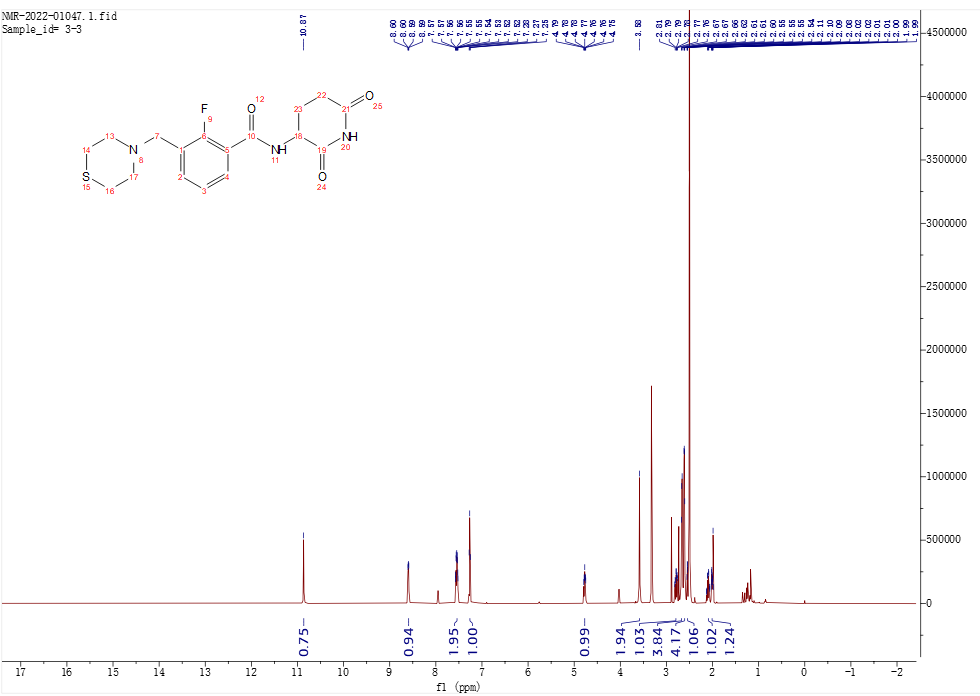


^1^H-NMR spectra of **MGD-C4**


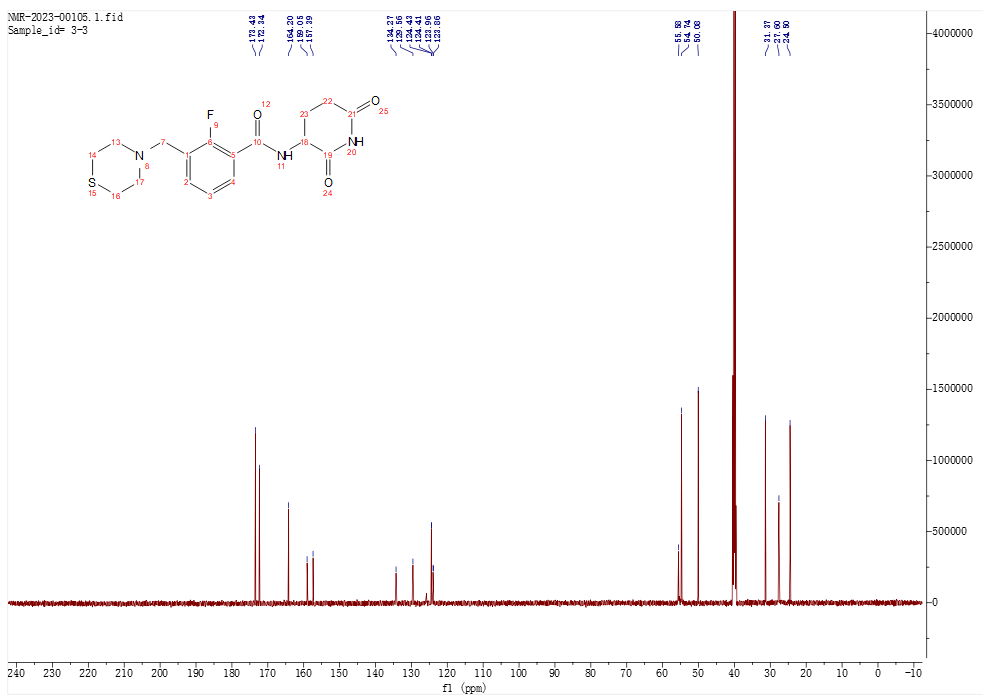


^13^C-NMR spectra of **MGD-C4**


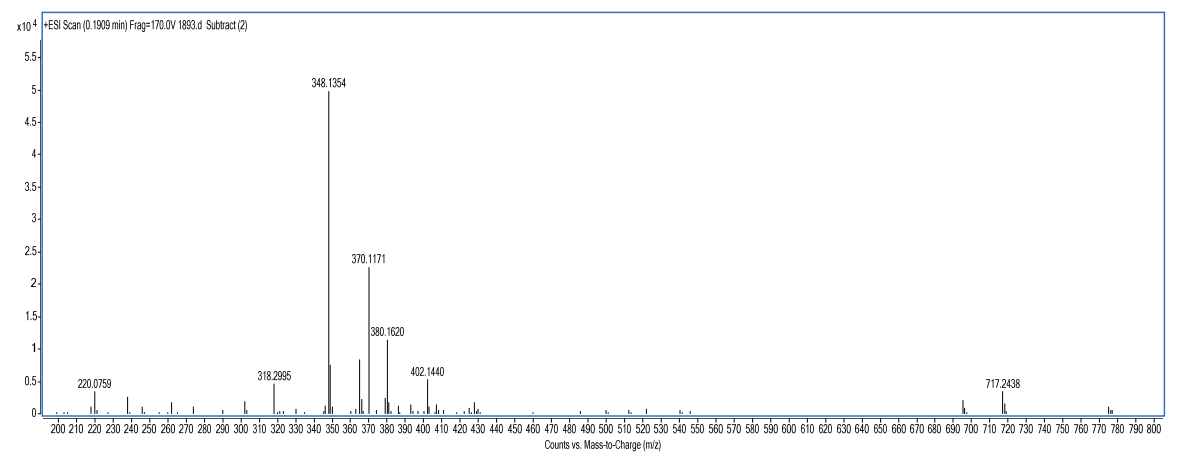


ESI-MS spectra of **MGD-C5**


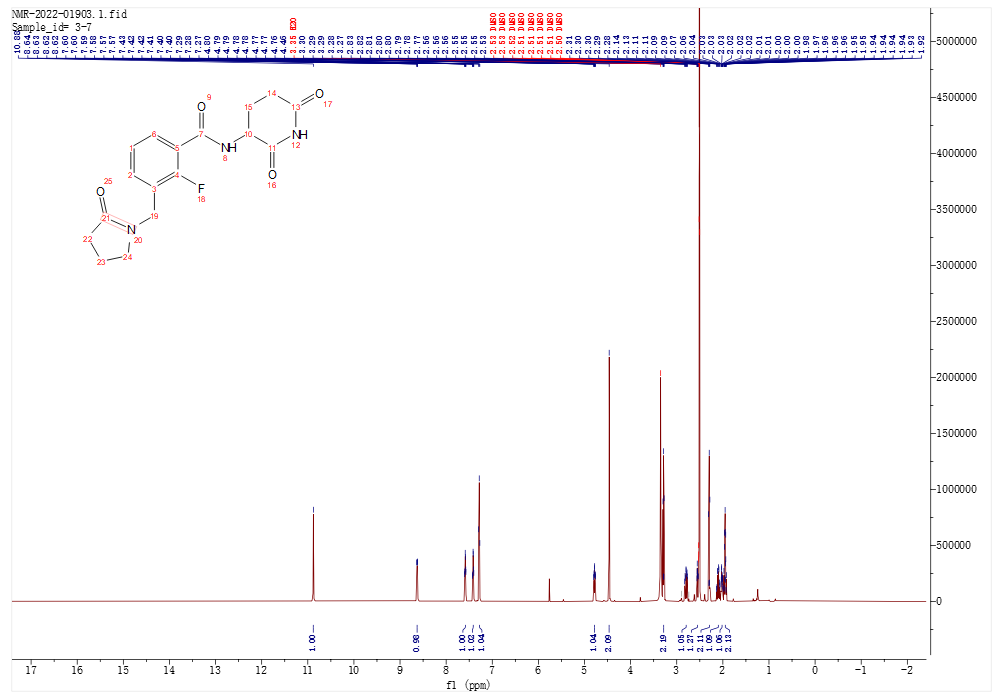


^1^H-NMR spectra of **MGD-C5**


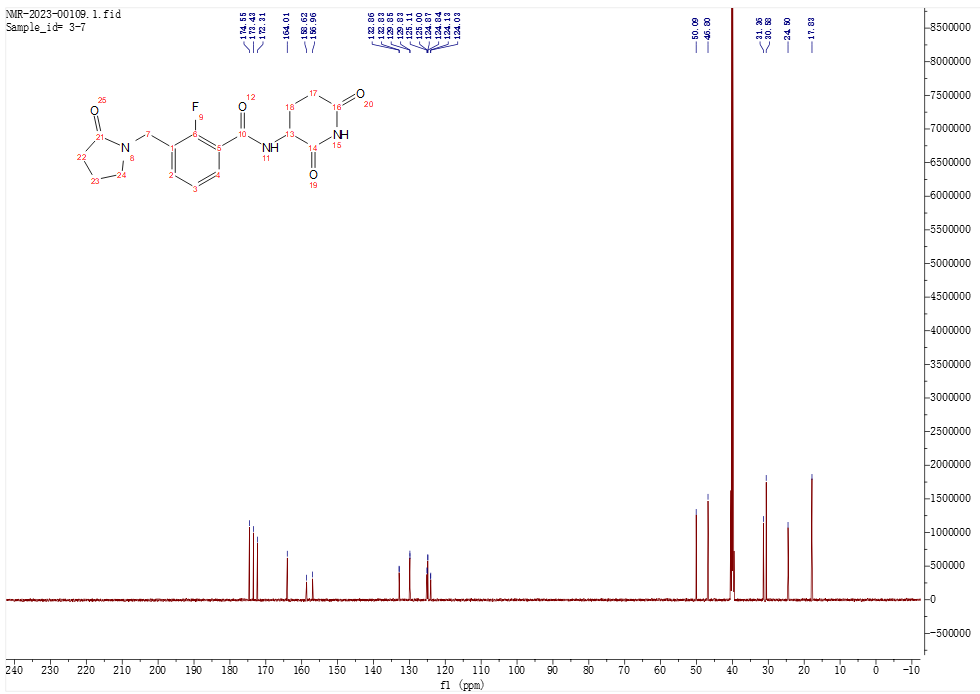


^13^C-NMR spectra of **MGD-C5**


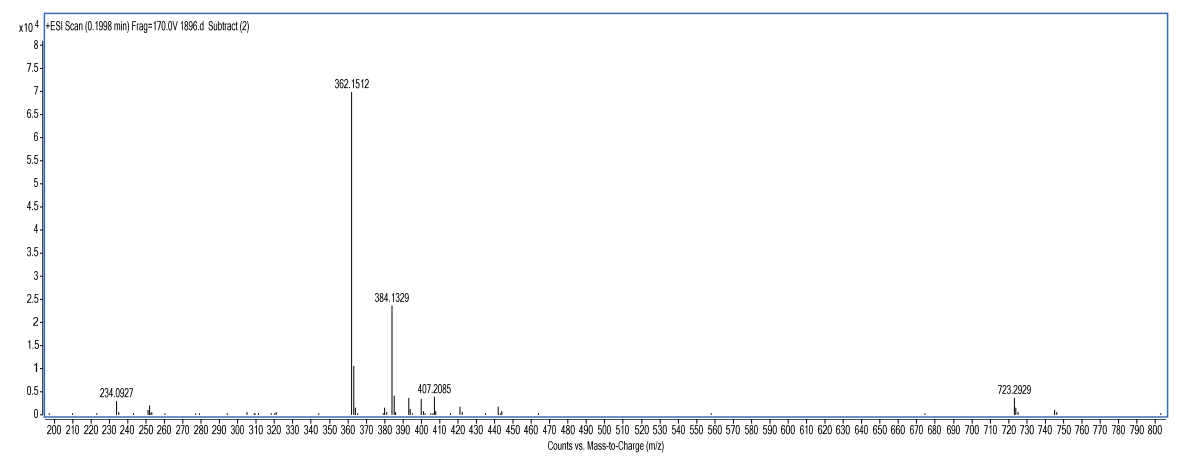


ESI-MS spectra of **MGD-C6**


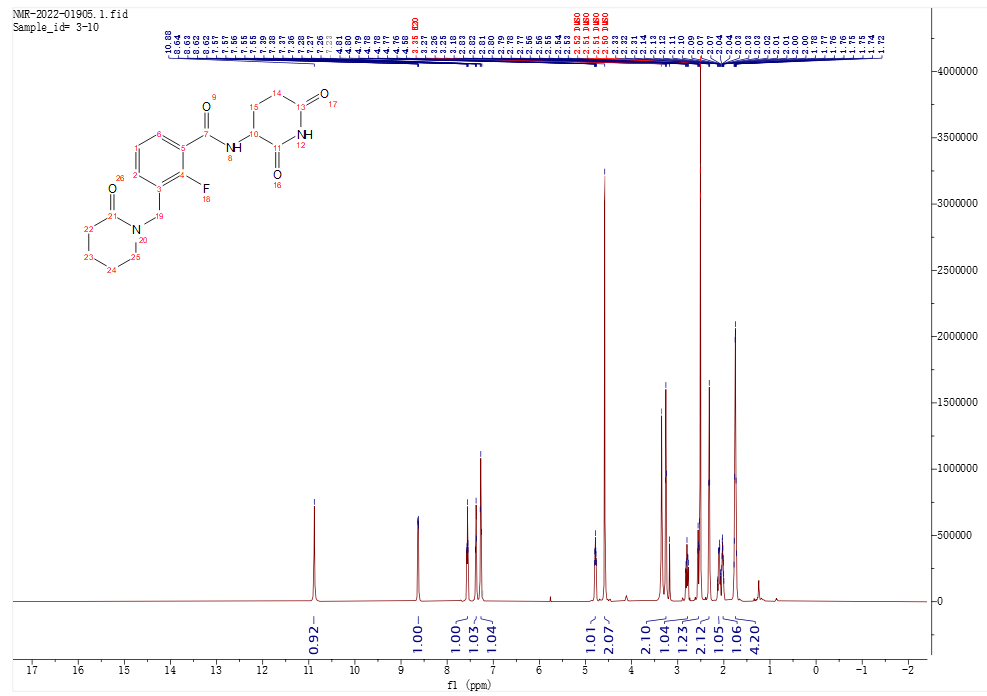


^1^H-NMR spectra of **MGD-C6**


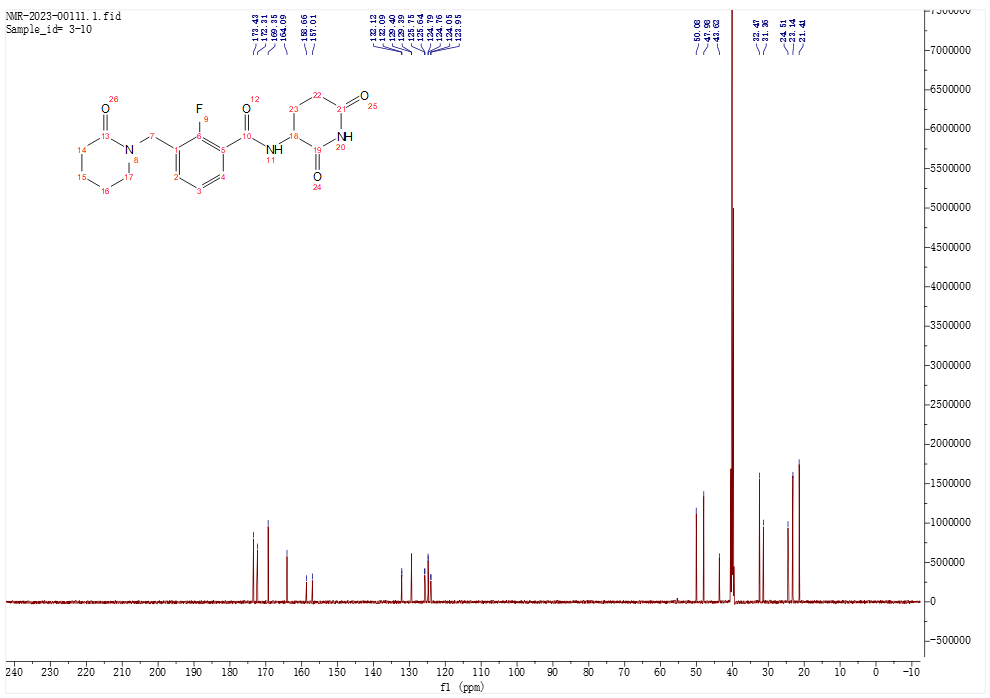


^13^C-NMR spectra of **MGD-C6**


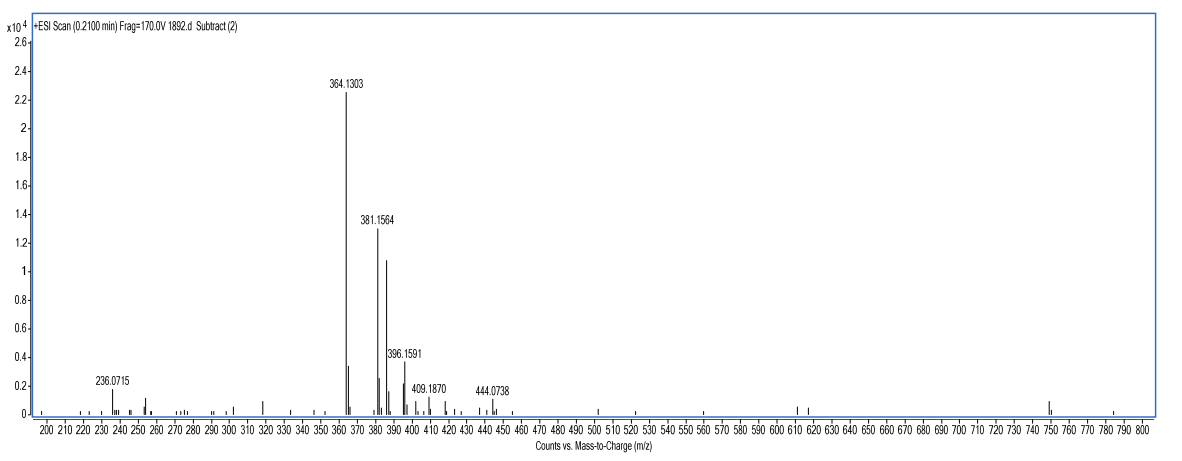


ESI-MS spectra of **MGD-C7**


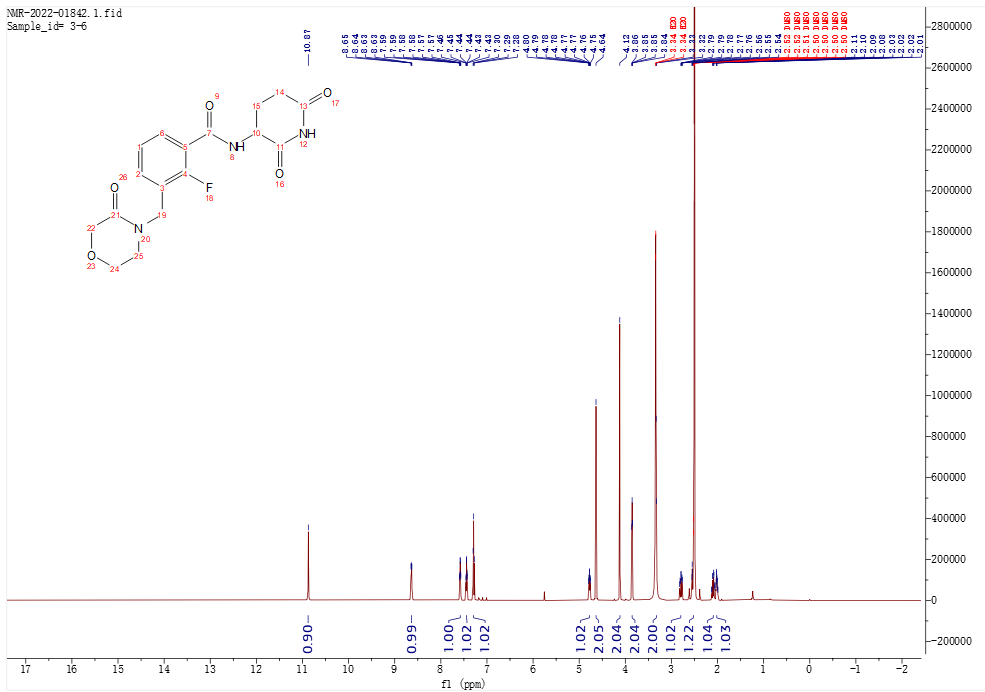


^1^H-NMR spectra of **MGD-C7**


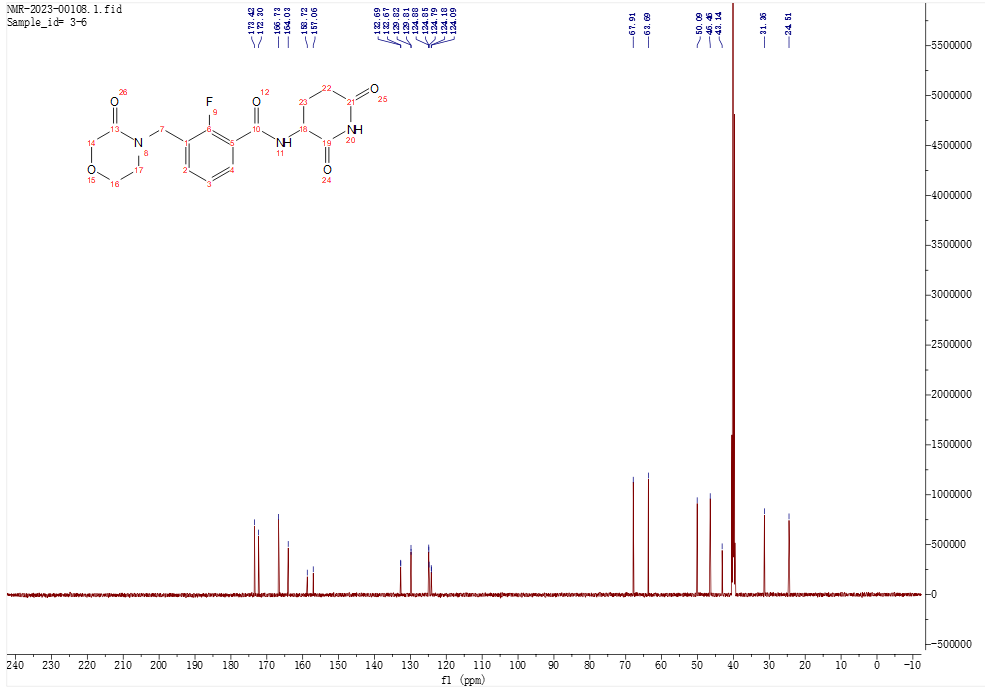


^13^C-NMR spectra of **MGD-C7**


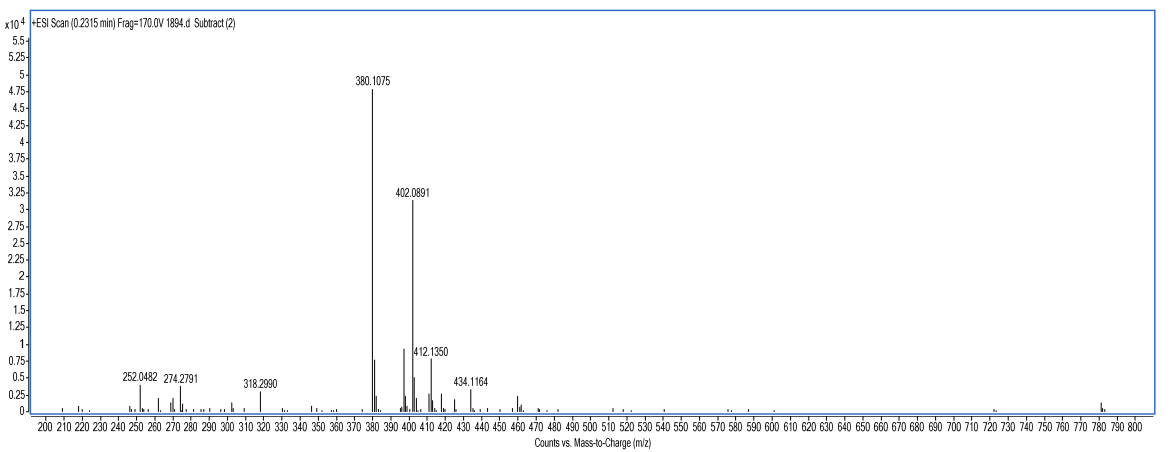


ESI-MS spectra of **MGD-C8**


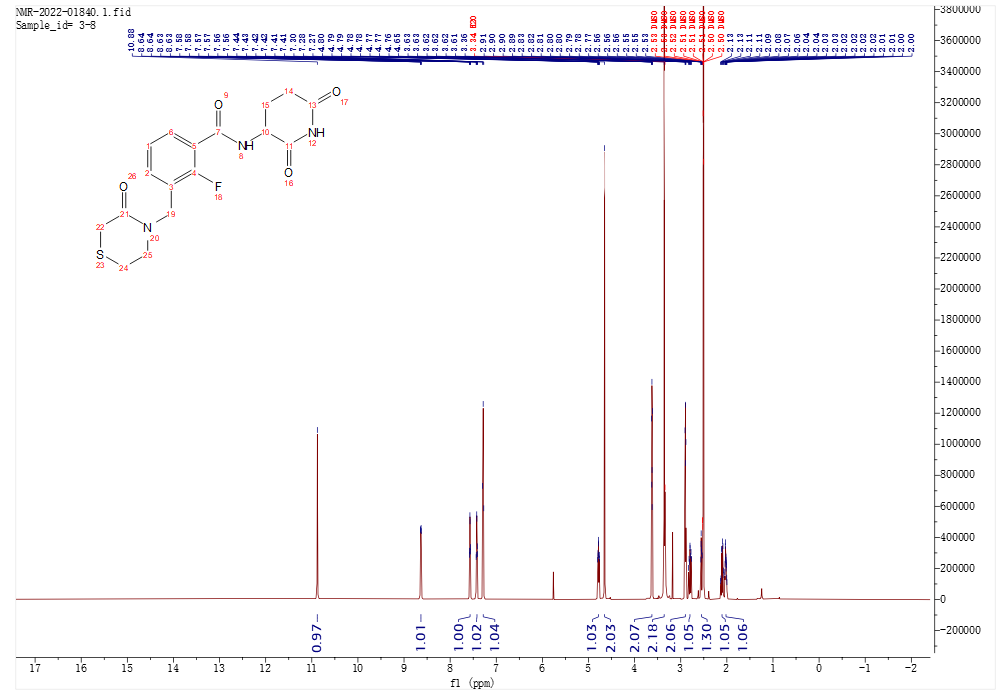


^1^H-NMR spectra of **MGD-C8**


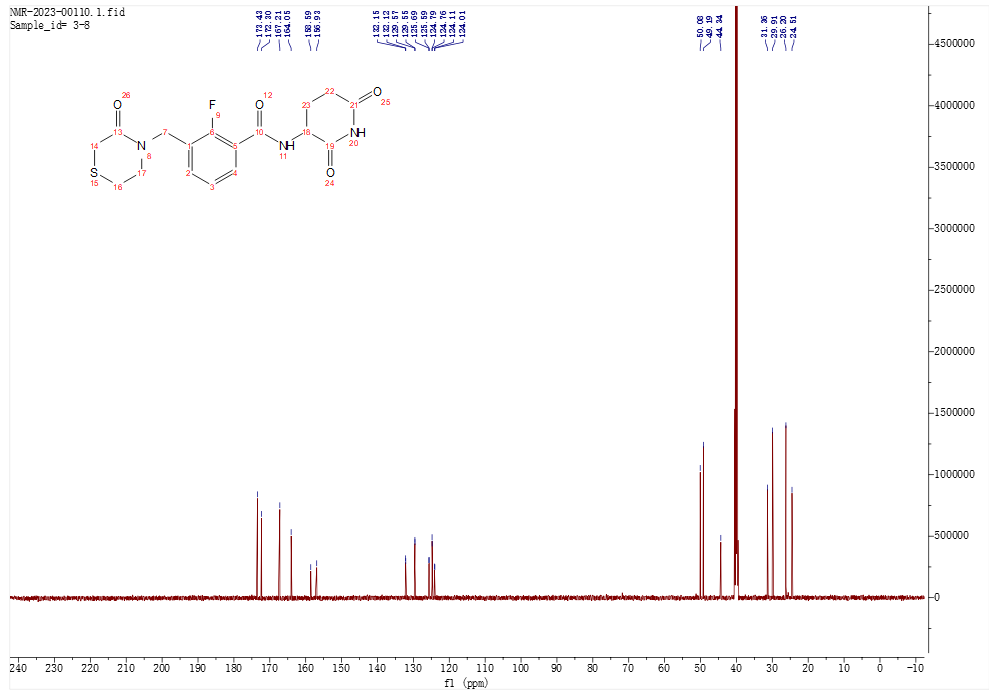


^13^C-NMR spectra of **MGD-C8**


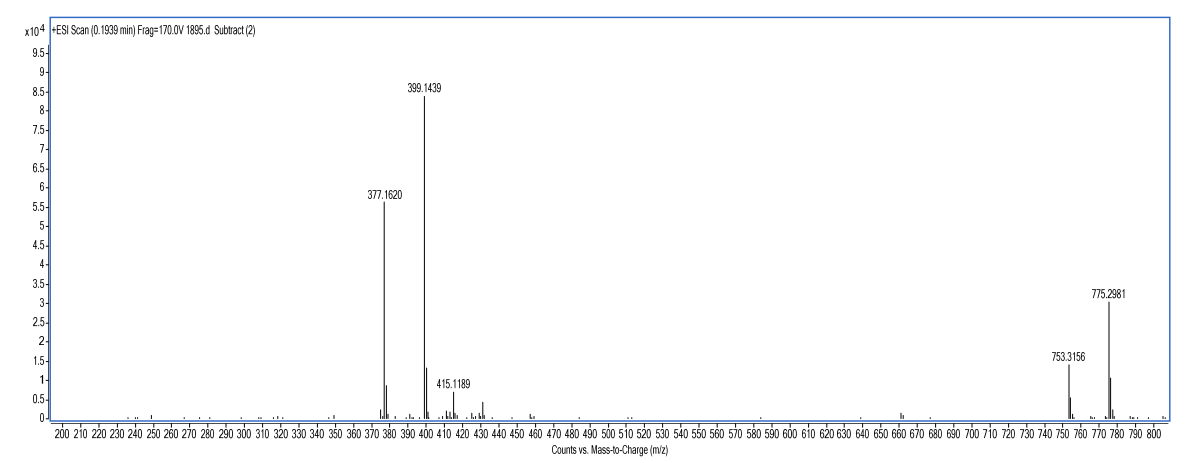


ESI-MS spectra of **MGD-C9**


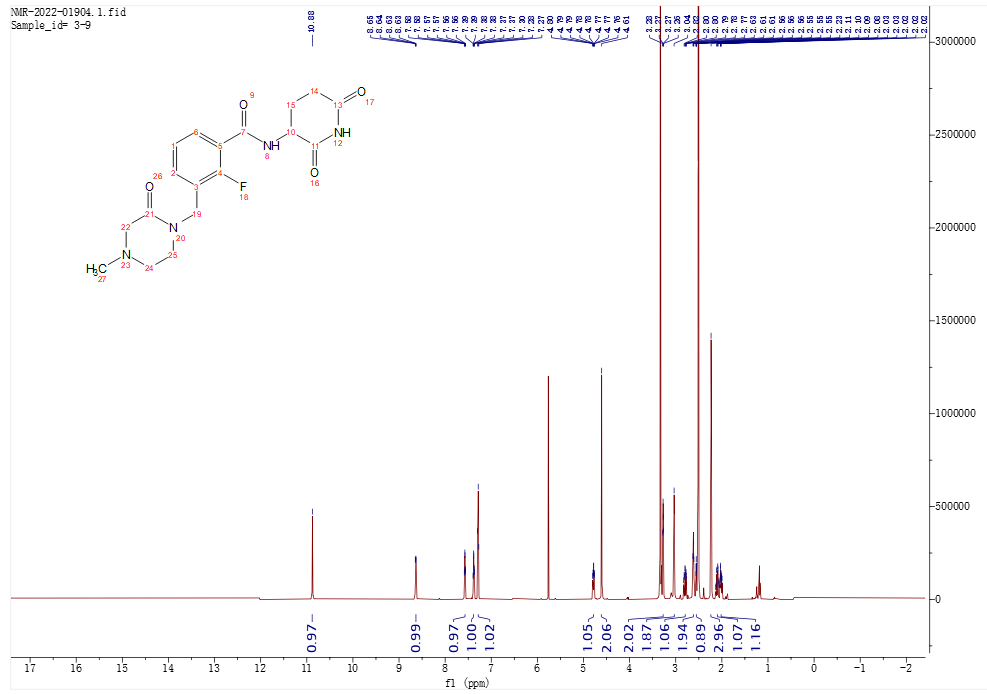


^1^H-NMR spectra of **MGD-C9**


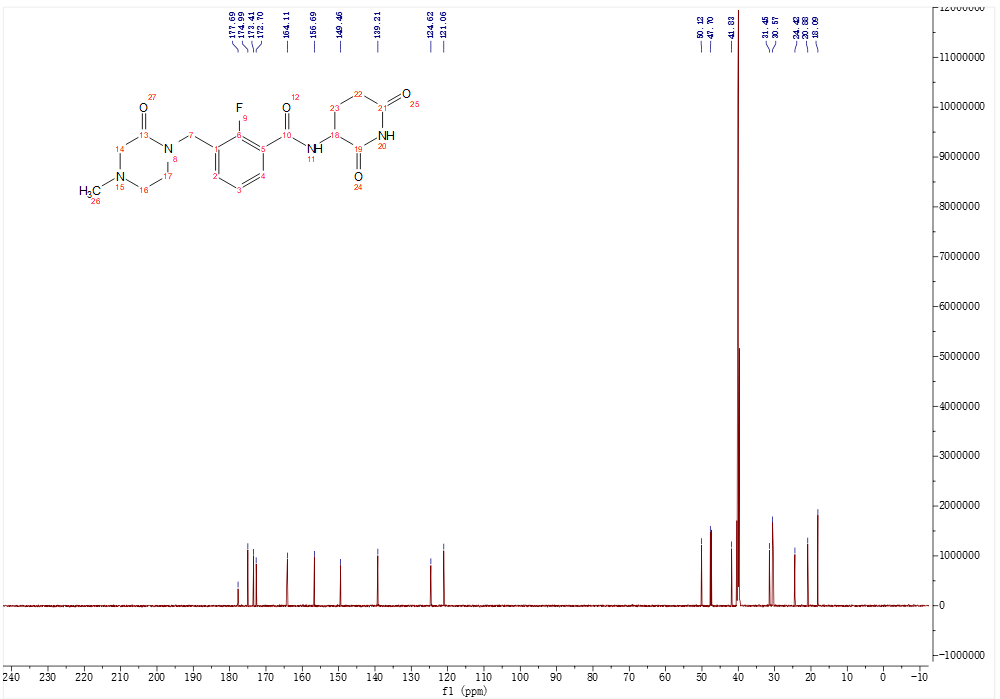


^13^C-NMR spectra of **MGD-C9**
