# Supplementary figures and images for: Discovery and Characterization of Novel IKZF1/3 Glue Degraders against Multiple Hematological Cancer Cell Lines
Source: Oncol Res. 2025 Sep 26;33(10):2981–3006. doi: 10.32604/or.2025.065123 (PMC12494099; doi:10.32604/or.2025.065123)

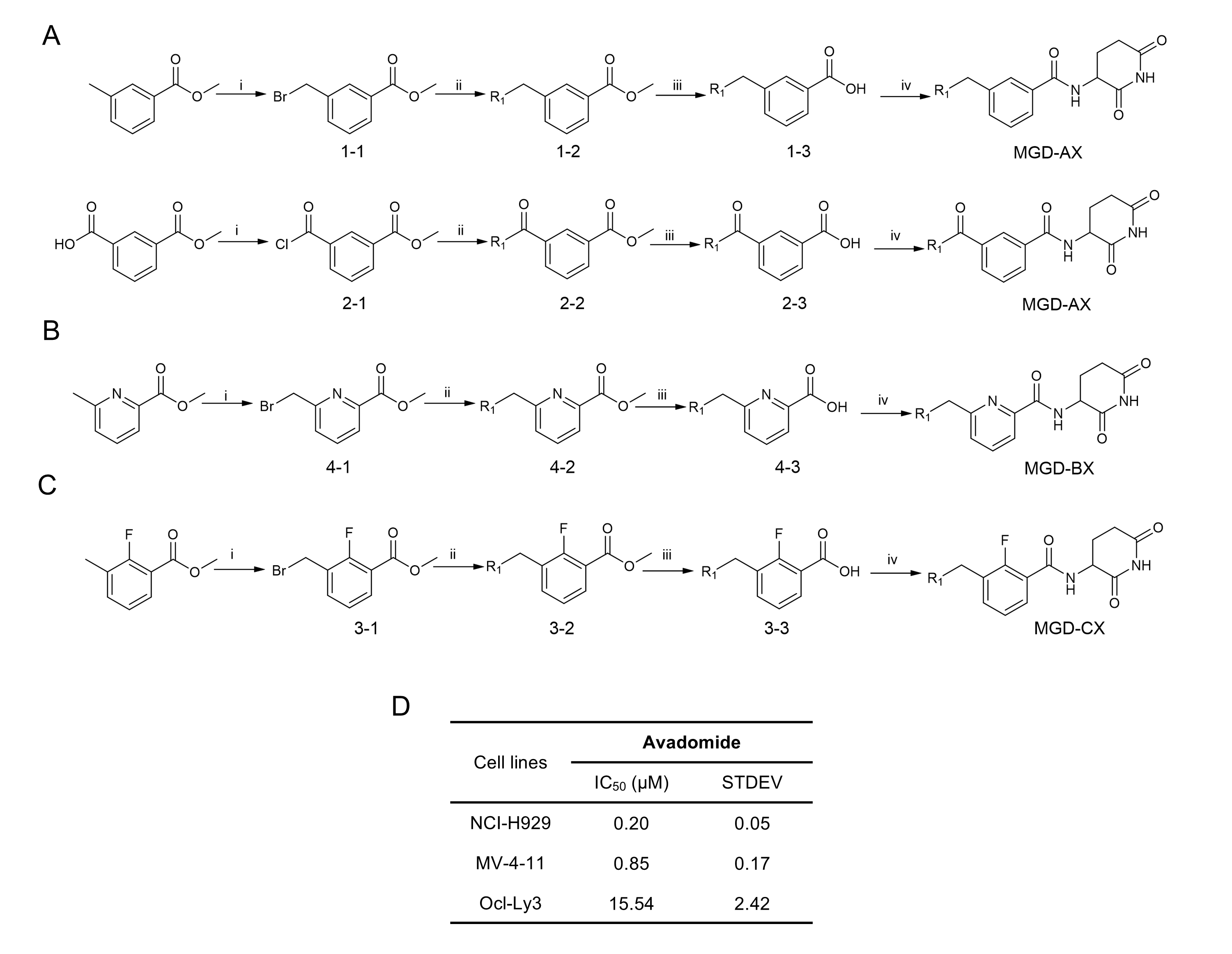

Supplement: Figure S1 [file OncolRes-33-65123-s003.TIF]

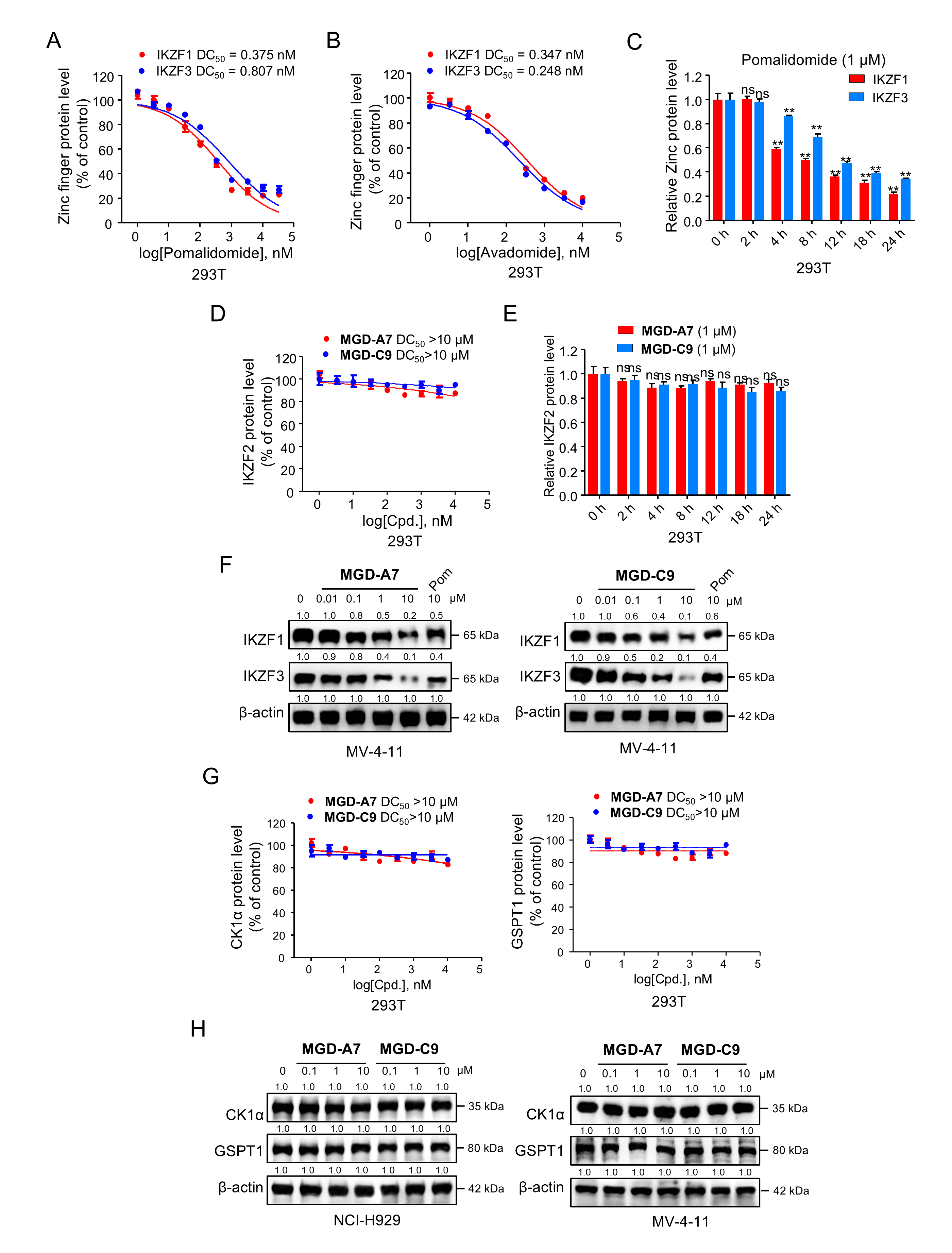

Supplement: Figure S2 [file OncolRes-33-65123-s004.TIF]

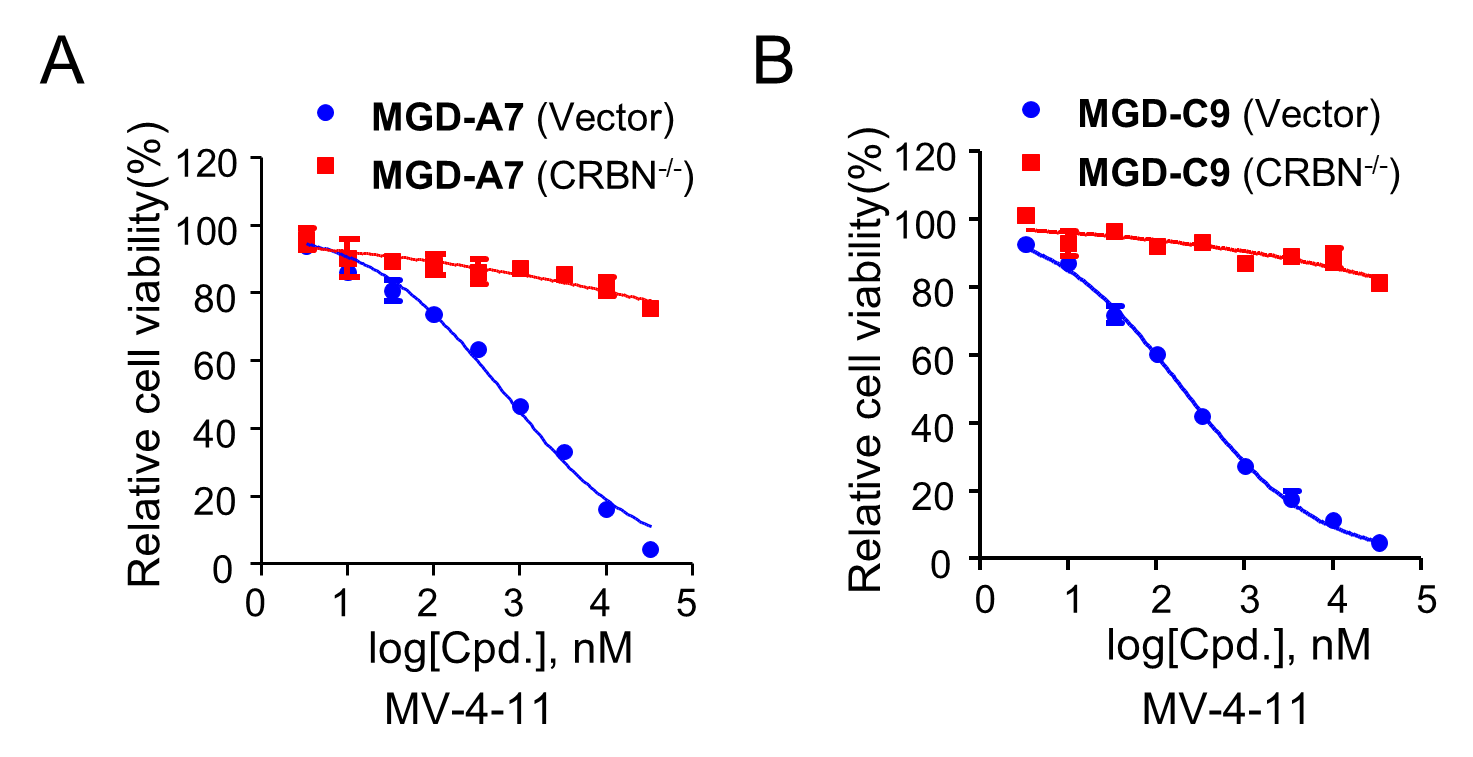

Supplement: Figure S3 [file OncolRes-33-65123-s005.TIF]

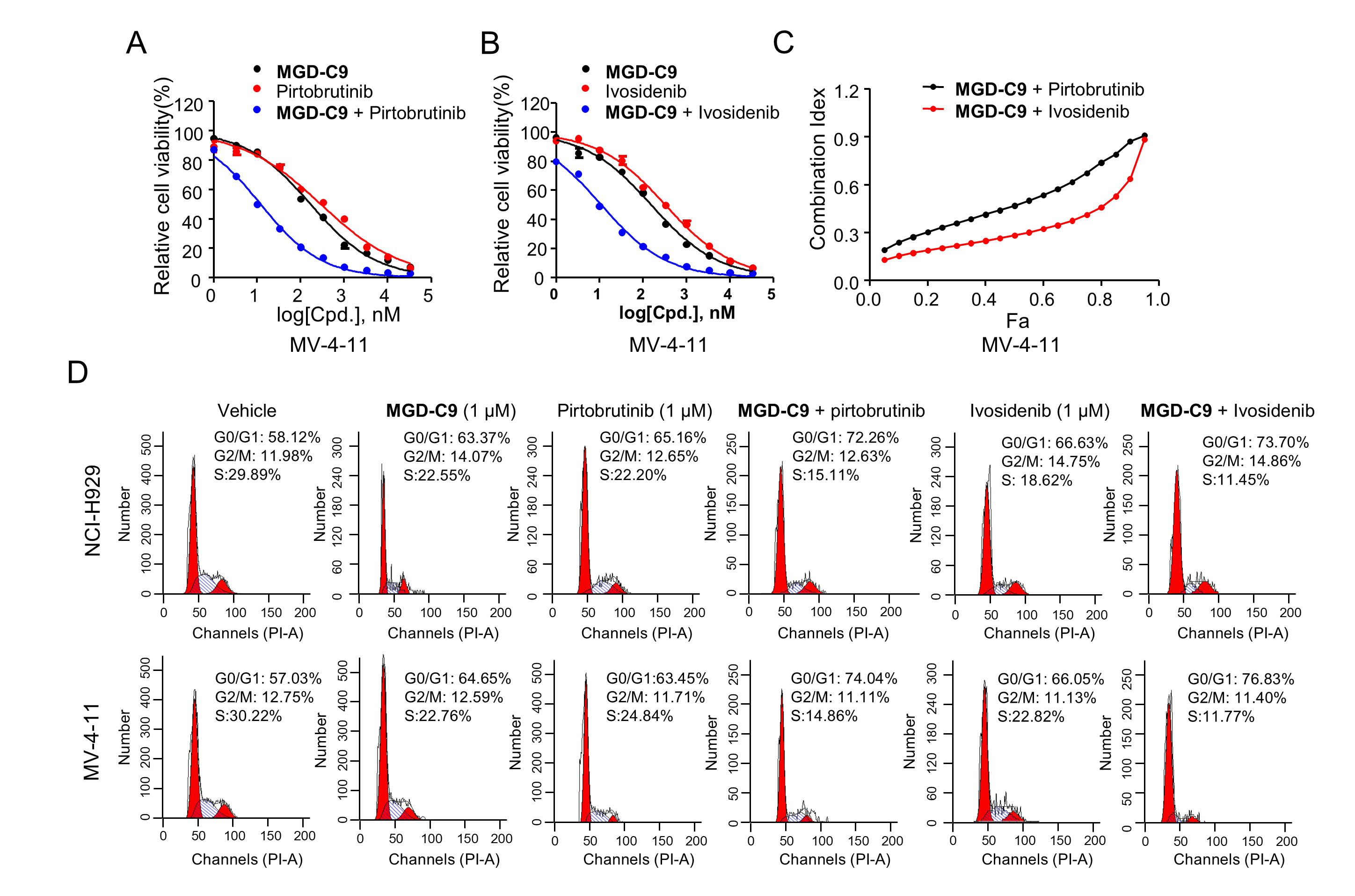

Supplement: Figure S4 [file OncolRes-33-65123-s006.TIF]
